# Hetero Diels–Alder Reactions with a Dicationic Urea Azine Derived Azo Dienophile and Their Use for the Synthesis of an Electron‐Rich Pentacene

**DOI:** 10.1002/chem.202001342

**Published:** 2020-09-07

**Authors:** Marco Werr, Elisabeth Kaifer, Hans‐Jörg Himmel

**Affiliations:** ^1^ Anorganisch-Chemisches Institut Ruprecht-Karls-Universität Heidelberg Im Neuenheimer Feld 270 69120 Heidelberg Germany

**Keywords:** azines, guanidines, ionic Diels–Alder reaction, pentacenes, reversible reactions

## Abstract

Herein, the first hetero Diels–Alder (DA) reactions with a stable, dicationic urea azine derived azo dienophile, synthesized by two‐electron oxidation of a neutral urea azine are reported. Several charged DA products were synthesized in good yield and fully characterized. The DA adduct of anthracene is in thermal equilibrium with the reactants at room temperature, and the reaction enthalpy and entropy were determined from the temperature‐dependent equilibrium constant. Furthermore, base addition to solutions of the pentacene DA product led to deprotonation, cleavage of the N−N bond, and formation of an electron‐rich 6,13‐bisguanidinyl‐substituted pentacene. The redox and optical properties of this new pentacene derivative were studied. Furthermore, the dication resulting from its two‐electron oxidation was synthesized and fully characterized. The results disclose a new elegant route to electron‐rich pentacene derivatives.

## Graphical abstract

**The use of oxidised urea azines** derived azo dienophiles in several Diels–Alder reactions is demonstrated. The DA product with anthracene is in equilibrium with its reactants at room temperature. Base‐induced N−N bond cleavage of the DA product with pentacene provides a new route to bisguanidinyl functionalized electron‐rich acenes (see scheme).

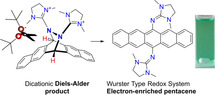

Cycloaddition reactions are among the most elegant coupling reactions in organic chemistry. Diene‐ene [4+2] cycloadditions, termed Diels–Alder (DA) reactions, lead to new six‐membered rings by formation of two new covalent bonds.^1^ The large scope of such reactions results from the variety of possible substrates and bonds (e.g., C−C, C−N) that could be formed. DA reactions are distinguished by their high atom economy, providing access to ring systems with high stereo‐ and regioselectivity.^2^ Hetero DA reactions lead in a single step to multi‐functionalized compounds, and are essential elements of synthetic strategies to build complex aromatics.^3^ Some DA reactions are reversible, allowing the liberation of the dienophile and diene components in a retro Diels–Alder (rDA) reaction initiated thermally,^4^ photochemically,^5^ or mechanochemically.^6^ Within the timely field of dynamic covalent chemistry (DCC),^7^ the DA reaction is used as one of the prime tools. However, there are only a few examples of rDA reactions for which the equilibrium could be varied fast near room temperature.^4^, ^8^

The use of an azo compound as a dienophile has marked the beginning of the DA reaction. In the year 1925, Diels et al. reported the reaction between cyclopentadiene and ethyl azodicarboxylate (**a**) yielding a six‐membered bridged heterocyclic product (Scheme 1).^9^ In the meantime, several azo compounds were applied as electron‐poor dienophiles in DA reactions.3a, 10 Popular examples are the already mentioned azodicarboxylic acid esters^11^ (**a**), as well as 4‐phenyl‐1,2,4‐triazoline‐3,5‐dione (PTAD)^12^ (**b**). The use of the dienophiles azobisformamidine^13^ (**c**) and dimethyldiazenium bromide^14^ (**d**) is hampered by their high reactivity and instability, and the susceptibility of the DA products to follow‐up reactions.^14^, ^15^ The electron‐deficient character of the azo group could be increased through induction or resonance effects, leading to higher reactivity in a normal electron‐demand DA reaction.^16^ Thus, the reactivity should decrease in the row **d>b>a>c**.3a However, when the electron deficit of the azo dienophile increases, redox reactions might compete with the DA reaction.3a, 10 Cis‐azo dienophiles, such as PTAD, exhibit higher reactivity compared to *trans*‐azo dienophiles (e.g., **a**),^17^ partially due to the reduced steric hindrance towards dienes with bulky groups.3a

**Scheme 1:**
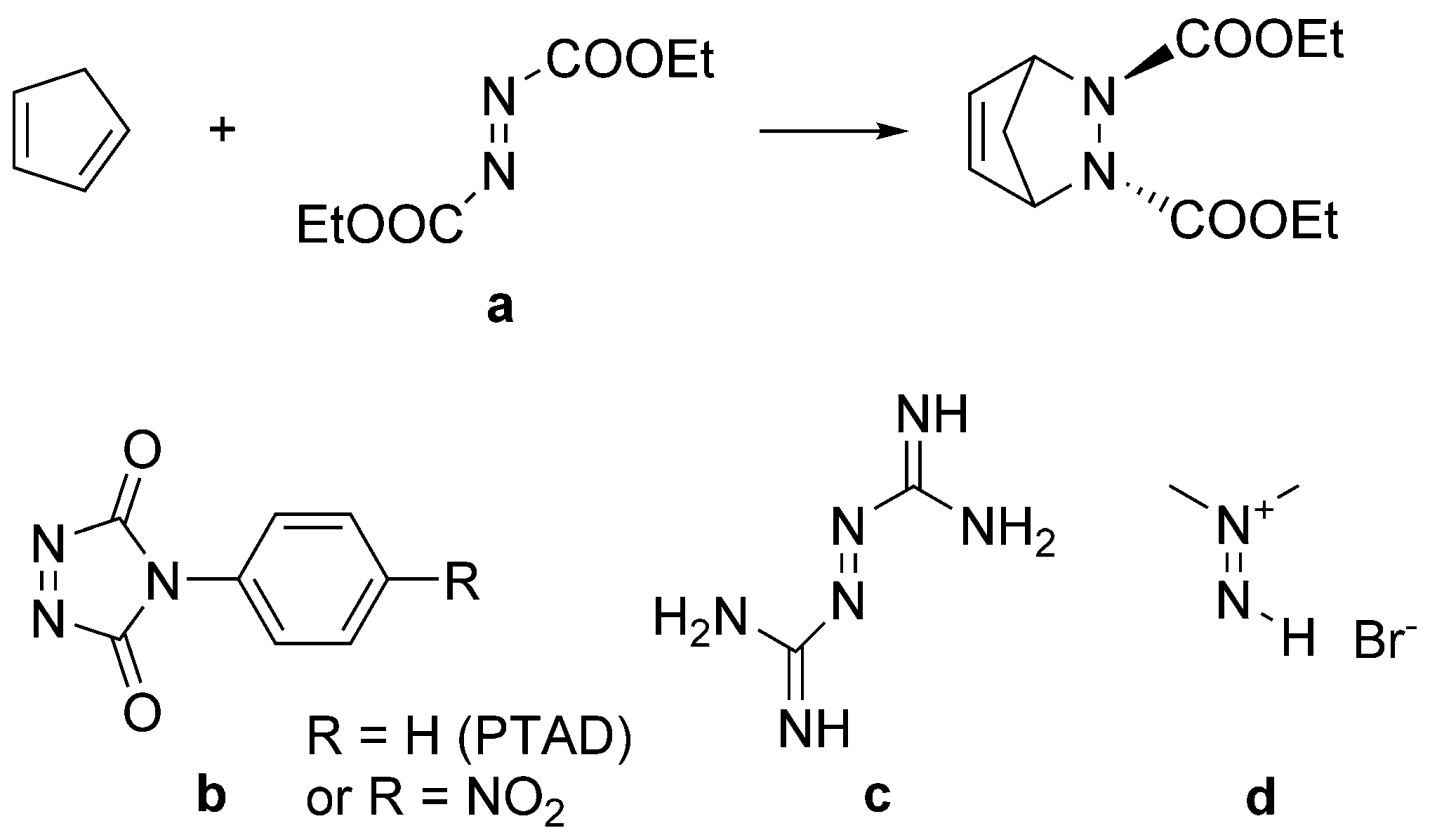
Selected known azo dienophiles **a**–**d** for hetero‐DA reactions.

Ionic DA reactions, involving cations or anions as diene or dienophile component, are quite rare in chemistry,^18^ and reactions with cationic species prevail.18e, 19 According to theoretical studies,18d, 18e, 20 ionic DA reactions follow different mechanisms than DA reactions of neutral reactants. Recent theoretical work by Domingo et al. showed that the rates of DA reactions increase with polarity and charge;18e, 20b hence, ionic DA reactions proceed fast already at low temperatures.

Based on our long‐term experience with redox‐active guanidines,^21^, ^22^ we herein report the use of the dication **1** (Scheme 2) as a new dienophile in ionic DA reactions, providing the first examples for hetero DA reactions with a dicationic azo dienophile in reaction with nonpolar dienes, leading to dicationic (bridged) heterocycles.

**Scheme 2:**
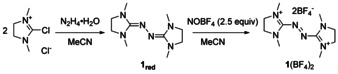
Synthesis of the dienophile **1**(BF_4_)_2_.

The anthracene DA product is in a thermal equilibrium with its reactants at room temperature. Furthermore, we disclose an elegant new route to the first bisguanidinyl‐substituted pentacene by N−N bond cleavage initiated by deprotonation of the DA product. Compound **1**(BF_4_)_2_ is conveniently accessible through oxidation of **1_red_** with NOBF_4_ (93 % yield).22e It is thermally robust and does not react with dioxygen, but is water sensitive. It is worth mentioning that **1_red_** is (structurally) related to azoimidazolium dyes, which have lately been used to form N‐heterocyclic carbene (NHC)‐derived nitrogen‐centered radicals and biradicals, as well as mesoionic carbene ligands (Azo‐MICs).^23^

The DA products **2**(BF_4_)_2_–**5**(BF_4_)_2_ (Scheme 3) were obtained in good yield by reaction of **1**(BF_4_)_2_ with the corresponding dienes in acetonitrile at room temperature. Conversion could be followed by the vanishing red colour of the dienophile. Under the applied conditions (see the Supporting Information), reaction with cyclopentadiene is completed in about one minute and with 1,3‐dimethylbutadiene in a few minutes. Relatively long reaction times of one–three days are required for the acenes due to their low solubility in acetonitrile (the reaction mixtures are suspensions).

**Scheme 3:**
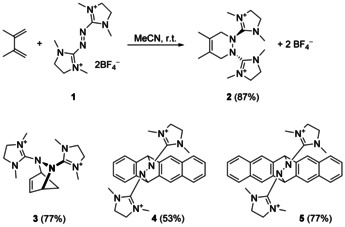
DA reactions of **1**(BF_4_)_2_ with electron‐rich dienes leading to compounds **2**–**5**. Quoted yields refer to the isolated pure compounds.

The pure colourless compounds **2**(BF_4_)_2_–**5**(BF_4_)_2_ are soluble in acetonitrile and stable under ambient conditions. Their structures, derived from SCXRD, are shown in Figure 1 (see the Supporting Information for details). The NN bond changes from a single to a double bond upon oxidation of **1_red_** to the dication **1** (1.416(1) Å in **1_red_** and 1.259(3) Å in **1**(BF_4_)_2_). In the DA products, it is converted back to a single bond (1.405(4), 1.438(5), 1.447(2), and 1.449(2) Å in **2**, **3**, **4**, and **5**, respectively). On the other hand, the imino C=N double bonds of **1_red_** that are converted to single bonds upon oxidation to **1**, remain single bonds in the DA product. Also, the other C−N bonds, that shorten upon oxidation, keep their short distances in the DA products. All these changes are in line with the Lewis structures in Scheme 3. In the solid state, **3** and **4** are chiral due to the *trans*‐type conformation of the guanidinyl groups. The crystal data of **3**(BF_4_)_2_ showed only one enantiomer of **3**, whereas **4**(BF_4_)_2_ crystallized as racemic mixture.

**Figure 1:**
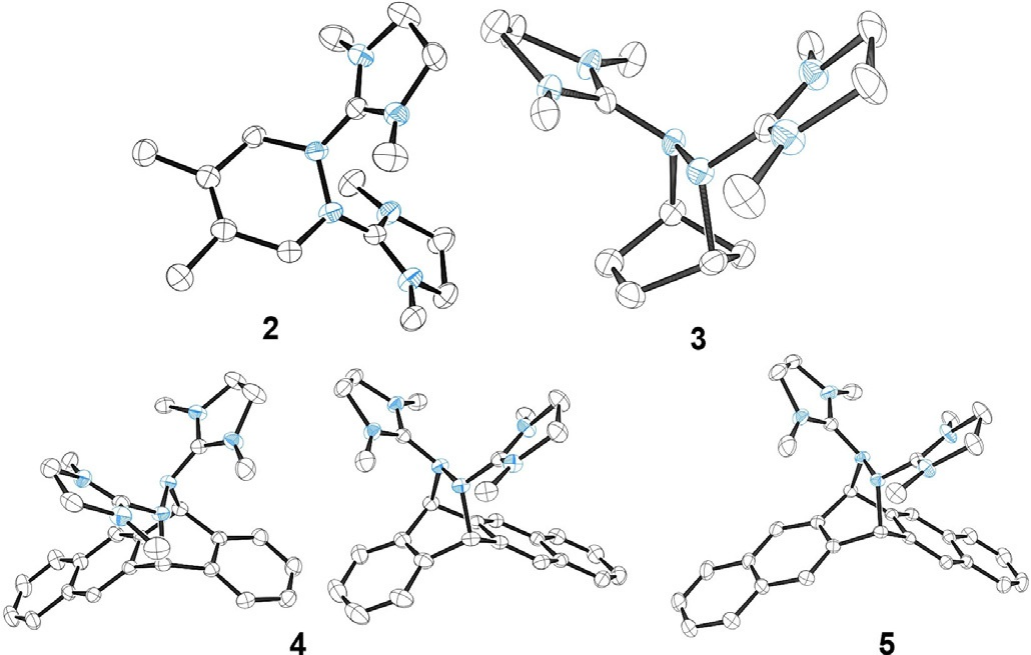
Illustration of the structures of the dications in the DA products **2**(BF_4_)–**5**(BF_4_)_2_ in the solid state (only one of the two independent molecules is shown for **2** and **3**, for details and bond parameters see the Supporting Information). Counterions (BF_4_^−^) and hydrogen atoms are omitted for clarity. Displacement ellipsoids drawn at the 50 % probability level.

In case of the DA reaction of anthracene with **1**(BF_4_)_2_ in MeCN, the ^1^H NMR spectra indicated the presence of a temperature‐dependent equilibrium between reactants and DA product **6**(BF_4_)_2_ (Figure 2). At room temperature, 32 % conversion was obtained, decreasing to 9 % at 55 °C and increasing to 60 % at −40 °C. This dynamic equilibrium resembles that reported by Lehn et al. for the reactions of PTAD with anthracene and derivatives, that also were found to be room‐temperature reversible.^4^ This behaviour is special, because most of the reported retro DA reactions require high temperatures.^24^ Thus, our results support the conclusion of Lehn et al. that retro DA is facilitated for azo dienophiles due to the lower CN versus CC single‐bond energy.

**Figure 2:**
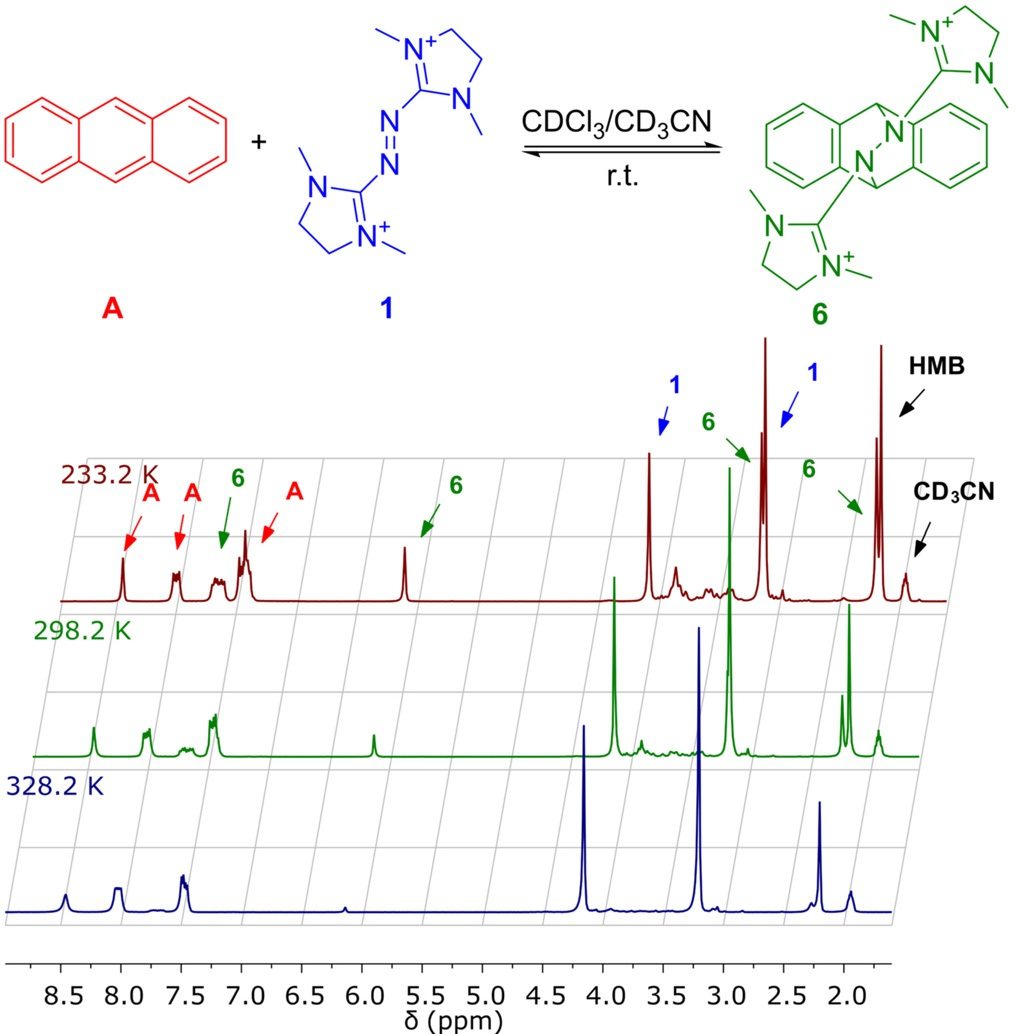
Variable‐temperature ^1^H NMR spectra (199.87 MHz, CDCl_3_/CD_3_CN 1:1) recorded for the equilibrium between the DA adduct **6** and the reactants, anthracene (**A**), and **1** (HMB=hexamethylbenzene).

Although the opposite polarities of the diene and dienophile components in our reaction might hamper an analysis by NMR spectroscopy,^25^ equilibrium constants were obtained (*K*_eq_=15.3±1.9 m ^−1^ at 25 °C, 79.9±9.8 m ^−1^ at −40 °C and 2.7±0.3 m ^−1^ at +55 °C) from ^1^H NMR signal integration at different temperatures in CD_3_CN/CDCl_3_ (1:1) solvent mixtures to which hexamethylbenzene (8.14 mm) was added as internal standard (see the Supporting Information). An analysis (see the Supporting Information for details) for the region 25–55 °C, gave Δ*H*=−47±5 kJ mol^−1^ and Δ*S*=−135±15 J mol^−1^ K^−1^. DFT calculations (B3LYP+D3/def2‐TZVP+COSMO; *ϵ*_r_=37.5) found Δ*G*=+30.7 kJ mol^−1^ and Δ*H*=−45.0 kJ mol^−1^, in line with the experimental values. A similar Δ*H* value of −44 kJ mol^−1^, but a lower Δ*S* value of −99 J mol^−1^ K^−1^ were reported by Lehn and co‐workers for DA reaction between the neutral azo dienophile PTAD and anthracene in CDCl_3_ solution.^4^ Clearly, a direct comparison is hampered by the different solvents, but the smaller entropy change for PTAD is most likely due to its fixed, pre‐oriented *cis*‐conformation.

The dication **5** appears to be a promising precursor for the formation of new electron‐rich 6,13‐guanidinyl‐pentacenes (Scheme 4) by cleavage of the N−N bond and removal of two protons. Several methods for N−N bond cleavage reactions of azines and hydrazones were reported by using reducing agents2c, 26 or catalytic hydrogenation.^27^ Cyclic voltammograms of **5**(BF_4_)_2_ in MeCN (see the Supporting Information) showed one irreversible reduction wave at about −1.45 V (−1.9 V vs. Fc^+^/Fc). After this reduction process has been passed through, typical oxidation and reduction waves of free **1_red_** appear, indicating that reduction of **5** leads to decomposition into **1_red_** and pentacene. In line with this result from electrochemistry, reactions of **5** with strong reductants leads to precipitation of pentacene. Therefore, N−N bond cleavage could not be initiated by reduction.

**Scheme 4:**
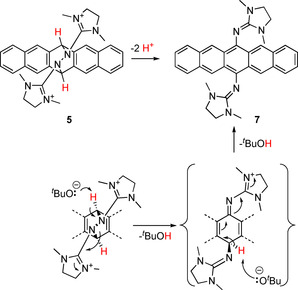
Base‐induced N−N bond cleavage of **5** leading to electron‐rich 6,13‐bisguanidinyl‐pentacene **7**.

Next, we tested if deprotonation of a bridge atom might provoke N−N bond cleavage (Scheme 4). Indeed, reaction with KO*t*Bu in THF gives a green solution and green residue, which was isolated by filtration and washed first with THF and then with water to remove the KBF_4_ by‐product, yielding neutral 6,13‐bisguanidinyl‐pentacene **7** (47 % yield). We propose concerted deprotonation and cleavage of the central N−N bond (Scheme 4), avoiding the formation of an intermediate carbanion that would violate Bredt's rule.^28^ Compound **7** is air and water stable (and also light stable under air exclusion). Its low solubility in all standard organic solvents prohibits its analysis by ^1^H NMR spectroscopy in solution and also the growth of crystals suitable for structural characterization through SCXRD. The UV/Vis spectrum (MeCN) is comparable to those of other pentacenes.^29^ The low‐energy band (*λ*_max_=718 nm) is assigned to the so‐called p‐band with partially resolved vibrational progression. Due to the superior electron‐donating effect of the guanidinyl groups, it is shifted bathochromic with respect to the parent pentacene (*λ*_max_=581 nm), 6,13‐diethylamino pentacene (*λ*_max_=606 nm), and 6,13‐dianisylamino pentacene (*λ*_max_=665 nm).^30^ The band at 453 nm in the spectrum of **7** is assigned to the α‐band showing vibrational progression (Δ*E*=1289 cm^−1^).29a In line with the experiments, TD‐DFT calculations (B3LYP+D3/def2‐TZVP) found two electronic transitions in the visible region at 822 nm (oscillator strength *f*=0.07, HOMO→LUMO excitation) and 411 nm (*f*=0.10, see the Supporting Information for details).

Although guanidines are generally strong bases,^31^ compound **7** was not protonated by water, indicative of a reduced basicity. Reaction of **7** with HOTf in THF gave magenta single crystals of (**7**+2 H)(OTf)_2_ (Figure 4 and Table 1). As was expected, protonation leads to hypsochromic shift of the Vis bands (to 607 and 431 nm) due to reduction of the donor ability of the guanidinyl groups (Figure 3). The p‐band now exhibits almost identical energy and vibrational progression as reported for 6,13‐bis(diethylamino)pentacene (607, 561 and 523 nm for (**7**+2H)^2+^ vs. 606, 562 and 524 nm for 6,13‐bis(diethylamino)pentacene).^30^

**Table 1:** Selected structural XRD parameters for protonated and oxidised **7** (bond lengths in Å; bond angles in °) together with values calculated (B3LYP/TZVP/D3) for **7**.

Parameter	**7** calcd	(**7**+2H^+^)(OTf)_2_	**8**
*d*(N1−C1)	1.391	1.435(2)	1.284(2)
*d*(N1−C12)	1.277	1.334(2)	1.348(2)
*d*(N2−C12)	1.391	1.330(2)	1.332(2)
*d*(N3−C12)	1.390	1.343(2)	1.320(3)
∢(C1‐N1‐C12)	126.4	126.5(2)	135.1(2)
∢(C11‐C1‐N1)	119.4	118.2(2)	126.4(2)
∢(C11‐C1‐N1‐C12)	77.1	79.8(2)	6.2(2)
∢(C1‐N1‐C12‐N2)	1.9	13.5(2)	95.4(2)

**Figure 3:**
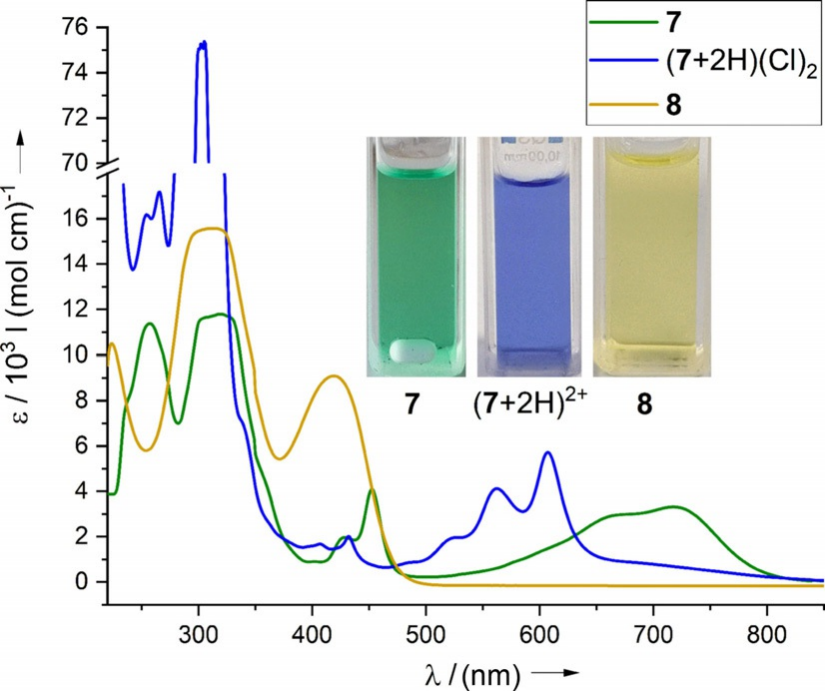
UV/Vis spectra of **7** (*c*=8.353×10^−5^ m) in CH_2_Cl_2_, (**7**+2 H)^2+^ (derived from reaction with HCl⋅Et_2_O; *c*=4.794×10^−5^ m) in MeCN and **8** (*c*=7.731×10^−5^ m) in MeCN. Colours of the solutions are shown as inlets.

The voltammogram of **7** in CH_2_Cl_2_ (see the Supporting Information) showed a single, quasi‐reversible redox event at *E*_1/2_=−0.65 V (*E*_Ox_=−0.56 V). The twofold oxidised pentacene derivative **8** was obtained by reaction of **7** with AgSbF_6_ in MeCN (Scheme 5). There are spectroscopic indications (UV/Vis and EPR) that chemical oxidation occurs in two one‐electron steps (see the Supporting Information for details). The salt **8**(SbF_6_)_2_ is thermally robust and, in difference to **7**, highly soluble in standard polar organic solvents. Its crystal structure (Figure 4 and Table 1) is consistent with the Lewis structure in Scheme 5. In the UV/Vis spectrum, yellow **8** shows one broad band in the Vis region (419 nm), which is similar in energy to the second lowest energy band observed for (**7**+2H)^2+^.

**Scheme 5:**
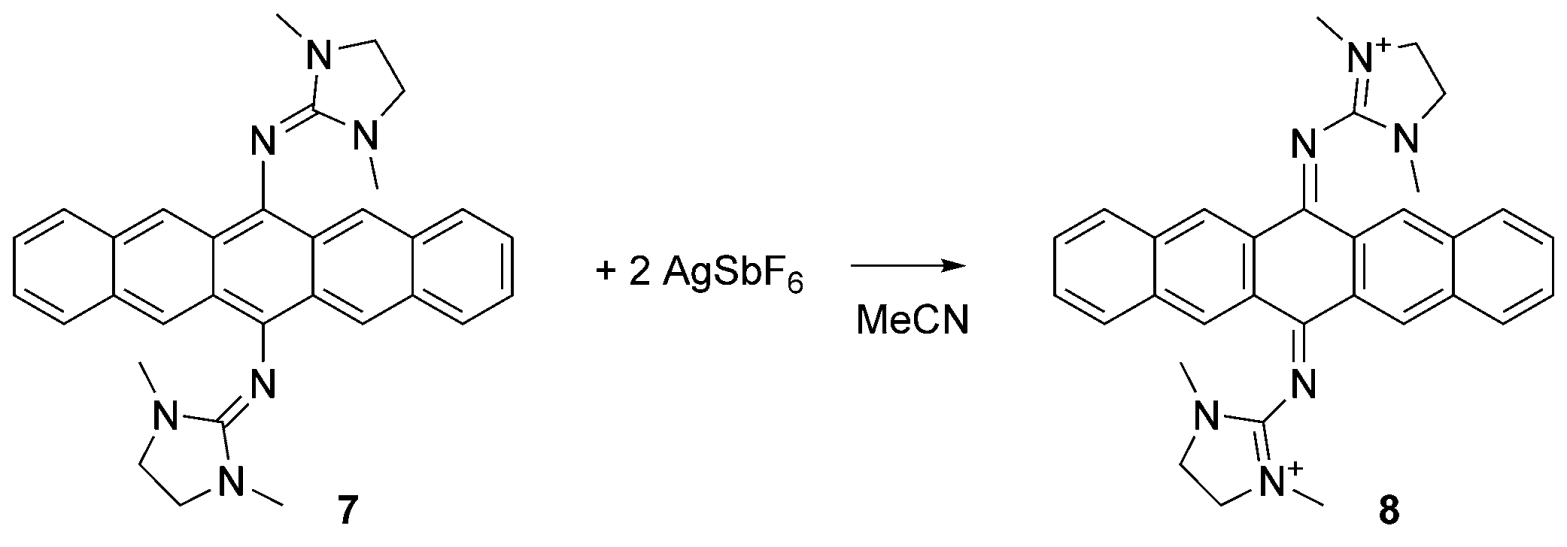
Synthesis of the dication **8** from two‐electron oxidation of **7**.

**Figure 4:**
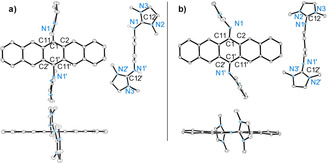
Molecular structures of (**7**+2 H^+^)(OTf)_2_ (a) and **8**(SbF_6_)_2_ (b; anions not shown, in case of (**7**+2 H^+^)(OTf)_2_ there are two molecules of MeCN per unit). Hydrogen atoms bound to carbon are omitted for clarity. Displacement ellipsoids drawn at the 50 % probability level. Selected bond parameters are given in Table 1.

In summary, oxidised urea azines were introduced as new potent dienophiles for ionic hetero Diels–Alder (DA) reactions. Several DA products were synthesized in good yield and fully characterized. In the case of anthracene, we find the DA reaction to be in a dynamic equilibrium at room temperature. The equilibrium constant and Δ*H* and Δ*G* values were estimated from quantitative NMR measurements at variable temperature. The reversible formation and cleavage of covalent bonds near room‐temperature makes the new dienophile to an interesting reagent in the field of dynamic covalent chemistry (DCC). In addition, the Diels–Alder product could be cleaved reversibly not only by temperature, but also electrochemically.

Furthermore, in an unprecedented reaction sequence, deprotonation of the pentacene DA product initiates cleavage of the N−N bond to give a new neutral, electron‐rich 6,13‐bisguanidinyl pentacene. Two‐electron oxidation gave the corresponding dication as a stable, highly soluble compound. Hence, a proficient new route to electron‐rich pentacene is disclosed. Similar work with other urea azine derived azo dienophiles, as well as base‐induced N−N cleavage reactions of the resulting DA products, are on the way.

## Conflict of interest

The authors declare no conflict of interest.

## Supporting information

As a service to our authors and readers, this journal provides supporting information supplied by the authors. Such materials are peer reviewed and may be re‐organized for online delivery, but are not copy‐edited or typeset. Technical support issues arising from supporting information (other than missing files) should be addressed to the authors.

Supplementary
