# Supplementary material for: Hetero Diels–Alder Reactions with a Dicationic Urea Azine Derived Azo Dienophile and Their Use for the Synthesis of an Electron‐Rich Pentacene
Source: Chemistry. 2020 Sep 7;26(54):12328–32. doi: 10.1002/chem.202001342 (PMC7589293; doi:10.1002/chem.202001342)
Supplement: Supplementary file 1 — Supplementary [file CHEM-26-12328-s001.pdf]

Chemistry–A European Journal

Supporting Information

Hetero Diels–Alder Reactions with a Dicationic Urea Azine Derived Azo Dienophile and Their Use for the Synthesis of an Electron-Rich Pentacene

Marco Werr, Elisabeth Kaifer, and Hans-Jörg Himmel*[a]

Table of Contents

No.	Title	Page
1	Experimental Procedures	2
2	Experimental Details and analytical data for all compounds	4
3	NMR analysis of the DA products	9
4	Room-temperature reversible hetero Diels-Alder reaction of **6**$(BF_4)_2$	10
5	Selected structural parameters of the DA products **1-5**	13
6	Cyclovoltammetric studies	14
7	Titration of **7** with $AgSbF_6$	17
8	EPR spectra	19
9	Computational data	21
10	Structural comparison of **7**	25
11	Results of the crystal structure determinations	27
12	1D- and (selected) 2D-NMR spectra of the described compounds	29
13	HR-Mass spectra	40
14	IR-spectra	46
15	Calculated minimum structures .xyz data	49
16	References	75

1. Experimental Procedures

General experimental details

If not otherwise stated, all synthetic work was carried out under an inert-gas atmosphere using the Schlenk technique. The used chemicals were obtained from common companies (Sigma-Aldrich, Acros Organics, Alfa Aesar, abcr GmbH and TCI (pentacene 99.999%, tetracene >97%)). Solvents were dried with an MBraun Solvent Purification System, degassed by three freeze-pump-thaw cycles and stored over molecular sieves prior to their use. Elemental analysis was carried out at the Microanalytical Laboratory of the University of Heidelberg. NMR spectra were recorded with Bruker DPX 200 (also VT-NMR), Bruker Avance II 400 or Bruker Avance III 600 spectrometers. HR-ESI spectra were recorded with a Bruker ApexQe hybrid 9.4 T FT-ICR spectrometer. IR spectroscopic measurements were performed on an FT-IR Biorad Merlin Excalibur FT 300 spectrometer. UV/Vis measurements relied on a Cary 5000 spectrometer. CV measurements were carried out at a Metrohm Autolab PGSTAT 204 potentiostate/galvanostate with an Ag/AgCl reference electrode. The curves were recorded at room temperature. CH_2Cl_2 or MeCN were used as solvent for the individual compounds ($c = 10^{-3}$ M), whereas $n$$Bu_4N(PF_6)$ (electrochemical grade (≥99.0%), Fluka) was employed as supporting electrolyte ($c = 0.1$ M). X-band EPR spectra were measured at room-temperature in a glass capillary with a Bruker ESP 300 E.

X-ray Crystallographic Study

Full shells of intensity data were collected at low temperature with a Nonius Kappa CCD diffractometer (Mo-K_α radiation, sealed X-ray tube, graphite monochromator, compound **2**$(BF_4)_2$) and Bruker D8 Venture, dual source (Mo- or Cu-K_α radiation, microfocus X-ray tube, Photon III detector, compounds **3**$(BF_4)_2$, **4**$(BF_4)_2$, **5**$(BF_4)_2$, (**7**+2H)$(OTf)_2$, and **8**). Data were processed with the standard Nonius and Bruker (SAINT, APEX3) software package.[1] Multiscan absorption correction was applied using the SADABS program.[2] The structures were solved by intrinsic phasing[3] and refined using the SHELXTL software package (Version 2014/6 and 2018/3).[4] Graphical handling of the structural data during solution and refinement were performed with OLEX2.[5] All non-hydrogen atoms were given anisotropic displacement parameters. Hydrogen atoms bound to carbon were input at calculated positions and refined with a riding model. Hydrogen atoms bound to nitrogen were located in difference Fourier syntheses and refined, either fully or with appropriate distance and/or symmetry.
Due to disorder and fractional occupancy, electron density attributed to solvent of crystallization (Acetonitrile) was removed from the structure of **2**$(BF_4)_2$ with the BYPASS procedure,[6] as implemented in PLATON (squeeze/hybrid).[7] Partial structure factors from the solvent masks were included in the refinement as separate contributions to F_{calc}.
Crystallographic data for the structures reported in this paper have been deposited in the Cambridge Crystallographic Data Centre (see **Chap. 11** for CCDC No.) These data can be obtained free of charge from The Cambridge Crystallographic Data Centre via www.ccdc.cam.ac.uk/data_request/cif.
Structures are visualized with ORTEP III.[8]

Details of quantum chemical calculations

Quantum-chemical (DFT) calculations were performed with the 7.3.1 suite of the TURBOMOLE program[9] using the B3LYP functional[10] together with the def2-TZVP basis set.[11] The RI (resolution identity) approximation[12] and multipole accelerated RI-J[13] was applied for structure optimization. All structures are stationary points on the energy potential surface as confirmed by frequency computations.[14] For the calculation of the thermodynamic properties unscaled harmonic vibrational frequencies were used. Time-dependent density functional theory (TD-DFT) computations were performed to obtain the electronic excitations.[15] The excitation energies for the first 40 states were calculated. Dispersion was included by the DFT-D3[16] approach developed by Grimme and co-workers. In the calculations of free Gibbs energies, the solvent effect was considered with the conductor-like screening model (COSMO)[17] by means of single-point calculations (ε_r = 37.50 for CH_3CN). The deviations between experimentally derived and calculated structures were analyzed with the program aRMSD[18] using default settings. Orbital visualization relied on the IboView program,[19] and the visualization of the optimized structures on the mercury program.[20]

References

[1] a) *DENZO-SMN*, Z. Otwinowski & W. Minor, Processing of X-ray Diffraction Data Collected in Oscillation Mode, Methods Enzymol. (1997), 276, Eds C. W. Carter, R. M. Sweet, Academic Press.; b) *SAINT*, Bruker AXS GmbH, Karlsruhe, Germany **2016**.

[2] a) G. M. Sheldrick, SADABS, Bruker AXS GmbH, Karlsruhe, Germany **2004**-**2014**; b) L. Krause, R. Herbst-Irmer, G. M. Sheldrick, D. Stalke, *J. Appl. Cryst.* **2015**, *48*, 3.

[3] a) G. M. Sheldrick, SHELXT, *Program for Crystal Structure Solution*, University of Göttingen, Germany **2014**-**2018**; b) G. M. Sheldrick, Acta Cryst. **2015**, *A71*, 3.

[4] a) G. M. Sheldrick, *SHELXL-20xx*, University of Göttingen and Bruker AXS GmbH, Karlsruhe,Germany **2012**-**2018**; b) W. Robinson, G. M. Sheldrick in: N. W. Isaaks, M. R. Taylor (eds.) “*Crystallographic Computing 4*“, Ch. 22, IUCr and Oxford University Press, Oxford, UK, **1988**; c) G. M. Sheldrick, *Acta Cryst.* **2008**, *A64*, 112; d) G. M. Sheldrick, *Acta Cryst.* **2015**, *C71*, 3.

[5] O. V. Dolomanov, L. J. Bourhis, R. J. Gildea, J. A. K. Howard, H. Puschmann, OLEX2: A complete structure solution, refinement and analysis program, *J. Appl. Cryst.* **2009**, *42*, 339.

[6] a) P. v. d. Sluis, A. L. Spek, *Acta Cryst.* **1990**, *A46*, 194; b) A. L. Spek, *Acta Cryst.* **2015**, *C71*, 9.

[7] a) A. L. Spek, *PLATON*, Utrecht University, The Netherlands; b) A. L. Spek, *J. Appl. Cryst.* **2003**, *36*, 7.

[8] L. J. Farrugia, *J. Appl. Cryst.* **2012**, *45*, 849.

[9] a) F. Furche, R. Ahlrichs, C. Hättig, W. Klopper, M. Sierka, F. Weigend, *WIREs Comput. Mol. Sci.* **2014**, *4*, 91; b) *TURBOMOLE*, vers. 7.3.1 **2019**, a development of the University of Karlsruhe and Forschungszentrum Karlsruhe GmbH, **1989**-**2007**, TURBOMOLE GmbH, since 2007.

[10] a) A. D. Becke, *J. Chem. Phys.* **1993**, *98*, 5648; b) C. Lee, W. Yang, R. G. Parr, *Phys. Rev.* **1988**, *37*, 785.

[11] A. Schäfer, H. Horn, R. Ahlrichs, *J. Chem. Phys.* **1992**, *97*, 2571.

[12] O. Treutler, R. Ahlrichs, *J. Chem. Phys.* **1995**, *102*, 346.

[13] M. Sierka, A. Hogekamp, R. Ahlrichs, *J. Chem. Phys.* **2003**, *118*, 9136.

[14] P. Deglmann, K. May, F. Furche, R. Ahlrichs, *Chem. Phys. Lett.* **2004**, *384*, 103.

[15] R. Bauernschmitt, M. Häser, O. Treutler, R. Ahlrichs, *Chem. Phys. Lett.* **1997**, *264*, 573.

[16] S. Grimme, *J. Comput. Chem.* **2004**, *25*, 1463.

[17] A. Klamt; G. Schüürmann, *J. Chem. Soc. Perkin Trans.* **1993**, *2*, 799.

[18] A. Wagner, H.-J. Himmel, *J. Chem. Inf. Model* **2017**, *57*, 428.

[19] a) G. Knizia, *J. Chem. Theory Comput.* **2013**, *9*, 4834; b) G. Knizia, J.E.M.N. Klein, *Angew. Chem. Int. Ed.* **2015,** *54*, 5518.

[20] Mercury 4.0: *from visualization to analysis, design and prediction* C. F. Macrae, I. Sovago, S. J. Cottrell, P. T. A. Galek, P. McCabe, E. Pidcock, M. Platings, G. P. Shields, J. S. Stevens, M. Towler and P. A. Wood, *J. Appl. Cryst.* **2020***, 53,* 226.

2. Experimental Details and analytical data for all compounds

Synthesis of the Diels-Alder dienophile 1(BF$_4$)$_2$

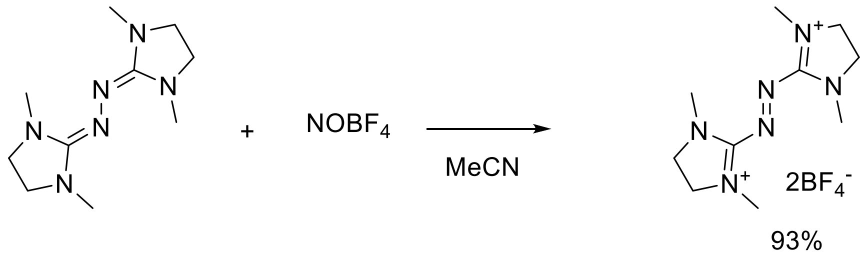

Using an upscaled, slightly modified procedure we achieved higher yields compared with the previously published procedure.[1] 1,2-bis(1,3-dimethylimidazolidin-2-ylidene)-hydrazine[1] (750 mg, 3.34 mmol, 1 eq.) and 1.00 g of NOBF$_4$ (1.00 g, 8.69 mmol, 2.6 eq.) are mixed in a Schlenk tube and dissolved in acetonitrile (30 mL), resulting in an immediate colour change to red-brown. While stirring, the reaction vessel is evacuated numerous times (at least 8 times) until the solvent started to boil and then purged with Ar to remove traces of NO. Then, the reaction mixture is stirred 16 h under slightly reduced pressure. Subsequently, all volatiles are removed *in vacuo* and the residue is dissolved in acetonitrile (8 mL) and crystallized by diffusion of diethyl ether (30 mL) from the gas-phase into the solution. The product **1**(BF$_4$)$_2$ is obtained as red needles (1.24 g, 3.11 mmol, 93%).

^{1}H NMR (199.87 MHz, CD$_3$CN, 298 K): δ = 4.13 (s, 8 H, CH$_2$), 3.15 (s, 12 H, CH$_3$) ppm.
Further analytical data can be found in the literature.[2]

General procedure for the dicationic hetero Diels-Alder reaction of 1(BF$_4$)$_2$ with different dienes

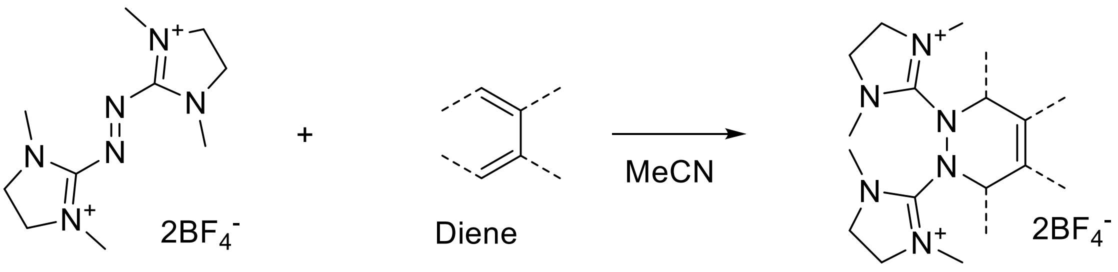

1(BF$_4$)$_2$

The dicationic hetero Diels-Alder reaction is carried out by adding the dienophile **1**(BF$_4$)$_2$ (1 eq.) and corresponding diene (1 eq.) in a Schlenk tube. After the addition of acetonitrile, the reaction mixture is stirred for the given reaction time (reaction progress can be followed by decolorization of the red reaction mixture). After the work-up, XRD suitable single crystals are grown.

2,3-Dimethylbutadiene Diels-Alder product, 2(BF$_4$)$_2$

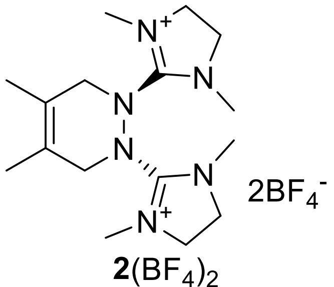

2(BF$_4$)$_2$

The reaction is carried out by the general procedure with **1**(BF$_4$)$_2$ (56 mg, 141 µmol, 1 eq.) and 2,3-dimethylbutadiene (16 µl, 141 µmol, 1 eq.) in MeCN (3 mL) for 10 min. Subsequently all volatiles are removed *in vacuo* and the residue is washed with Et$_2$O (3x3 mL) giving the title compound as a pale-white powder (59 mg, 123 µmol, 87%). XRD suitable single crystals are obtained by diffusion of Et$_2$O from the gas-phase into a MeCN solution.

^{1}H NMR (399.89 MHz, CD_3CN, 294 K): δ = 3.97 (4 H, bs, DA-bridge-CH_2), 3.78 (bs, 8 H, CH_2-gua.), 3.04 (s, 12 H, CH_3-gua.), 1.71 (bs, 6 H, CH_3-diene) ppm.
^{13}C{^{1}H} NMR (100.55 MHz, CD_3CN, 295 K): δ = 161.70 (Cq-gua.), 122.84 (Cq-diene), 53.55 (CH_2-DA-bridge), 51.13 (CH_2-gua), 36.43 (CH_3-gua), 15.57 (CH_3-diene) ppm.
MS (ESI^+, MeCN): m/z = 153.12 (100%, $[M{-}2BF_4^-]^{2+}$).
Elemental Analysis ($C_{16}H_{30}B_2F_8N_6$, *MW*: 480.07 g/mol): calcd. C 40.03, H 6.30, N 17.51; found C 39.50, H 5.79, N 18.01

Cyclopentadiene Diels-Alder product, 3(BF_4)$_2$

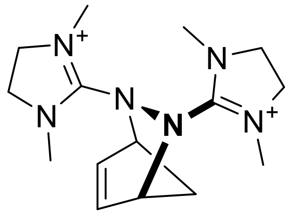

3(BF_4)$_2$

The reaction is carried out by the general procedure with **1**(BF_4)$_2$ (61 mg, 153.3 µmol, 1 eq.) and freshly distilled cyclopentadiene (13 µL, 153.3 µmol, 1 eq.) in MeCN (3 mL) for 5 min. Subsequently all volatiles are removed *in vacuo* and the residue is washed with Et_2O (3x3 mL) giving the title compound as a pale-yellowish powder (55 mg, 119 µmol, 77%). XRD suitable single crystals are obtained by diffusion of Et_2O from the gas-phase into a MeCN solution.

^{1}H NMR (399.89 MHz, CD_3CN, 294 K): δ = 6.91-6.84 (2 H, m, RHC=CHR), 5.28-5.26 (m, 1 H, $CH_{DA\text{-}bridge}$), 5.23-5.22 (m, 1 H, $CH_{DA\text{-}bridge}$), 3.93-3.83 (m, 4 H, $CH_{2,gua.}$), 3.67-3.61 (m, 4 H, $CH_{2,gua.}$), 3.15 (s, 3 H, $CH_{3,gua.}$), 3.12 (bs, 6 H, $CH_{3,gua.}$), 2.91 (s, 3 H, $CH_{3,gua.}$), 2.06-1.98 (m, 2 H, $CH_{2,diene}$) ppm.
^{13}C{^{1}H} NMR (100.55 MHz, CD_3CN, 295 K): δ = 160.04 ($Cq_{gua.}$), 159.69 ($Cq_{gua.}$), 140.86 (RCH=CHR), 137.74 (RCH=CHR), 75.82 ($CH_{DA\text{-}bridge}$), 72.32 ($CH_{DA\text{-}bridge}$), 52.55 ($CH_{2,gua.}$), 49.57 ($CH_{2,diene}$), 49.38 ($CH_{2,gua.}$), 36.60 ($CH_{3,gua.}$), 35.59 ($CH_{3,gua.}$) ppm.
IR (KBr): $\tilde{\nu}$ = 2949 (w), 2898 (w), 1611 (s), 1559 (s), 1457 (w), 1421 (m), 1379 (m), 1306 (m), 1055 (vs), 922 (w), 859 (m), 782 (w), 748 (w), 689 (w), 645 (w), 522 (m) cm^{-1}.
HR-MS (ESI^+, MeCN): m/z calcd for $C_{15}H_{26}BF_4N_6^+$ $[M{-}BF_4^-]^+$ = 377.2243, found m/z = 377.2243.
Elemental Analysis ($C_{15}H_{26}B_2F_8N_6$, *MW*: 464.02 g/mol): calcd. C 38.83, H 5.56, N 18.11; found C 39.20, H 6.21, N 18.89.

Tetracene Diels-Alder product, 4(BF_4)$_2$

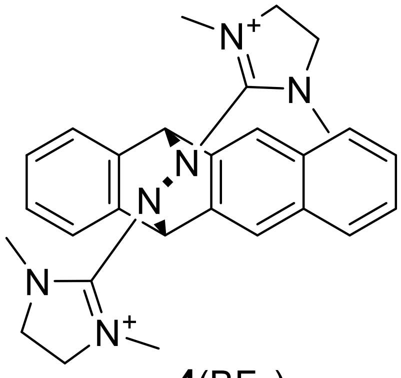

4(BF_4)$_2$

The reaction is carried out by the general procedure with **1**(BF_4)$_2$ (400 mg, 1.01 mmol, 1 eq.) and tetracene (230 mg, 1.01 mmol, 1 eq.) under exclusion of light in MeCN (30 mL) for 36 h. Subsequently the reaction mixture is filtered under Ar, all volatiles are removed *in vacuo* from the filtrate and the residue is washed with Et_2O (3x3 mL) and ice cooled acetone (2x5 mL), yielding the title compound as an off-white powder (337 mg, 538 µmol, 53%). XRD suitable single crystals are obtained by diffusion of Et_2O from the gas-phase into a MeCN solution.

^{1}H NMR (399.89 MHz, CD_3CN, 295 K): *δ* = 8.24 (s, 1 H, $CH_{arom.}$), 8.12 (s, 1 H, $CH_{arom.}$), 7.99-7.96 (m, 2 H, $CH_{arom.}$), 7.81-7.79 (m, 1 H, $CH_{arom.}$), 7.73-7.71 (m, 1 H, $CH_{arom.}$), 7.66-7.63 (m, 2 H, $CH_{arom.}$), 7.52-7.50 (m, 2 H, $CH_{arom.}$), 6.29 (s, 1 H, CH-DA-bridge), 6.28 (s, 1 H, CH-DA-bridge), 3.93-3.36 (m, 8 H, CH_2-gua.), 3.25 (s, 3 H, CH_3-gua.), 3.24 (s, 3 H, CH_3-gua.), 2.29 (s, 3 H, CH_3-gua.), 2.21 (s, 3 H, CH_3-gua.) ppm.
^{13}C{^{1}H} NMR (100.55 MHz, CD_3CN, 296 K): *δ* = 159.98 ($Cq_{gua.}$), 137.98 ($Cq_{arom.}$), 136.54 ($Cq_{arom.}$), 133.93 ($Cq_{arom.}$), 133.93 ($Cq_{arom.}$), 133.51 ($Cq_{arom.}$), 132.66 ($Cq_{arom.}$), 131.07 ($CH_{arom.}$), 130.50 ($CH_{arom.}$), 129.70 ($CH_{arom.}$), 129.61 ($CH_{arom.}$), 128.94 ($CH_{arom.}$), 128.81 ($CH_{arom.}$), 126.71 ($CH_{arom.}$), 126.34 ($CH_{arom.}$), 125.67 ($CH_{arom.}$), 125.14 ($CH_{arom.}$), 66.08 ($CH_{DA\text{-}bridge}$), 51.93 ($CH_{2,gua.}$), 49.11 ($CH_{2,gua.}$), 35.75 ($CH_{3,gua.}$), 35.68 ($CH_{3,gua.}$), 35.56 ($CH_{3,gua.}$), 35.39 ($CH_{3,gua.}$) ppm.
IR (KBr): 3040 (sh), 2953 (w), 2895 (w), 1718 (m), 1611 (vs), 1558 (s), 1507 (w), 1477 (w), 1466 (w), 1457 (w), 1419 (w), 1411 (w), 1374 (m), 1308 (m), 1218 (w), 1056 (vs), 935 (w), 859 (w), 835 (w), 818 (w), 772 (m), 761 (m), 734 (w), 613 (w), 699 (w), 686 (w), 647 (w), 627 (w), 580 (w), 567 (w), 552 (w), 522 (w), 488 (w) cm^{-1}.
HR-MS (ESI^+, MeCN): m/z calcd for $C_{28}H_{32}BF_4N_6^+$ $[M-BF_4^-]^+$ = 539.2712, found m/z = 539.2720.
Elemental Analysis ($C_{28}H_{32}B_2F_8N_6$, *MW*: 626.12 g/mol): calcd. C 53.71, H 5.15, N 13.42; found C 53.98, H 5.30, N 13.22.

Pentacene Diels-Alder product 5(BF$_4$)$_2$

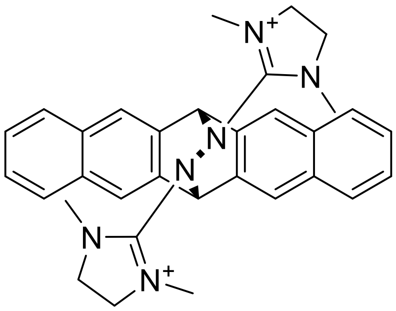

5(BF_4)$_2$

The reaction is carried out by the general procedure with **1**(BF_4)$_2$ (400 mg, 1.01 mmol, 1 eq.) and pentacene (280 mg, 1.01 mmol, 1 eq.) under exclusion of light in MeCN (30 mL) for 72 h. Subsequently the reaction mixture is filtered under Ar, all volatiles are removed *in vacuo* from the filtrate, and the residue is washed with Et_2O (3x3 mL) and ice cooled acetone (2x5 mL), yielding the title compound as an off-white powder (527 mg, 780 µmol, 77%). XRD suitable single crystals are obtained by diffusion of Et_2O from the gas-phase into a MeCN solution.

^{1}H NMR (600.13 MHz, CD_3CN, 295 K): *δ* = 8.28 (s, 2 H, $CH_{arom.}$), 8.18 (s, 2 H, $CH_{arom.}$), 8.01-7.99 (m, 4 H, $CH_{arom.}$), 7.67-7.63 (m, 4 H, $CH_{arom.}$), 6.41 (s, 2 H, $CH_{DA\text{-}bridge}$), 3.96-3.90 (m, 2 H, $CH_{2,gua.}$), 3.86-3.80 (m, 2 H, $CH_{2,gua.}$), 3.66-3.61 (m, 2 H, $CH_{2,gua.}$), 3.45-3.40 (m, 2 H, $CH_{2,gua.}$), 3.29 (s, 6 H, $CH_{3,gua.}$), 2.26 (s, 6 H, $CH_{3,gua.}$) ppm.
^{13}C{^{1}H} NMR (150.90 MHz, CD_3CN, 296 K): *δ* = 159.95 ($Cq_{gua.}$), 134.12 ($Cq_{arom.}$), 133.83 ($Cq_{arom.}$), 133.74 ($Cq_{arom.}$), 132.60 ($Cq_{arom.}$), 129.73 ($CH_{arom.}$), 129.64 ($CH_{arom.}$), 128.98 ($CH_{arom.}$), 128.85 ($CH_{arom.}$), 126.34 ($CH_{arom.}$), 125.22 ($CH_{arom.}$), 66.12 ($CH_{DA\text{-}bridge}$), 51.98 ($CH_{2,gua.}$), 49.18 ($CH_{2,gua.}$), 35.83 ($CH_{3,gua.}$), 35.67 ($CH_{3,gua.}$) ppm.
IR (KBr): $\tilde{\nu}$ = 3030 (w), 2957 (w), 2892 (w), 1616 (vs), 1558 (s), 1505 (w), 1457 (w), 1411(w), 1375 (m), 1309 (s), 1231 (w), 1168 (w), 1052 (vs), 896 (w), 861 (m), 846 (m), 771 (m), 761 (m), 751 (m), 728 (m), 691 (w), 646 (w), 587 (w), 568 (w), 559 (w), 545 (w), 522 (w), 489 (w), 478 (m) cm^{-1}.
HR-MS (ESI^+, MeCN): m/z calcd for $C_{32}H_{34}BF_4N_6^+$ $[M-BF_4^-]^+$ = 589.2869, found m/z = 589.2868.
Elemental Analysis ($C_{32}H_{34}B_2F_8N_6$, *MW*: 676.27 g/mol): calcd. C 56.83, H 5.07, N 12.43; found C 56.23, H 5.25, N 12.97.

6,13-Bis(*N*,*N'*-dimethylethyleneguanidinyl)-pentacene, 7

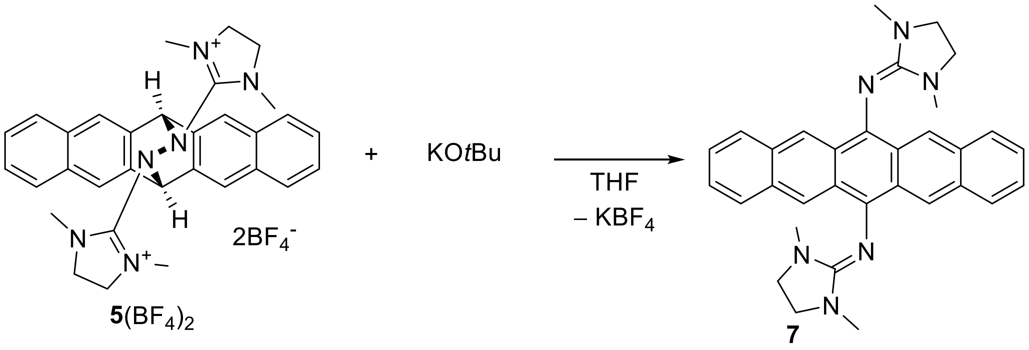

In a Schlenk tube, **5**$(BF_4)_2$ (100 mg, 147.9 µmol, 1 eq.) and KO*t*Bu (34 mg, 303.1 µmol, 2.05 eq.) are mixed. After addition of THF (4 mL) a green suspension formed, that is stirred for 22 h under exclusion of light. Subsequently the reaction mixture is filtered and the residue is dried. The raw product is washed under Ar with THF (3x4 mL). Then, it is suspended in DCM and washed with water (3x4 mL) (filtering under Ar) to remove KBF_4. Unfortunately, it is not possible to remove all KBF_4 due to its low solubility. Please note that the reaction does not work with LiO*t*Bu in place for KO*t*Bu, indicating that the formation of un-soluble KBF_4 might be an important driving force. The title compound is obtained as an emerald coloured powder (35 mg, 70 µmol, 47%).

IR (KBr): $\tilde{\nu}$ = 3046 (w), 2951 (w), 2845 (w), 1624 (vs), 1613 (vs), 1483 (m), 1431 (m), 1414 (m), 1383 (s), 1267 (m), 1236 (w), 1197 (w), 1135 (m), 1050 (m), 1011 (m), 993 (w), 955 (s), 877 (m), 792 (w), 759 (m), 700 (w), 621 (w), 571 (w), 514 (w), 466 (m) cm^{-1}.
UV-Vis (CH_2Cl_2, c = $8.353\cdot10^{-5}$ mol L^{-1}): λ_{max} (ε, 10^3 L mol^{-1} cm^{-1}) = 718 (3.3), 675 (2.98), 453 (4.1), 428 (2.0), 318 (11.8), 257 (11.4) nm.
HR-MS (ESI^+, CH_2Cl_2): m/z calcd for $C_{32}H_{33}N_6^+$ $[M+H]^+$ = 501.2761, found m/z = 501.2762.
Elemental Analysis ($C_{32}H_{32}N_6\cdot0.4\ KBF_4$, *MW*: 500.65 g/mol): calcd. C 69.75, H 5.85, N 15.25; found C 70.19, H 6.06, N 14.66.

6,13-Bis(*N*,*N'*-dimethylethyleneguanidinium)pentacene bistriflate/chloride, (7+2H)(OTf/Cl)$_2$

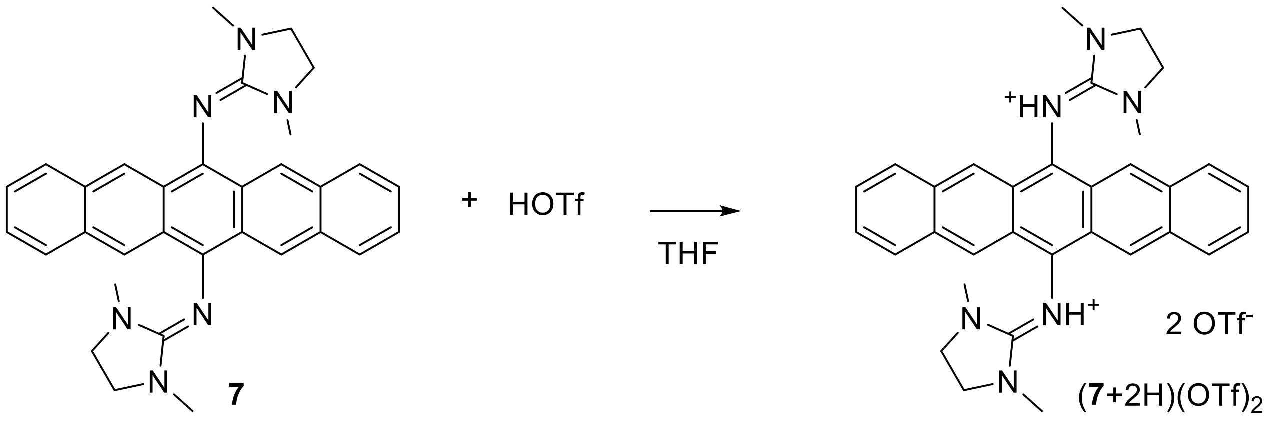

In a Schlenk tube **7** (15 mg, 30 µmol, 1 eq.) is suspended in THF (4 mL) under the absence of light trifluoromethanesulfonic acid (6 µL, 63 µmol, 2.1 eq.) is added. The reaction mixture immediately changes colour from a green suspension to a deep blue purple solution. It is stirred for a period of 5 min. Then all volatiles are removed *in vacuo* and the residue is washed with Et_2O (2x3 mL). For the growth of XRD suitable single crystals, the purple residue is dissolved in MeCN (2.5 mL) and stored at –18 °C.

The reaction was also done with HCl (2 Molar solution in Et_2O, 20 µl, 2.1 eq) for this reaction **7** (10 mg, 20 µmol, 1eq.) was dissolved in MeCN. The reaction mixture was stirred for a period of 5 min. Then all volatiles are removed *in vacuo* and the residue is washed with Et_2O (2x3 mL). The blue purple residue was used directly for UV-Vis measurements.

UV-Vis (MeCN, c = $4.794\cdot10^{-5}$ mol L^{-1}): λ_{max} (ε, 10^3 L mol^{-1} cm^{-1}) 609 (5.6), 561 (4.1), 521 (1.9), 432 (2.0), 407 (1.7), 302 (75.2), 265 (17.2), 253 (16.1), 226 (31.6), 205 (31.8) nm.

6,13-Bis(*N*,*N'*-dimethylethyleneguanidinyl)pentacene bis(hexafluoroantimonate), 8(SbF$_6$)$_2$

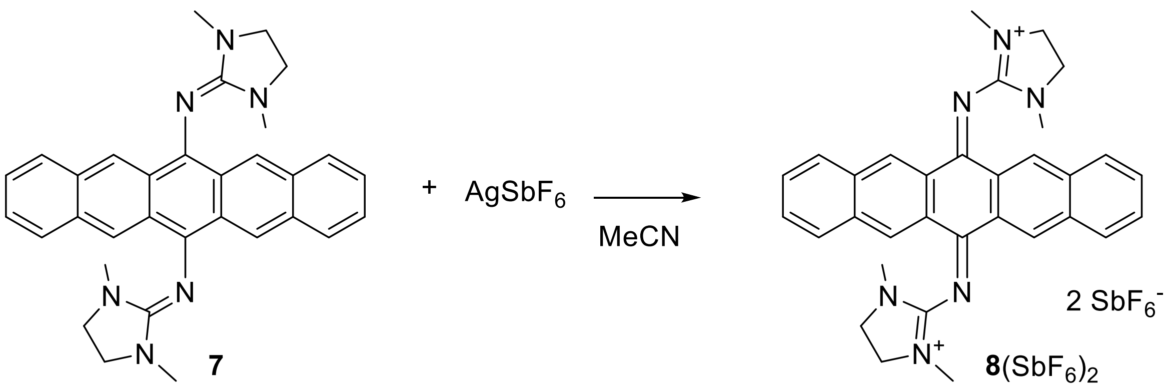

In a Schlenk tube, **7** (10 mg, 20 µmol, 1 eq.) and $AgSbF_6$ (14 mg, 40 µmol, 2.05 eq.) are mixed and MeCN (4 mL) is added under exclusion of light, resulting in an immediate colour change to a yellow solution. After stirring for 2 h, the reaction mixture is filtered, and the solvent removed *in vacuo*. The yellow residue is dissolved in MeCN (0.5 mL) and crystals are obtained by diffusion of Et_2O (5 mL) from the gas-phase into this solution (8 mg, 8 µmol, 41%).

^{1}H NMR (600.13 MHz, CD_3CN, 293 K): δ = 8.67 (s, 4 H, $CH_{arom.}$), 8.31-8.29 (m, 4 H, $CH_{arom.}$), 7.91-7.89 (m, 4 H, $CH_{arom.}$), 4.03 (s, 8 H, $CH_{2,gua.}$), 2.91 (s, 12 H, $CH_{3,gua.}$) ppm.

^{13}C{^{1}H} NMR (150.90 MHz, CD_3CN, 295 K): δ = 167.41 ($Cq_{arom.}$), 163.45 ($Cq_{gua.}$), 133.76 ($Cq_{arom.}$), 132.15 ($CH_{arom.}$), 131.50 ($CH_{arom.}$), 131.15 ($CH_{arom.}$), 128.63 ($Cq_{arom.}$), 49.85 ($CH_{2,gua.}$), 33.43 ($CH_{3,gua.}$) ppm.

IR (KBr): $\tilde{\nu}$ = 3031 (w), 2964 (w), 2943 (w), 2900 (w), 2817 (sh), 1685 (s), 1673 (s), 1617 (s), 1587 (s), 1546 (m), 1496 (w), 1481 (w), 1458 (m), 1466 (m), 1425 (w), 1410 (w), 1395 (m), 1375 (m), 1295 (s), 1285 (sh), 1206 (m), 1064 (m), 995 (m), 969 (m), 934 (m), 841 (w), 798 (w), 765 (m), 700 (w), 657 (vs), 627 (w), 473 (w) cm^{-1}.

UV-Vis (MeCN, c = $7.731\cdot10^{-5}$ mol L^{-1}): λ_{max} (ε, 10^3 L mol^{-1} cm^{-1}) = 419 (9.1), 312 (15.6), 224 (10.5), 214 (7.8) nm.

HR-MS (ESI^+, MeCN): m/z calcd for $C_{32}H_{32}N_6SbF_6^+$ $[M{-}SbF_6]^+$ = 735.1625, found m/z = 735.1620; m/z calcd for $C_{32}H_{32}N_6^{2+}$ $[M{-}2SbF_6]^{2+}$ = 250.1339, found m/z = 250.1340.

Elemental Analysis ($C_{32}H_{32}N_6Sb_2F_{12}$, *MW*: 972.15 g/mol): calcd. C 39.54, H 3.32, N 8.64; found C 39.11, H 3.53, N 8.86.

3. NMR analysis of the DA products

The DA products were investigated by ^{1}H, ^{13}C NMR as well as 2D-NMR and ^{1}H NOESY experiments to elucidate their structure in solution (see SI). The ^{1}H NMR (400 MHz, d_3-MeCN) spectrum of the butadiene adduct **2**$(BF_4)_2$ contains four singlet signals at δ = 3.97 (4 H), 3.78 (8 H), 3.04 (12 H) and 1.71 (6 H) ppm. The first two downfield shifted signals are broadened and belong to the methylene bridge and guanidinyl ethylene backbone. Structural conformation is further confirmed by the ^{1}H NOESY NMR showing correlation through space for the four guanidinyl CH_3 groups with the CH_2 ring atoms as well as with the guanidinyl ethylene backbone. Furthermore, the methyl groups attached to the six-membered ring show correlation through space with the CH_2 ring atoms, too. The signal broadening is caused by either rotation or inversion of the guanidinyl groups in solution which cannot be resolved on the NMR time scale, leading to similar chemical shifts for all CH_3 and CH_2 protons in the ^{1}H NMR spectra.

The ^{13}C NMR spectrum of **3**$(BF_4)_2$ contains eleven signals from which nine arise from carbon atoms connected to protons. These carbons bound directly to protons are assigned with the aid of ^{1}H-^{13}C HSQC spectra (see SI). All carbon atoms in the former cyclopentadiene moiety are chemically inequivalent, while for the guanidinyl moieties some carbon atoms are equivalent on the NMR time scale. For example, there are two quaternary carbon signals for the central guanidinyl carbons but only two instead of four signals for the connected methyl and the ethylene groups. The compound should therefore exhibit C_1 symmetry in solution and the urea azine moiety on top of the cyclopentene ring adopts a trans conformation as seen in the crystal structure. Chemical equivalence for the guanidinyl backbone (methyl groups and ethylene bridge) could be established through rotation about the CN azine-guanidine bond. Flipping inversion at the azine N atoms of the guanidinyl groups can probably be ruled out due to the inequivalence of the carbon signals of the two guanidinyl groups.

The ^{1}H NMR spectrum of the pentacene-DA product **5**$(BF_4)_2$ shows four signals for the twelve aromatic protons, one signal for the two CH-bridge atoms with a characteristic shift of 6.41 ppm and two signals for the four guanidinyl methyl groups, as well as two complex multiplets for the ethylene bridge of the guanidinyl moiety. The compound presumably exhibits C_2 symmetry in solution giving a structure in which the guanidinyl groups again adopt trans-conformation (as in the crystal structure), for which half of the atoms are chemically equivalent. Interestingly, from ^{1}H NOESY NMR we find direct chemical exchange for the guanidinyl protons in which the methyl groups interconvert. Similar spectra are obtained for the tetracene adduct.

4. Room-temperature reversible hetero Diels-Alder reaction of 6(BF_4)$_2$:

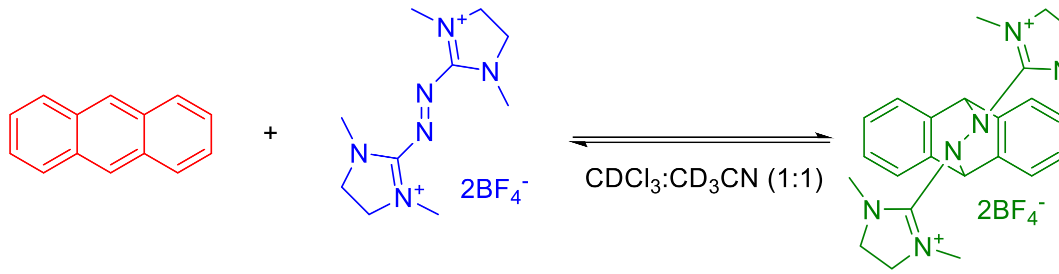

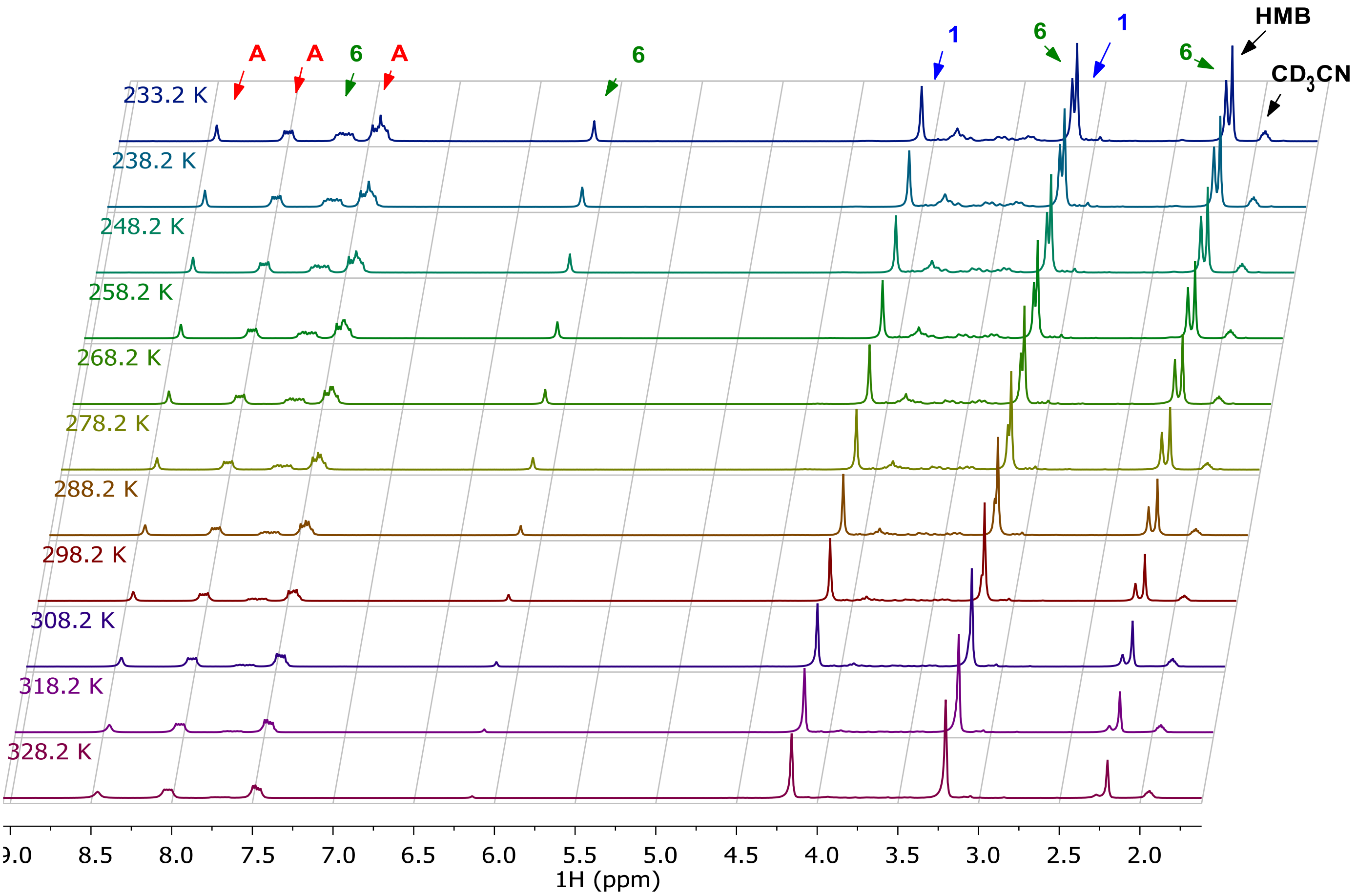

Figure 4.1. ^{1}H NMR spectra (199.87 MHz, $CDCl_3$:CD_3CN (1:1)) of the VT-NMR (temperature region –40 – 55 °C) experiment showing the reversible nature of the DA reaction with anthracene (**A**) and **1**(BF_4)$_2$ (with hexamethylbenzene (**HMB**) added as internal standard). At low temperatures, the equilibrium is on the side of the DA product **6**(BF_4)$_2$, and at higher temperatures it shifts towards the reactants, **1**(BF_4)$_2$ and anthracene. Note that only the major signals used for the integration are shown.

NMR Signals for the anthracene adduct (not isolated):

^{1}H NMR (199.87 MHz, CD_3CN:$CDCl_3$ 1:1, 298 K): δ = 7.74-7.63 (m, 4 H, $CH_{arom.}$), 7.49-7.44 (m, 4 H, $CH_{arom.,}$ Note: signals are covered by anthracene), 6.13 (s, 2 H, $CH_{DA\text{-}bridge}$), 3.97-3.41(m, 8 H, $CH_{2,gua.}$), 3.20 (s, 6 H, $CH_{3,gua.}$), 2.24 (s, 6 H, $CH_{3,gua.}$) ppm.

Estimation of the equilibrium constants (see Figure 4.1 and Table 4.1) relied on the law of mass conservation. Absolute concentrations in the equilibrium were estimated with the aid of the internal standard hexamethylbenzene (HMB c = 8.14 mM, **1** and anthracene at a concentration of 30 mM) by integration of respective peak signals for anthracene (A), the dienophile **1** and the Diels-Alder product **6**. In order to obtain more accurate concentrations, integration and speciation was carried out with the MestreNova internal module Mnova qNMR.[3] The peak areas were deconvoluted in cases where they overlapped with other signals (accuracy of integration ±5%, errors estimated by error convolution).

Table 4.1. Estimated equilibrium constants from the VT ^{1}H NMR experiment.

T / °C	K_{eq} [l/mol]	ΔK_{eq} [l/mol]	$\ln(K_{eq})$	$\Delta\ln(K_{eq})$
-40	79.92	9.78	4.3810	0.1224
-35	75.98	9.30	4.3305	0.1224
-25	68.50	8.38	4.2269	0.1224
-15	57.16	7.00	4.0459	0.1224
-5	47.86	5.86	3.8684	0.1224
+5	37.12	4.51	3.6142	0.1224
+15	26.40	3.23	3.2734	0.1224
+25	15.26	1.87	2.7249	0.1224
+35	10.37	1.27	2.3384	0.1224
+45	5.21	0.64	1.6514	0.1224
+55	2.73	0.33	1.0045	0.1224

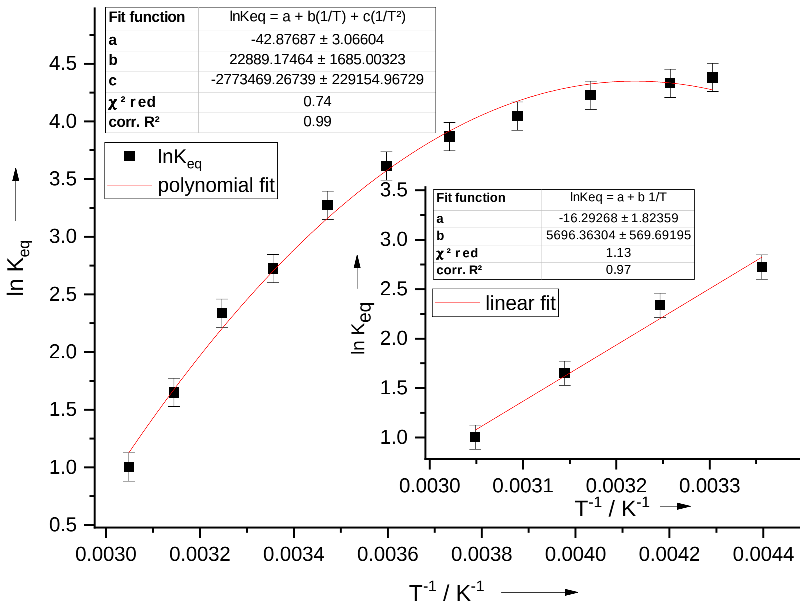

Figure 4.2. Van't Hoff plot ($\ln(K_{eq})$ versus T^{-1}) for the room-temperature reversible DA reaction between anthracene and **1**. Estimation of thermodynamic data was carried out for the full temperature region (–40-55 °C) with a polynomial fit assuming a non-linear Van't Hoff temperature dependence. For the higher temperature region (25-55 °C) shown in the inset, a linear fit was used. Results of the polynomial fit: ΔH(298K) = –35.6 ± 19.0 kJ mol^{-1}, ΔS(298K) = –97.1 ± 33.3 J mol^{-1} K^{-1}. Results of the linear fit: ΔH = –47 ± 5 kJ mol^{-1}, ΔS = –136 ± 15 J mol^{-1} K^{-1}.

The different solubility of the diene and dienophile component used in our reactions might hamper the analysis over a wide temperature regime. Anthracene is only partially soluble in MeCN in concentrations required for an NMR experiment,[4] while the dicationic dienophile is completely dissolved. In less polar solvents the opposite behaviour is found. For estimation of the equilibrium constant we therefore used a mixture of CD_3CN:$CDCl_3$ (1:1) to which an internal standard (hexamethylbenzene, 8.14 mM) was added. Evaluation of the thermodynamic parameters relied on a Van´t Hoff plot ($\ln(K_{eq})$ vs. $1/T$). For the full temperature region from –40 to +55 °C, a linear fit proved to be inadequate. The Van´t Hoff plot is linear on the assumption that the enthalpy and entropy are temperature-independent in the analysed temperature region. However, it is known that in some cases ΔH and ΔS vary significantly with temperature.[5] Deviations from the linear behaviour could be caused, for example, by a temperature-dependent conformational change or secondary equilibria

such as solubility.[5c] Analytical expressions for the temperature-dependence are usually based on the change of heat capacity during a reaction. Then, a polynomial fit is used to account for a non-constant reaction enthalpy (see Figure 4.2).[5b]
Fitting our NMR data with the quadratic function $\ln K_{eq} = a + b\frac{1}{T} + c\frac{1}{T^2}$ results in a standard reaction enthalpy $\Delta H^0 =$ –35.6 ± 19.0 J mol^{-1} K^{-1} and a standard reaction entropy $\Delta S^0 =$ –97.1 ± 33.3 J mol^{-1} K^{-1}.

Alternatively, the thermodynamic data could be derived from a linear fit for the higher temperature region (+25 to +55 °C), yielding a reaction enthalpy ΔH = –47 ± 5 kJ mol^{-1} and a reaction entropy ΔS = –135 ± 15 J mol^{-1} K^{-1}.

5. Selected structural parameters of the DA products 2-5

Table 5.1. Selected structural parameters (bond lengths in Å, angles in °, *α* dihedral angle between the two guanidinyl planes, *β* dihedral angle between the planes of the two carbon sites of the diene in the DA compound) of the DA products as well as $\mathbf{1_{red}}$ and **1**$(BF_4)_2$. Averaged values are given in cases with two independent molecules in the unit cell or two bonds of the same type.

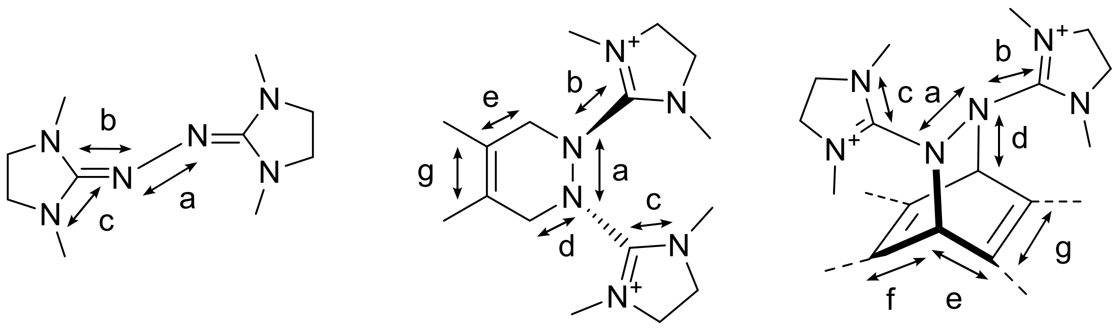

Compound	a	b	c	d	e	f	g	α	β
$\mathbf{1_{red}}$	1.416(1)	1.296(1)	1.396(1)	-	-	-	-	-	-
**1**$(BF_4)_2$	1.259(3)	1.399(2)	1.333(2)	-	-	-	-	-	-
**2**$(BF_4)_2$	1.405(4)	1.365(3)	1.330(4)	1.470(3)	1.500(4)	-	-	81	-
**3**$(BF_4)_2$	1.438(5)	1.391(6)	1.326(6)	1.554(6)	1.508(6)	1.514(6)<br>1.538(6)	1.326(3)	73	129
**4**$(BF_4)_2$	1.447(2)	1.390(2)	1.325(2)	1.528(2)	1.504(2)	1.512(2)	1.401(2)<br>1.426(2)	76	128
**5**$(BF_4)_2$	1.449(2)	1.391(2)	1.322(3)	1.525(2)	1.501(3)	1.513(3)	1.425(3)	82	125

6. Cyclovoltammetric studies

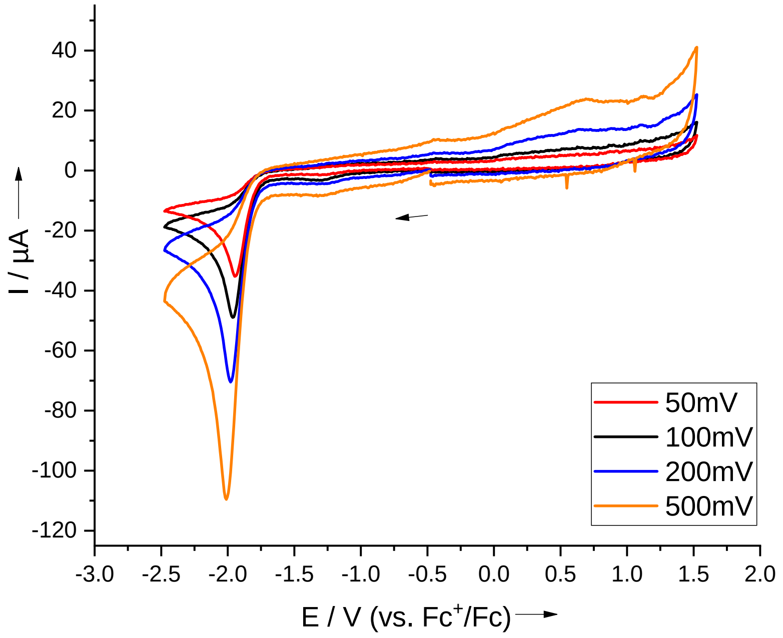

Figure 6.1. Cyclic voltammograms recorded for the DA product **2**$(BF_4)_2$ in MeCN at variable scan speed in reductive direction (0.1 M, N(*n*Bu)$_4$PF$_6$, Ag/AgCl reference electrode). Potentials given vs. Fc^+/Fc (applied as external reference).

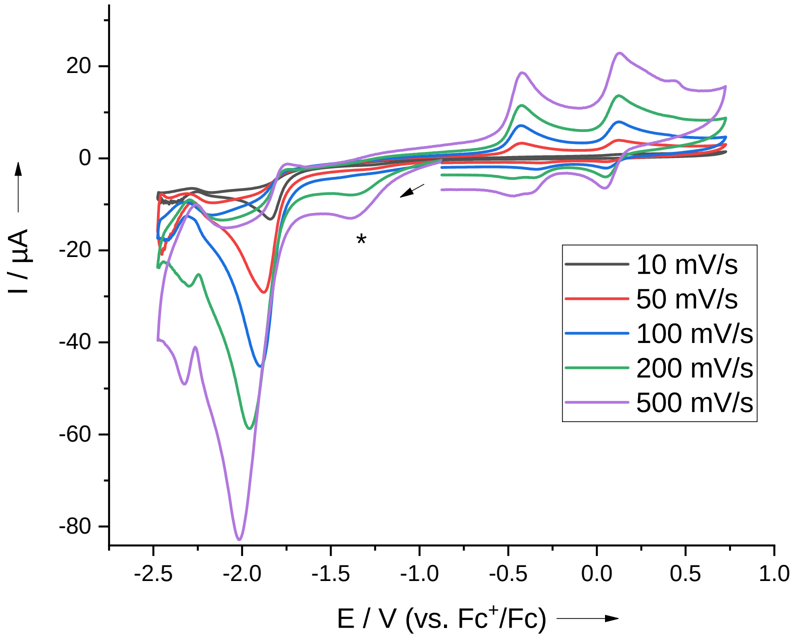

Figure 6.2. Cyclic voltammograms recorded for the DA product **5**$(BF_4)_2$ in MeCN at variable scan speed in reductive direction (0.1 M, N(*n*Bu)$_4$PF$_6$, Ag/AgCl reference electrode). Potentials given vs. Fc^+/Fc (applied as external reference). The wave marked by an asterisk is due to O_2 reduction.

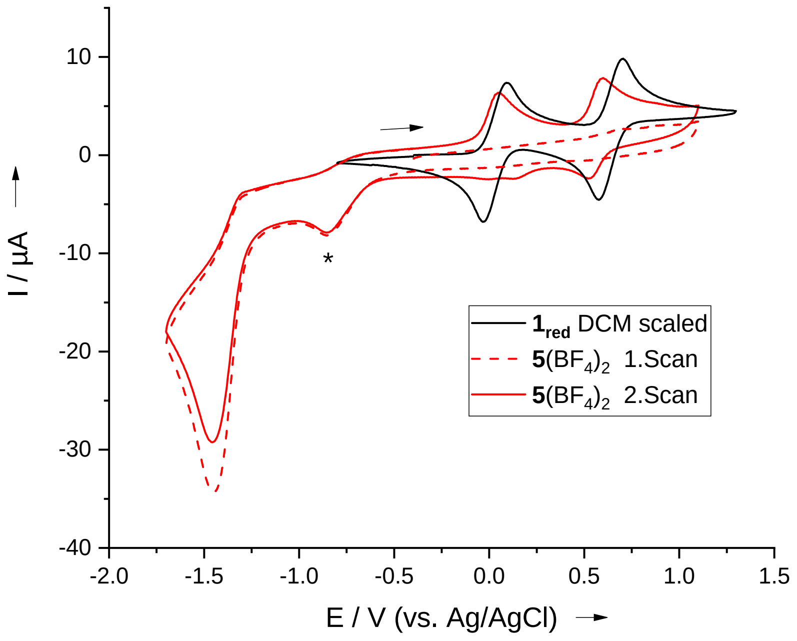

Figure 6.3. Comparison between the cyclic voltammograms recorded for the DA product **5**$(BF_4)_2$ in MeCN in oxidative direction (two consecutive scans) and neutral $\mathbf{1}_{\mathbf{red}}$ in DCM (0.1 M N(*n*Bu)$_4$PF$_6$, Ag/AgCl reference electrode, measured at scan rates of 100 mV s^{-1}. The wave marked by an asterisk is due to O_2 reduction.

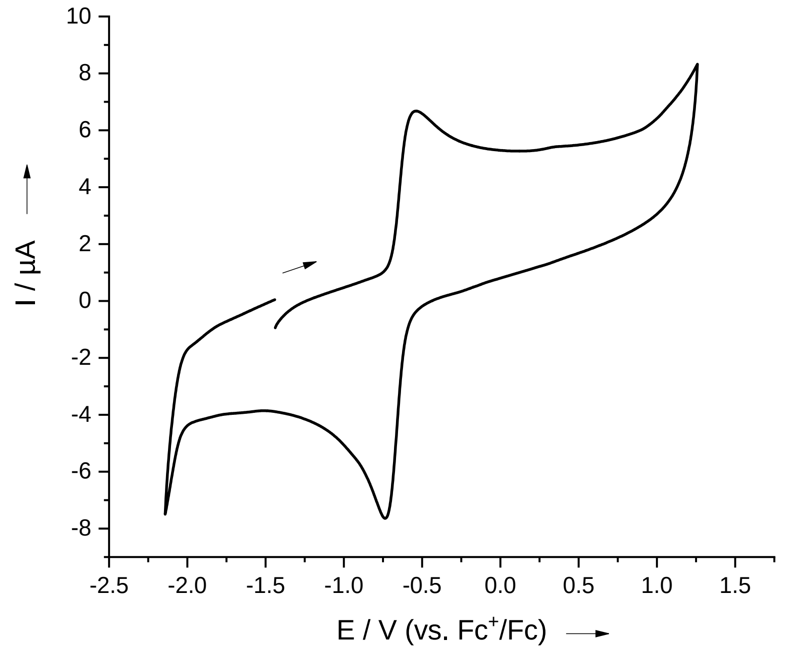

Figure 6.4. Cyclic voltammogram of **7** in DCM (0.1 M N(*n*Bu)$_4$PF$_6$, scan speed 100 mV/s, Ag/AgCl reference electrode). Potentials given vs. Fc^+/Fc (applied as external reference).

The electronic properties of compound **7** were studied by cyclic voltammetry in DCM (Figure 6.4). The voltammogram shows a single, quasi-reversible redox process at $E_{1/2}$ = –0.65 V (E_{Ox} = –0.56 V and E_{red} = –0.73 V). From chemical oxidation of **7** with $AgSbF_6$ (1 eq.) in MeCN we were able to isolate yellow crystals showing a stoichiometry of two SbF_6^- anions per one bisguanidinyl-pentacene.

Therefore, we assume that the wave observed in the cyclic voltammogram belongs to a quasi-reversible two-electron redox process. The quasi-reversible nature indicates that there should be only a small structural change between the neutral and dicationic redox state.[6] This is in accordance with the DFT calculations showing only small changes for the critical parameters between the calculated structure of **7** and crystal structure of **8**. No formation of a butterfly conformation was observed upon oxidation, and the acene moiety is only slightly twisted. A related compound, 1,4-bis-tetramethylguanidinyl-benzene, was investigated earlier by our group.[7] The CV spectra show one quasi-reversible two-electron redox process ($E_{1/2}$ = –0.18 V vs. Fc/Fc^+ in DCM), ΔE = 180 mV). The lower redox potential of **7** is in accordance with the more electron-rich acene bridge.

7. Titration of 7 with $AgSbF_6$

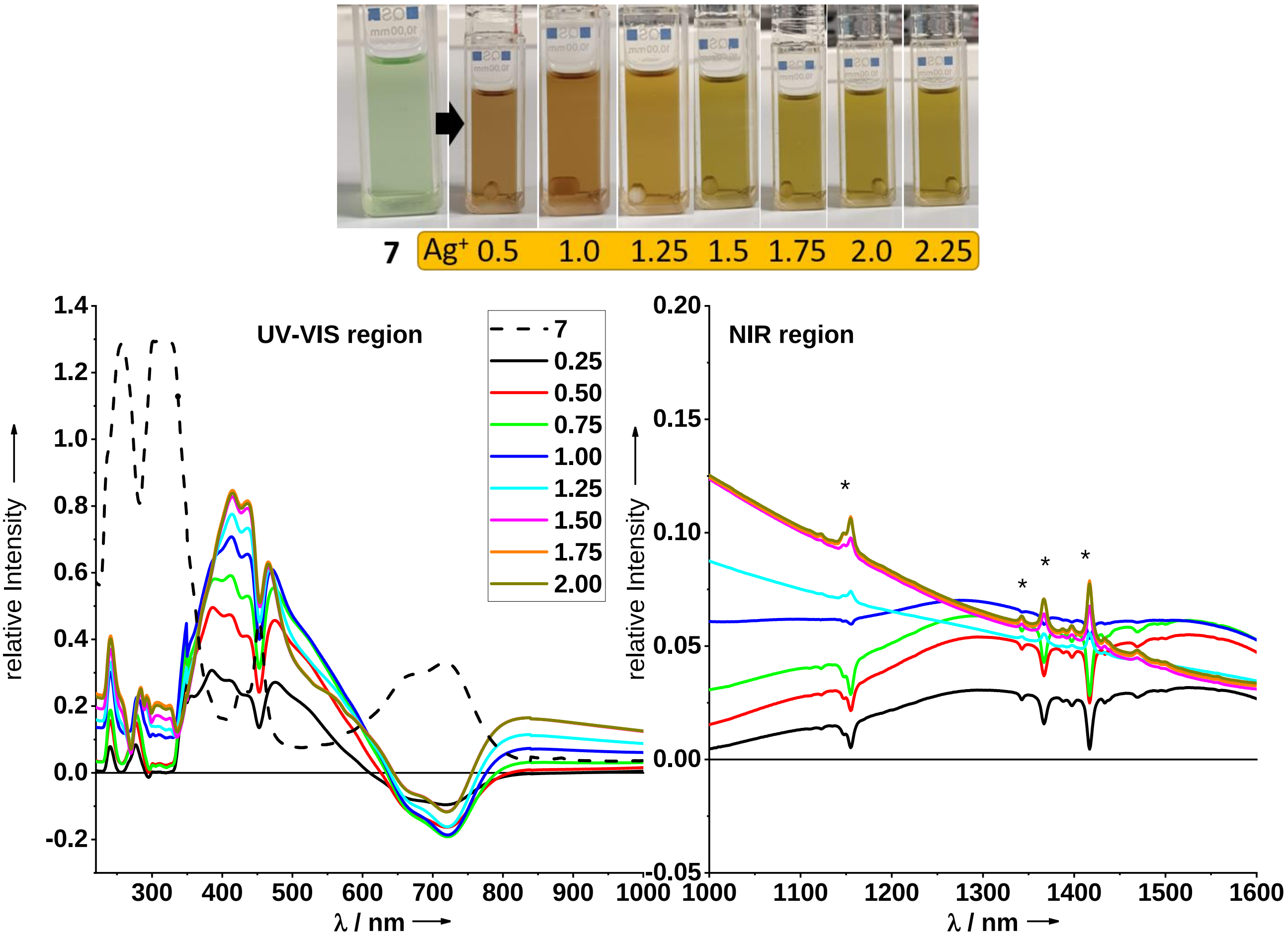

Figure 7.1. UV-Vis-NIR spectra recorded for the titration of **7** with $AgSbF_6$ (applied equivalents are given in the legend) in DCM. The spectrum of the solvent plus compound **7** was used as reference. The spectrum of compound **7** in DCM referenced to the solvent is included for comparison (dashed line). The asterisks highlight the bands due to the DCM stabilizer amylene. The photos illustrate the colour changes during the titration. The spectra point to oxidation in two one-electron steps, with intermediate formation of the open shell compound **7**$^{\bullet+}$.

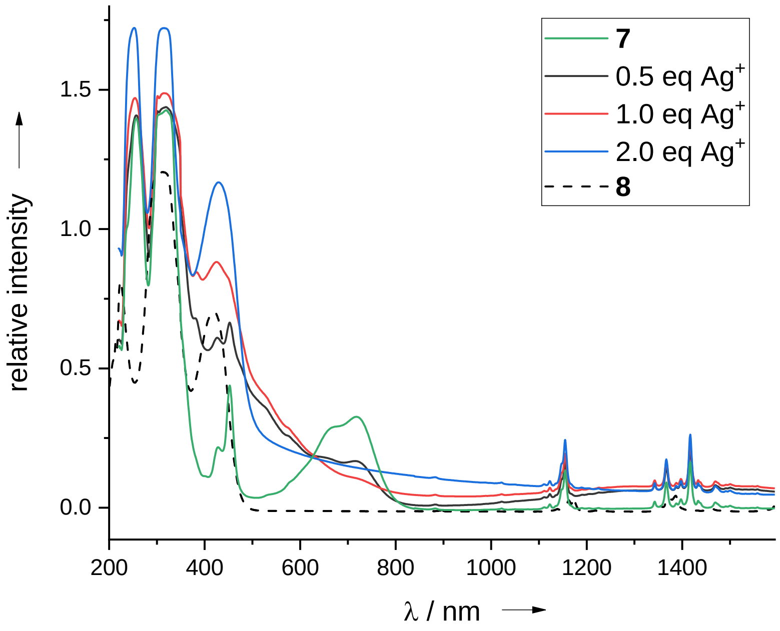

Figure 7.2. UV-Vis-NIR spectra recorded for the titration of **7** with $AgSbF_6$ (equivalents are given in the legend) in DCM. The solvent spectrum is used as reference. The spectrum of **8** in MeCN is included for comparison (dashed line). Compound **8** was synthesised by oxidation with $AgSbF_6$ (2 eq.) and purified by crystallisation.

UV-Vis spectra recorded upon titration of **7** with $AgSbF_6$ in DCM indicate a two-step oxidation process (see Figure 7.1 and 7.2). Upon oxidation with 0.25 to 1.00 eq. of $AgSbF_6$ the *p*-band (718 nm) as well as the higher energy *α*-band (453 nm) of the neutral from vanished. Instead, several new bands in the region 350-650 nm and weak bands in the NIR region between 1250 and 1600 nm appeared. The colour of the solution changes from green to red-brown. Upon further oxidation with up to 2 eq. $AgSbF_6$, the shoulder at about 525 nm as well as the NIR bands disappeared in the UV-Vis spectra (in comparison with the spectra for 1.00 eq. $AgSbF_6$). The colour of the solution now changed from red to yellow. According to TD-DFT calculations for $\mathbf{7}^{\bullet+}$, the lowest energy transition is located in the NIR region at 1337 nm, and the second lowest energy transition is located at 544 nm (see also Figure 7.2). The very broad absorption in the NIR might be composed of more than one band. The lower-energy band is tentatively assigned to a charge resonance (CR) band, originating from the class III or borderline class III/II mixed-valence (MV) system.[8] (Alternatively, it is due to an enlarged π-system resulting from dimer formation of neutral **7** with its radical monocation $\mathbf{7}^{\bullet+}$.[9]) The second lowest energy transition is also in good accordance with the observed shoulder at 525 nm. All attempts to isolate a salt of the radical $\mathbf{7}^{\bullet+}$ failed, underlining its instability towards disproportionation to the dicationic and neutral redox states. Nevertheless, the results argue for a two-step oxidation process (two one-electron steps) as also found for amino-tetracene and amino-anthracene derivatives.[8c,10] Differential pulse voltammetry showed for amino-tetracene and amino-anthracene derivatives that the observed quasi-reversible single wave voltammogram consists of two successive reversible one-electron oxidation steps with a minuscule potential difference of ΔE = 40 mV and ΔE = 60 mV, respectively.

In relation to our work we want to note the work by Ito *et al.* on the synthesis and redox properties of various diamino-acenes.[6,10] In summary these systems represent Wurster-type redox systems characterised by three distinct redox states. TMPD, the precursor to the radical denoted Wurster's blue, is a well-known representative.[11] However as shown by Ito *et al.,* by enlarging the π-system of the bridge between the two redox sites, the two step oxidation process is not necessarily preserved, as structural differences in the oxidised form might induce a change to a single two-electron process (with inverted oxidation potentials) and also might affect the electrochemical reversibility.

8. EPR spectra

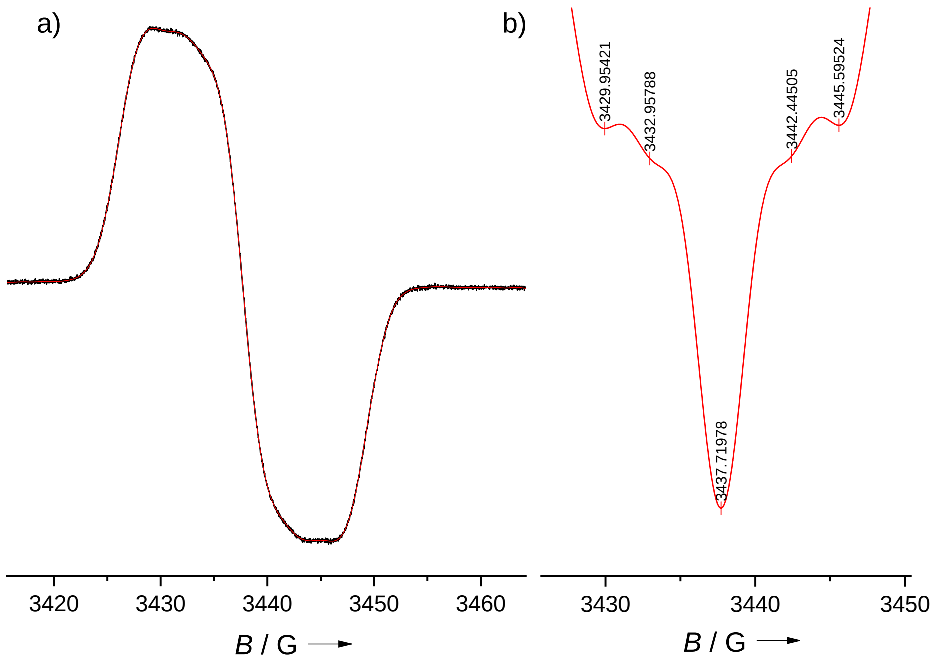

Figure 8.1. a) EPR spectrum (first derivative) of the reaction mixture of **7** and Ag(SbF_6) (0.5 eq.) in DCM. The spectrum was analysed with a low-pass filter (0.3Hz, red line). b) Second derivative of the EPR signal. Spectrometer frequency *f* = 9.637092 GHz, g = 2.0029.

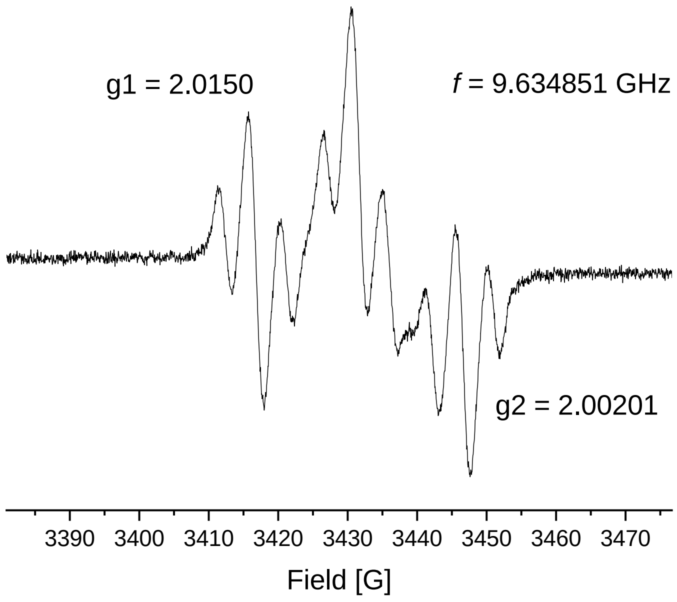

Figure 8.2. EPR spectrum of the filtrate of the reaction mixture of **5**(BF_4)$_2$ and LDA (1 eq.) in THF-d_8. Spectrometer frequency *f* = 9.634851 GHz. The spectrum probably shows two paramagnetic species.

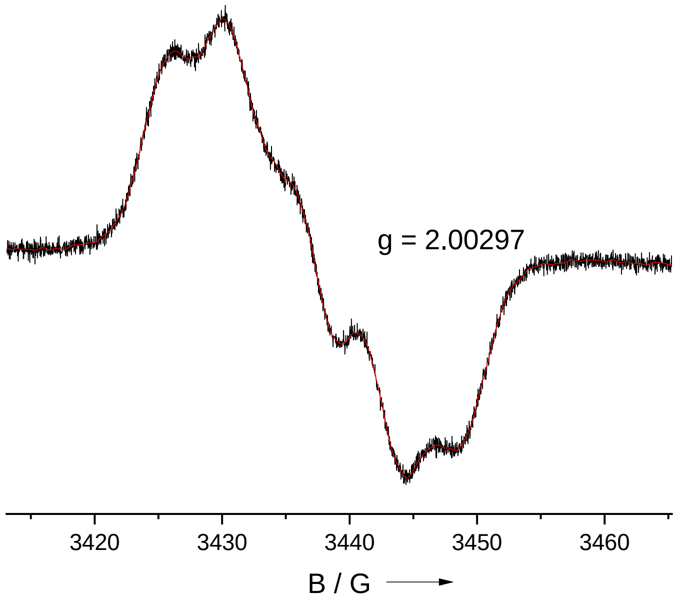

Figure 8.3. EPR spectrum of the filtrate of the reaction mixture of **5**$(BF_4)_2$ and LDA (1 eq.) in THF-d_8 after about 1 d. The spectrum was analysed with a low-pass filter (1 Hz, red line). Spectrometer frequency *f* = 9.636282 GHz.

Further evidence for the presence of a radical form $\mathbf{7}^{\bullet+}$ is provided by EPR experiments. The EPR spectrum of a solution containing **7** and 0.5 or 1.0 eq. of $AgSbF_6$ in DCM shows a broad paramagnetic signal (g = 2.0029) (see Figure 8.1). Hyperfine splitting is almost unresolved, but the free electron is expected to predominantly couple with two nitrogen cores adjacent to the pentacene moiety, therefore one would expect a quintet signal (not resolved). Interestingly investigation of the filtrate obtained from the reaction of the pentacene DA product **5** and LDA in THF shows a similar but better resolved paramagnetic signal (g = 2.0020) and a second signal (g = 2.0150) (Figure 8.2). Preparation of the EPR probe under air or measuring of the probe after about a day leads to vanishing of the second signal and broadening of the first signal (g = 2.0029) while the *hfs* is still visible (Figure 8.3). Observation of a paramagnetic signal for the reaction of **5**$(BF_4)_2$ with KOtBu/LDA to give **7** indicates oxidation to $\mathbf{7}^{\bullet+}$, probably due to a competing redox reaction with **1** accompanied by retro Diels-Alder reaction:

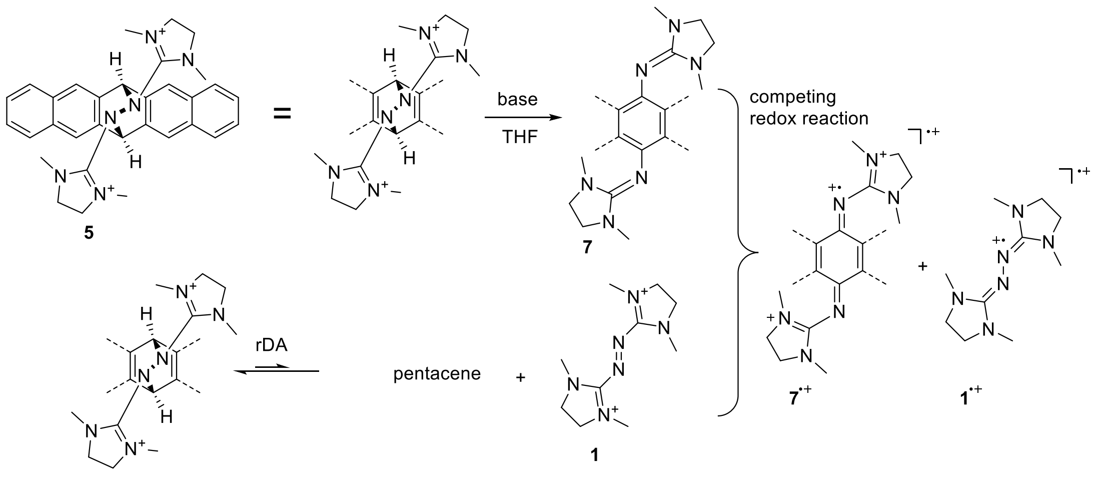

9. Computational data

Ionic Diels-Alder reactions are quite rare in chemistry. They compromise reactions with positively or negatively charged ionic species as diene or dienophile component.[12] The ionic nature is preserved along the reaction coordinate until formation of the ionic cycloadduct.[12e] In the last years several theoretical studies have been devoted to the mechanism of ionic DA reactions.[12d,e,13] In these studies, different mechanisms have been proposed for DA reactions of non-charged and charged species. By inspection of the transition state structures, correlations between the experimental reaction rates and the global charge transfer were disclosed.[12d] Recent work by Domingo *et al.* on the basis of favourable nucleophile/electrophilic interactions in the TS show that the rates of DA reactions (from neutral via polar to ionic DA) increases with polarity.[12d] Therefore, ionic DA reactions proceed fast even at low temperatures. In a few cases the solvent effect on the transition states was included, showing significant influence on the energy of activation as well as regioselectivity predictions.[12d,13b]

In our case the solvent effect is significant due to the charge of +2 of the dienophile. Therefore, some preliminary investigations were carried out on the influence of the solvent on the thermodynamics as well as kinetics of the herein described ionic DA reactions. Diene, dienophile and DA product were optimised at the B3LYP+D3/def2-TZVP level of theory and the solvent effect was estimated by single-point calculations with the conductor-like screening model (COSMO) for the solvent acetonitrile (ε_r = 37.5). The calculated thermodynamic data, collected in Table 9.1, indeed highlight the solvent effect on the reaction thermodynamics. Overall, they are less exothermic and less exergonic for ε_r = 37.5 than for ε_r = 1. These results are in line with the results of the studies of Domingo *et al.* on DA reactions with protonated imine derivatives and cyclopentadiene, showing that the separated reactants are preferentially stabilized due to larger localisation of the positive charge on the dienophile.[13b] The calculations also explain the experimental result that no DA reaction occurs with the dienes furan and pyrrole. Additionally, they highlight that for anthracene, for which a chemical equilibrium is found at room temperature, the position of the equilibrium depends on the relative solvent permittivity (the solvent polarity).

Table 9.1. Calculated ΔG and ΔH values for the DA reactions (values given in kJ mol^{-1}), with and without inclusion of the solvent effect (COSMO, ε_r = 37.5 for MeCN). For the butadiene reaction the values were also calculated with optimised structures including the BF_4^- anions in the dienophile and DA product.

	B3LYP+D3/def2-TZVP +COSMO (sp)		B3LYP+D3/def2-TZVP	
Diene	ΔG	ΔH	ΔG	ΔH
E-2,3-dimethylbutadiene	-75.4	-143.5	-112.4	-180.5
including 2 BF_4^-	-72.5	-140.6	-78.8	-147.0
Z-2,3-dimethylbutadiene	-82.5	-152.1	-119.7	-189.3
including 2 BF_4^-	-79.6	-149.3	-86.1	-155.8
furane	68.6	-3.8	42.3	-30.1
pyrrol	114.5	40.1	70.2	-4.2
cyclopentadiene	-1	-75.6	-34.6	-109.3
anthracene	30.7	-45	-49.9	-125.6
tetracene	4.5	-71.1	-88.4	-164.0
pentacene	-24.2	-99.7	-128.4	-203.9

Qualitative information about the reaction kinetics could be derived from the Frontier Molecular Orbital (FMO) theory. According to the FMO theory (considering only electronic interactions between HOMO/LUMO in the rate-determining TS), the reaction rate of the DA reaction is proportional to the interaction energy of the HOMO and LUMO orbitals of diene and dienophile.[14] The interaction energy depends on the degree of favourable orbital overlap as well as the energy difference between the frontier orbitals.[15] For a normal electron demand DA reaction the HOMO of the nucleophilic diene and the LUMO of the electrophilic dienophile are of relevance.

Assuming that the orbital overlap for a given series of reactions is similar, the interaction energy only depends on the HOMO-LUMO gap. Hence, we compared the HOMO-LUMO gap between the dienophile **1** and the corresponding dienes with and without solvent effect (COSMO), and in addition tested the influence of an inclusion of the BF_4^- anions for the DA reaction with 2,3-dimethylebutadiene (Figure 9.1). As expected, more electron rich dienes have higher HOMO energies, and the inclusion of the solvent effect for the neutral dienes only slightly alters the frontier orbital energies. On the other hand, the inclusion of the solvent effect raises the energies of both HOMO and LUMO of the doubly-charged dienophile significantly. The LUMO energy of the diene is now in the region where interaction with the diene HOMO is expected for the case of a normal electron-demand DA reaction. Interestingly, inclusion of the anions for the dienophile in the DFT calculations has a similar effect on the energy of the frontier MOs. Inclusion of the solvent effect for these salts again raises the HOMO and LUMO energies, but only slightly. Therefore, the COSMO structure of **1** can be considered as a good approximation (counterions/salts in frequency calculation are often problematic since they often have multiple minima structures on the PES). Qualitatively, the reaction rate is larger for cyclopentadiene than for butadiene. This experimental result could be explained within the framework of the FMO theory by the smaller HOMO-LUMO gap (diene/dienophile), in line with our basic theoretical analysis. Furthermore, one would expect the rate to increase with the size of the acene. We can say that for the best-soluble acene in this series, anthracene, the rate is high as the equilibrium quickly (<5 min) responds to temperature changes. However, one would expect the reactions with the larger acenes to be even faster. Here the poor solubility might be the limiting factor.

Another point is the competition between DA pathway and an electron transfers (redox) pathway. As the dienes become more electron rich an electron transfer might be favoured. The competing redox pathway is also influenced by the solvent as the LUMO energy of the dienophile depends on the solvent polarity.

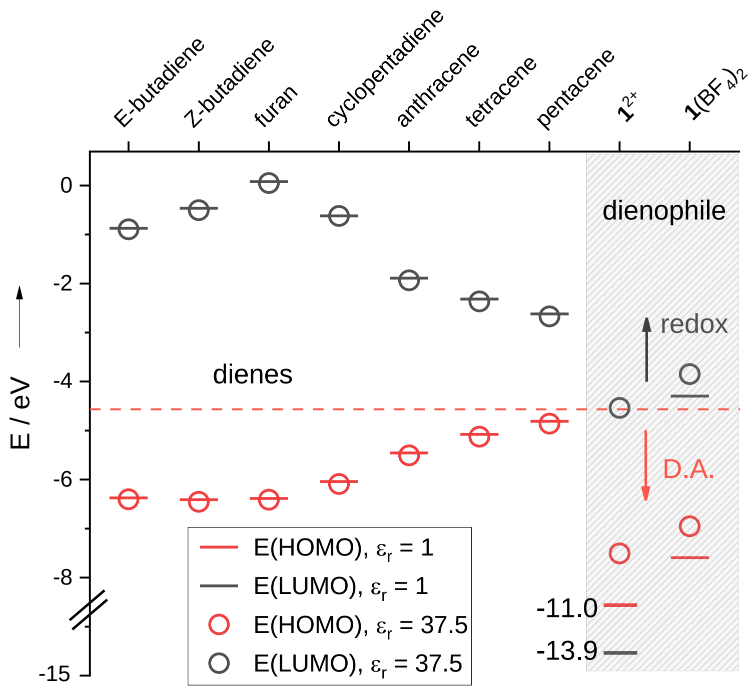

Figure 9.1. HOMO and LUMO energies (B3LYP+D3/def2-TZVP) for the applied dienophile and dienes with and without inclusion of the solvent effect (COMSO, ε_r = 37.5 for MeCN). The impact of the two BF_4^- anions on the orbital energies of the dienophile has also been considered (**1** vs. **1**$(BF_4)_2$). Note that butadiene stands for 2,3-dimethylebutadiene.

TD-DFT calculations, vertical transitions

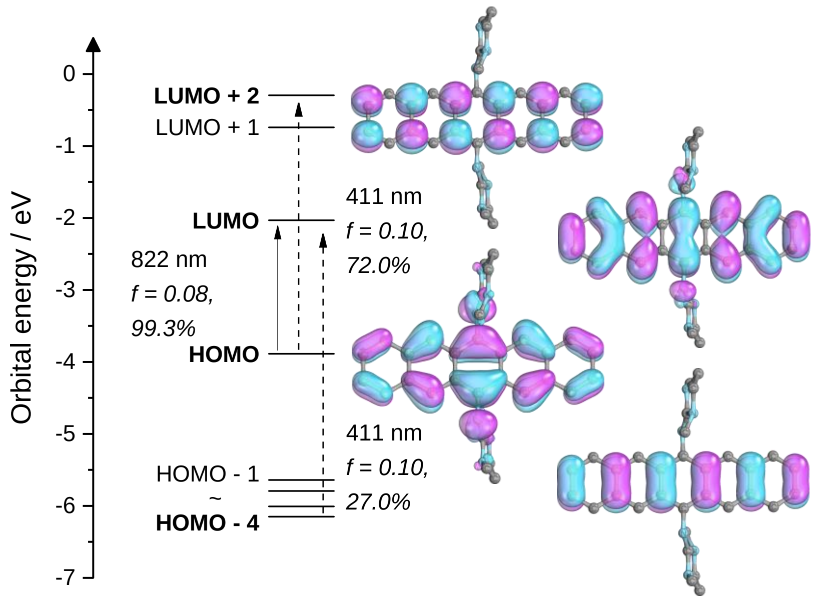

Figure 9.2. Frontier Kohn-Sham orbital energy levels for the ground state of **7** at the B3LYP+D3/TZVP level of theory. The solid and dashed arrows represent the major contributions related to the lowest and next lowest vertical transitions calculated by TD-DFT. Data gives the calculated vertical transition energy and oscillator strength as well as the orbital contributions (squared "coefficient" of a single electron excitation from orbital i to orbital a) of the related excited state. Orbitals plotted at 80 % threshold.

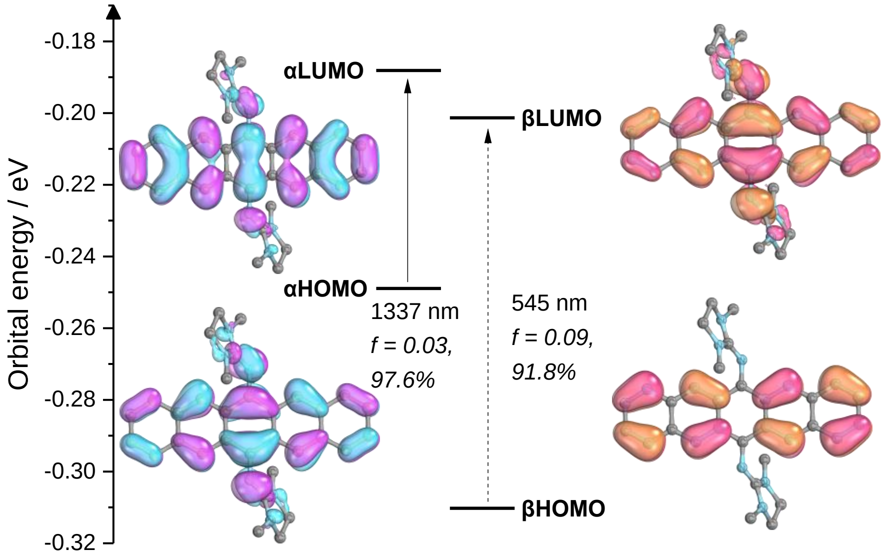

Figure 9.3. Frontier Kohn-Sham orbital energy levels for the ground state of $\mathbf{7}^{\bullet+}$ at the B3LYP+D3/TZVP level of theory.

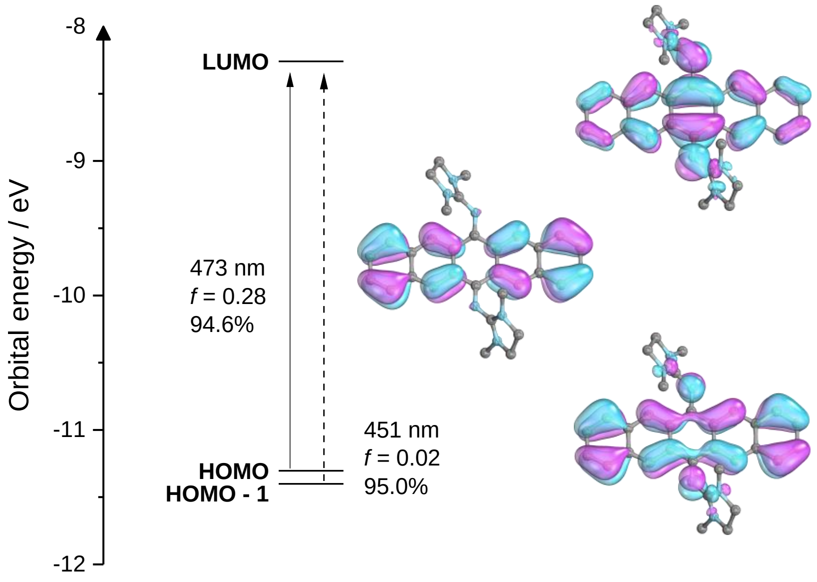

Figure 9.4. Frontier Kohn-Sham orbital energy levels for the ground state of **8** at the B3LYP+D3/TZVP level of theory.

10. Structural comparison of 7

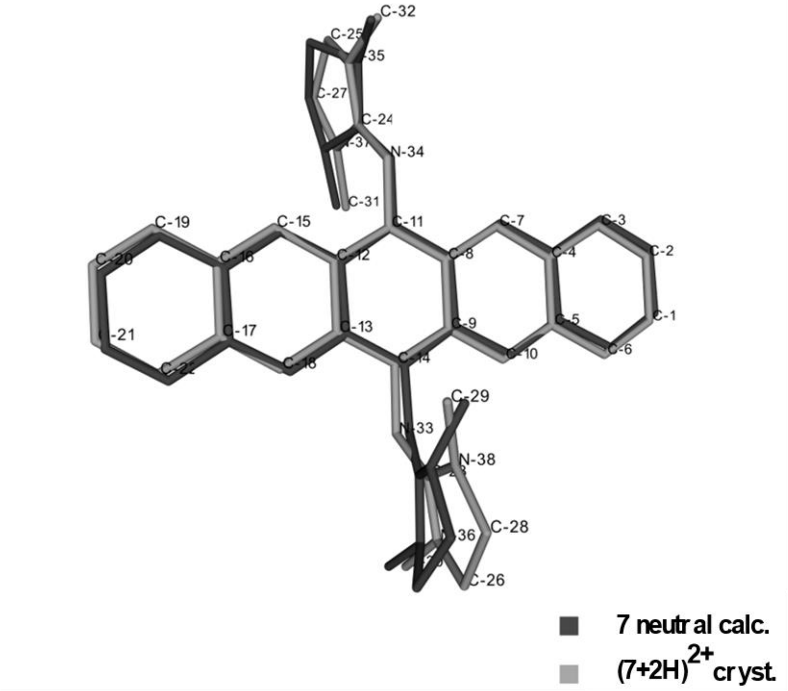

Figure 10.1. Comparison of the calculated structure of **7** (B3LYP+D3/TZVP) with the results obtained from XRD analysis for (**7**+2H)$^{2+}$, plotted with the program aRMSD.

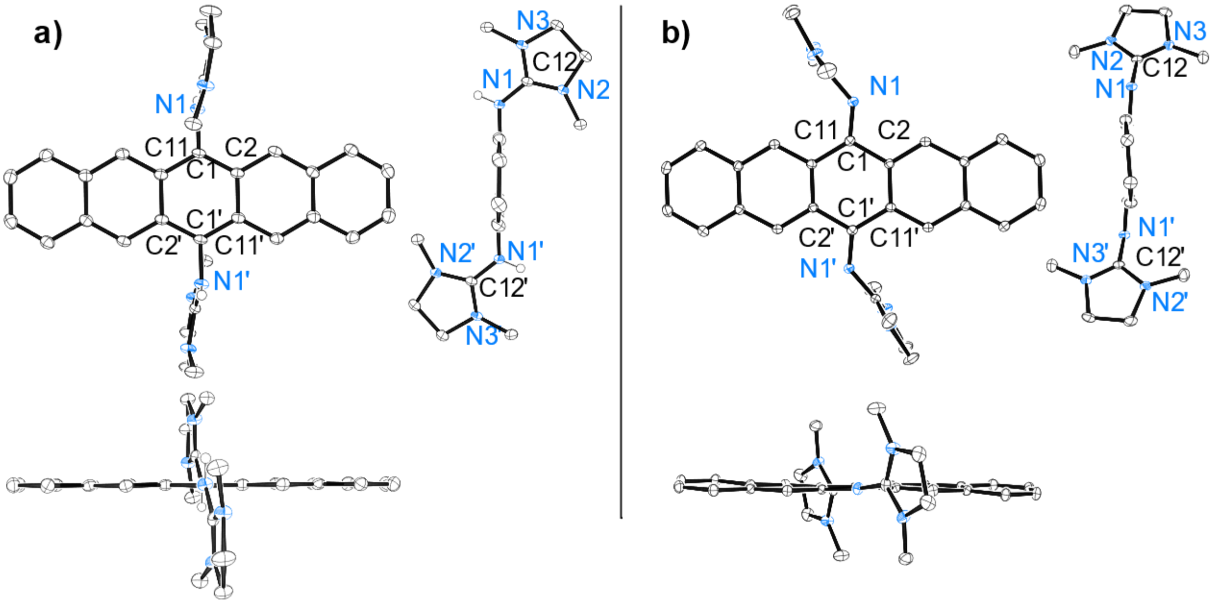

Figure 10.2. Molecular structures of (**7**+2H)$^{2+}$(OTf^{-})$_2$ (a) and **8**(SbF$_6$)$_2$ (b) (anions omitted). In case of (**7**+2H)$^{2+}$(OTf^{-})$_2$ there are two molecules of MeCN per unit. All carbon-bound hydrogens are omitted for clarity. Displacement ellipsoids drawn at the 50% probability level.

The structure of neutral **7** was calculated at B3LYP+D3/TZVP level of theory. The comparison between the calculated structure of **7** and that obtained by SCXRD for (**7**+2H)$^{2+}$ (see Figure 10.1) shows that both structures are similar, deviating only in some bond lengths. Interestingly in the protonated form the two guanidinyl groups are located in a plane almost perpendicular to the pentacene ring plane (see Figure 10.2a). In the oxidised form the guanidinyl groups are twisted about 90° (see Figure 10.2b). While we find a planar acene structure for the protonated form, the oxidised form shows a slightly twisted acene structure. Furthermore, the angle between the acene carbon and the guanidinyl group (∠(C11-C1-N1)) is larger than 120°.

These observations are in line with earlier studies by our group on the structural parameters of 1,4-bis(tetramethylguanidinyl)-benzene.[7] For the oxidised form we also found a bent-twisted structure, in which the nitrogen atom establishes π-interactions with the carbon π-system as well as the CN_2 group of the guanidinyl group.

11. Results of the crystal structure determinations

**Compound**	**2**(BF$_4$)$_2$	**3**(BF$_4$)$_2$	**4**(BF$_4$)$_2$	**5**(BF$_4$)$_2$
**CCDC No.**	1982181	1982183	1982184	1982182
Formula	$C_{32}H_{60}B_4F_{16}N_{12}$	$C_{16}H_{27.5}B_2F_8N_{6.5}$	$C_{56}H_{64}B_4F_{16}N_{12}$	$C_{32}H_{34}B_2F_8N_6$
$D_{calc.}$/ g cm^{-3}	1.436	1.475	1.411	1.431
μ/mm^{-1}	0.134	0.138	0.12	0.119
Formula Weight	960.16	484.56	1252.43	676.3
Colour	colourless	clear colourless	clear light yellow	yellow
Shape	box shaped	block	block	[shape ?]
Size/mm^3	0.50×0.30×0.20	0.20×0.14×0.08	0.58×0.23×0.20	0.44×0.33×0.18
T/K	120	100(2)	100(2)	120
Crystal System	monoclinic	monoclinic	monoclinic	monoclinic
Space Group	$P2_1/c$	$P2_1/n$	$P2_1/c$	$P2_1/n$
a/Å	24.350(5)	10.5539(19)	21.0879(8)	12.4813(8)
b/Å	13.873(3)	14.733(2)	17.9336(6)	18.0987(9)
c/Å	13.535(3)	28.133(5)	16.2002(6)	14.5247(9)
α/°	90	90	90	90
β/°	103.78(3)	93.736(6)	105.783(2)	106.935(2)
γ/°	90	90	90	90
V/Å^3	4440.5(16)	4365.2(13)	5895.6(4)	3138.8(3)
Z	4	8	4	4
Z'	1	2	1	1
Wavelength/Å	0.71073	0.71073	0.71073	0.71073
Radiation type	MoK_α	MoK_α	MoK_α	Mo K_α
Θ_{min}/°	0.861	2.377	1.814	2.21
Θ_{max}/°	28	27	28.5	28.78
Measured Refl's.	78799	60381	296594	50507
Ind't Refl's	10716	9516	14910	8152
Refl's with I > 2(I)	6149	5545	11590	5465
R_{int}	0.0949	0.1277	0.0605	0.0771
Parameters	589	631	886	456
Restraints	0	85	194	6
Largest Peak	0.73	1.506	0.715	0.71
Deepest Hole	-0.338	-0.509	-0.533	-0.7208
GooF	1.055	1.048	1.02	1.0373
wR_2 (all data)	0.2461	0.2687	0.1463	0.1785
wR_2	0.2187	0.2349	0.1303	0.1589
R_1 (all data)	0.1508	0.1565	0.0684	0.0979
R_1	0.0885	0.0953	0.0518	0.0643

**Compound**	($\mathbf{7}$+2H)$^{2+}$(OTf^{-})$_2$	**8**
**CCDC No.**	1982185	1982186
Formula	$C_{38}H_{40}F_6N_8O_6S_2$	$C_{32}H_{32}F_{12}N_6Sb_2$
$D_{calc.}$/ g cm^{-3}	1.473	1.842
μ/mm^{-1}	0.22	1.639
Formula Weight	882.9	972.13
Colour	clear light red	clear light yellow
Shape	block	cube
Size/mm^3	0.16×0.10×0.08	0.19×0.15×0.14
T/K	100	100
Crystal System	triclinic	monoclinic
Space Group	P-1	$P2_1/n$
a/Å	7.557(3)	9.3514(5)
b/Å	10.293(3)	12.1077(8)
c/Å	12.890(5)	15.4830(11)
α/°	85.709(14)	90
β/°	87.404(11)	90.690(3)
γ/°	84.859(8)	90
V/Å^3	995.1(6)	1752.92(19)
Z	1	2
Z'	0.5	0.5
Wavelength/Å	0.71073	0.71073
Radiation type	MoK$_\alpha$	MoK$_\alpha$
Θ_{min}/°	1.992	2.135
Θ_{max}/°	28.498	30
Measured Refl's.	45312	56105
Ind't Refl's	5043	5108
Refl's with I > 2(I)	4088	4471
R_{int}	0.0594	0.0575
Parameters	278	237
Restraints	0	0
Largest Peak	0.426	0.578
Deepest Hole	-0.44	-0.601
GooF	1.053	1.068
wR_2 (all data)	0.107	0.065
wR_2	0.0975	0.0618
R_1 (all data)	0.057	0.0313
R_1	0.044	0.0244

12. 1D- and (selected) 2D-NMR spectra of the described compounds

2(BF$_4$)$_2$

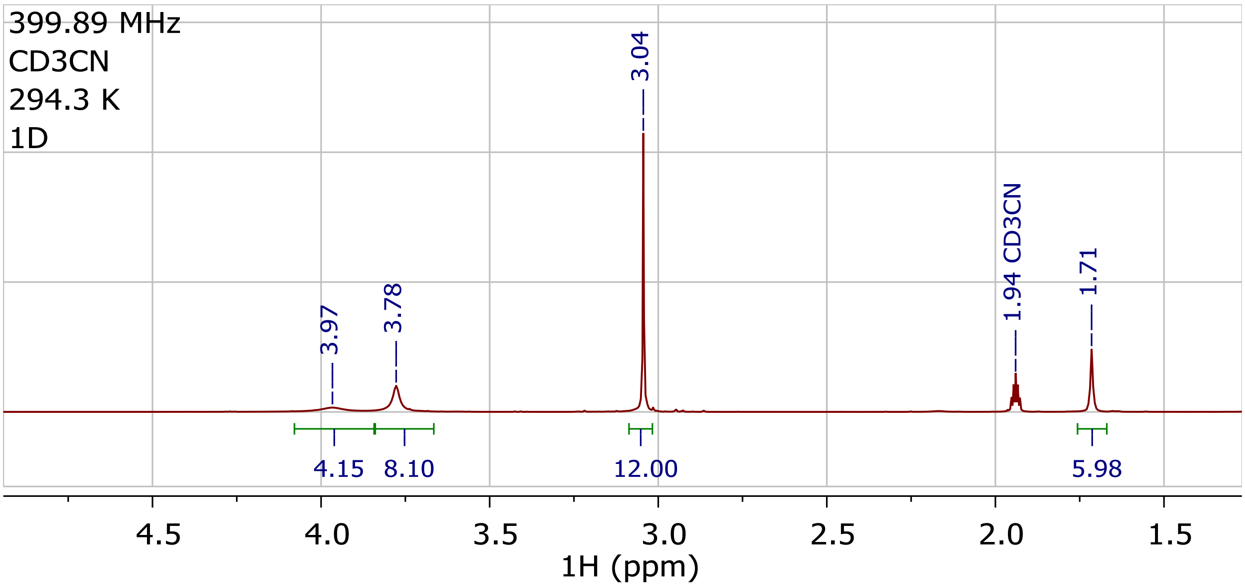

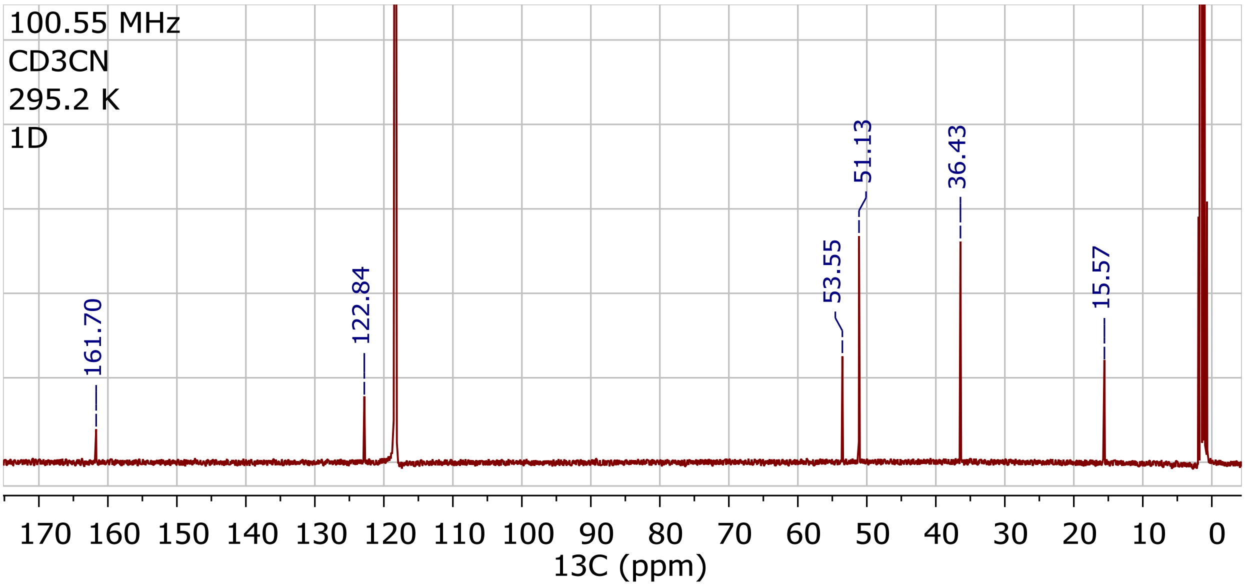

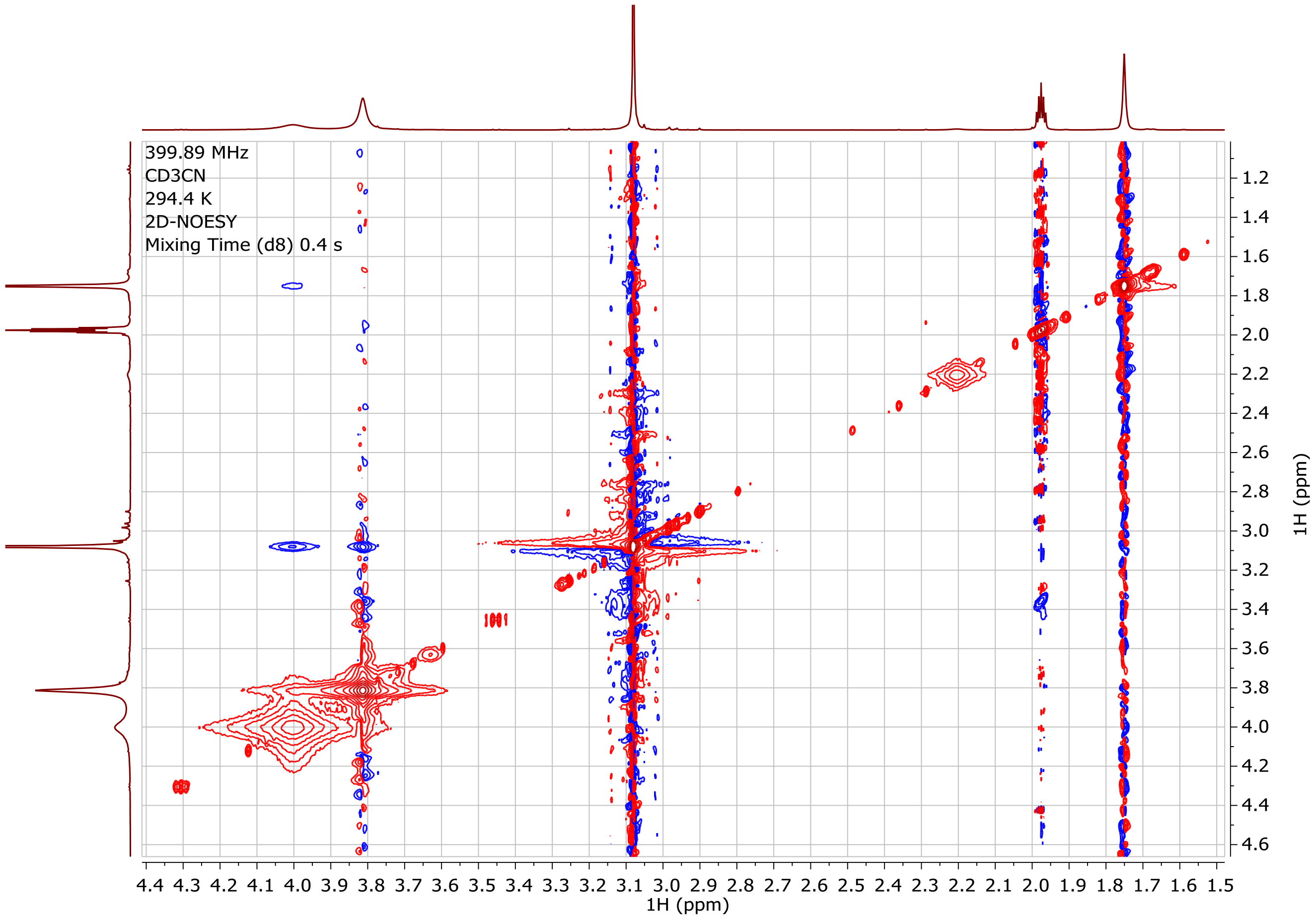
399.89 MHz
CD3CN
294.4 K
2D-NOESY
Mixing Time (d8) 0.4 s
1H (ppm)
1H (ppm)

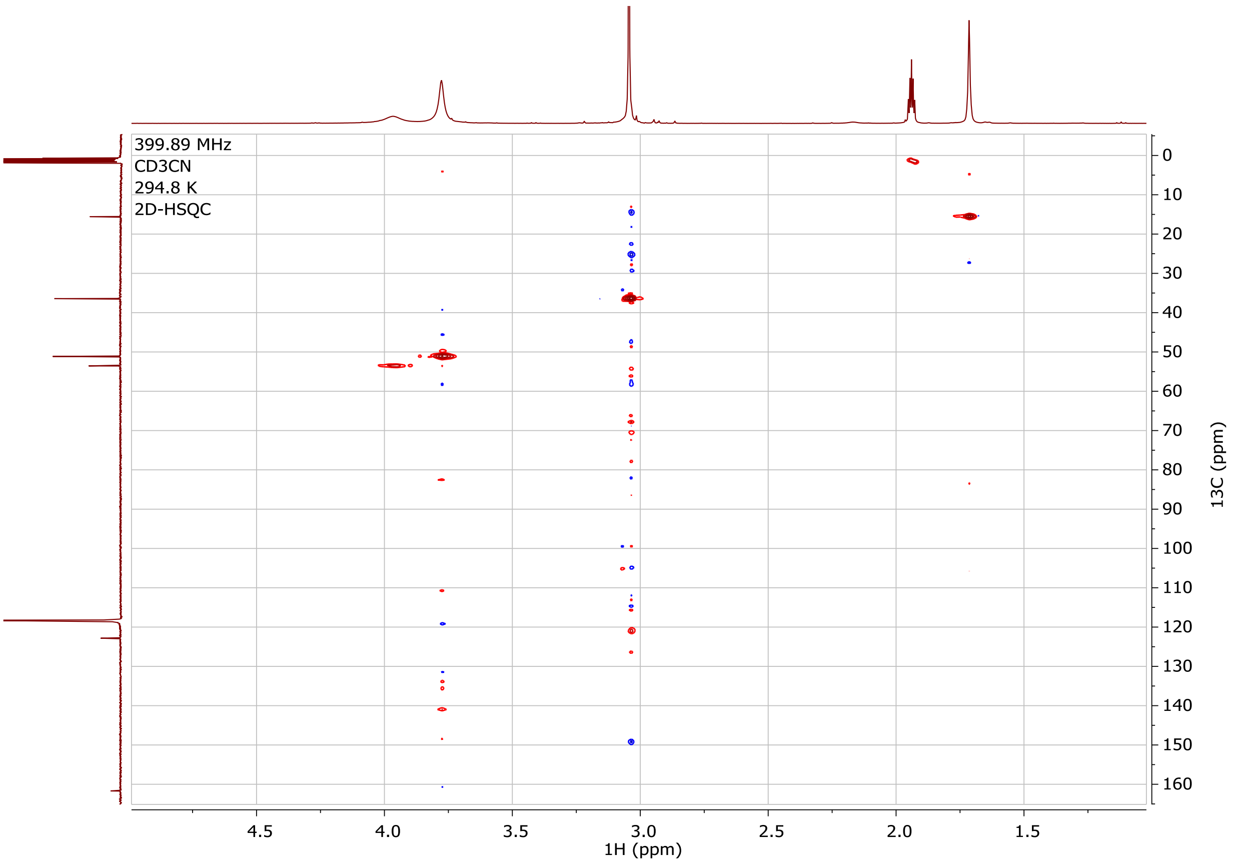
399.89 MHz
CD3CN
294.8 K
2D-HSQC
1H (ppm)
13C (ppm)

3(BF$_4$)$_2$

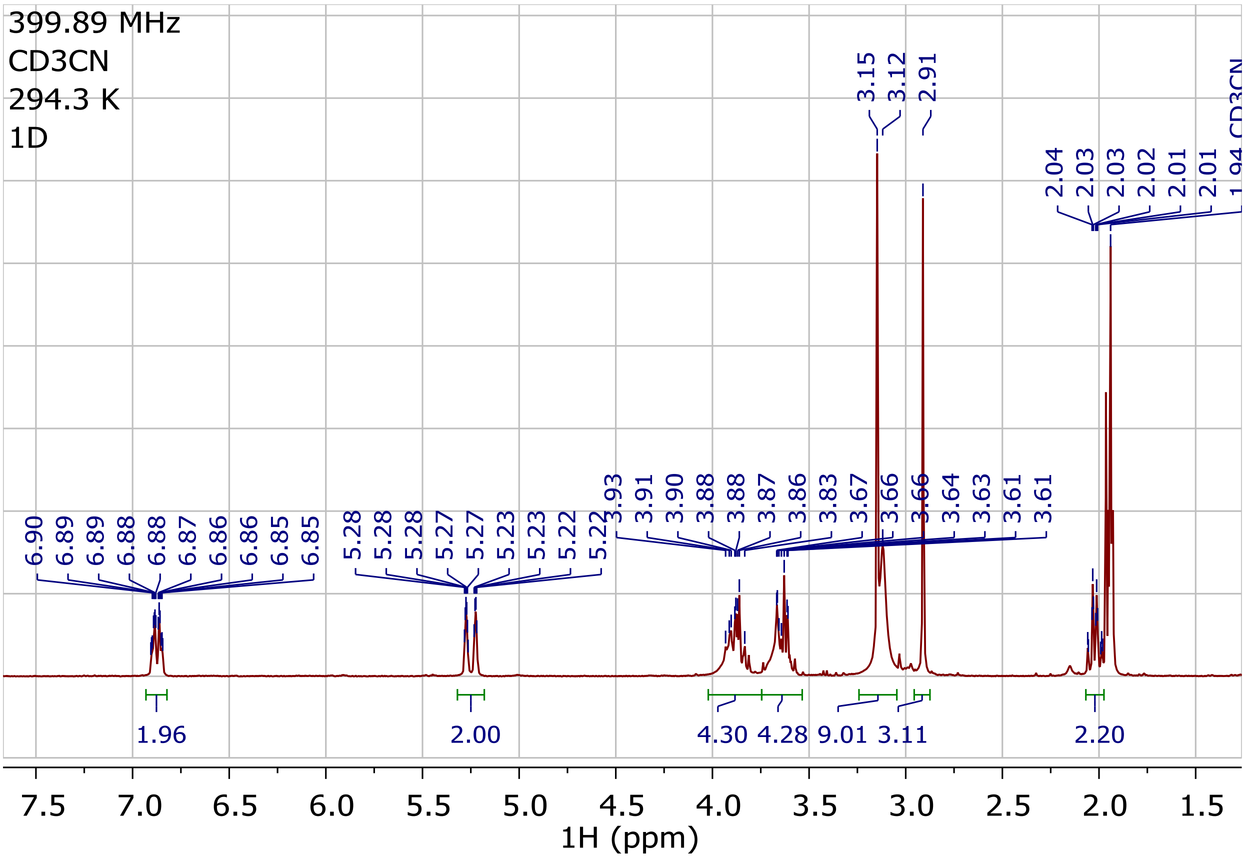
399.89 MHz
CD3CN
294.3 K
1D
3.15
3.12
2.91
2.04
2.03
2.03
2.02
2.01
2.01
1.94 CD3CN
6.90
6.89
6.89
6.88
6.88
6.87
6.86
6.86
6.85
6.85
5.28
5.28
5.28
5.27
5.27
5.23
5.23
5.22
5.22
3.93
3.91
3.90
3.88
3.88
3.87
3.86
3.83
3.67
3.66
3.66
3.64
3.63
3.61
3.61
1.96
2.00
4.30
4.28
9.01
3.11
2.20
7.5 7.0 6.5 6.0 5.5 5.0 4.5 4.0 3.5 3.0 2.5 2.0 1.5
1H (ppm)

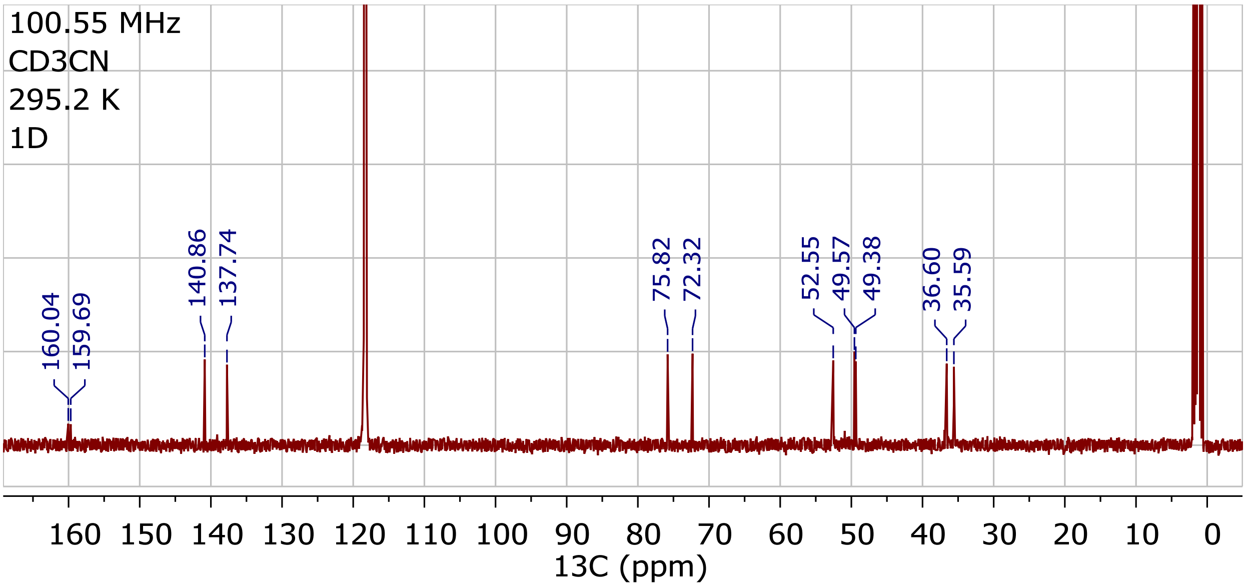
100.55 MHz
CD3CN
295.2 K
1D
160.04
159.69
140.86
137.74
75.82
72.32
52.55
49.57
49.38
36.60
35.59
160 150 140 130 120 110 100 90 80 70 60 50 40 30 20 10 0
13C (ppm)

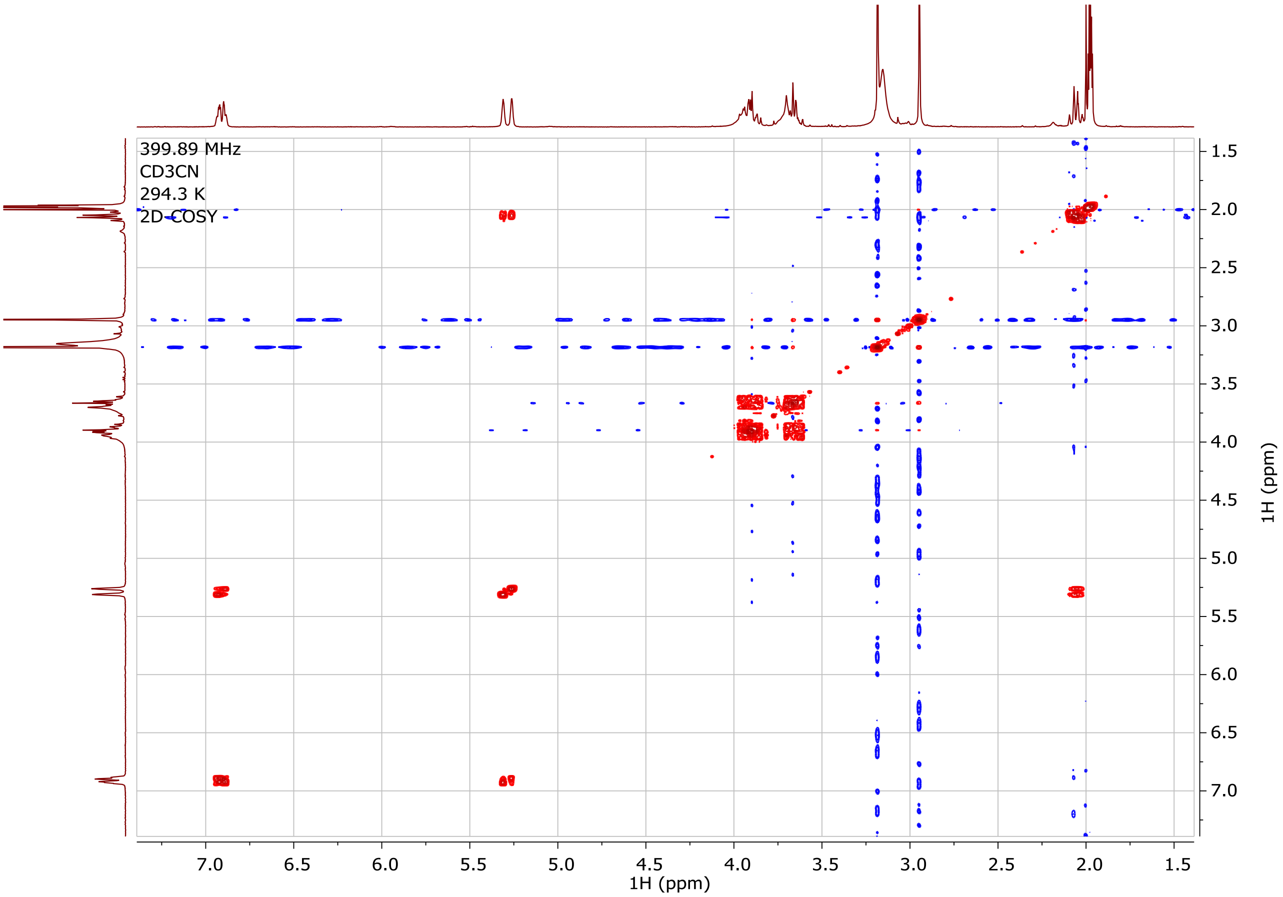
399.89 MHz
CD3CN
294.3 K
2D-COSY
1H (ppm)
1H (ppm)

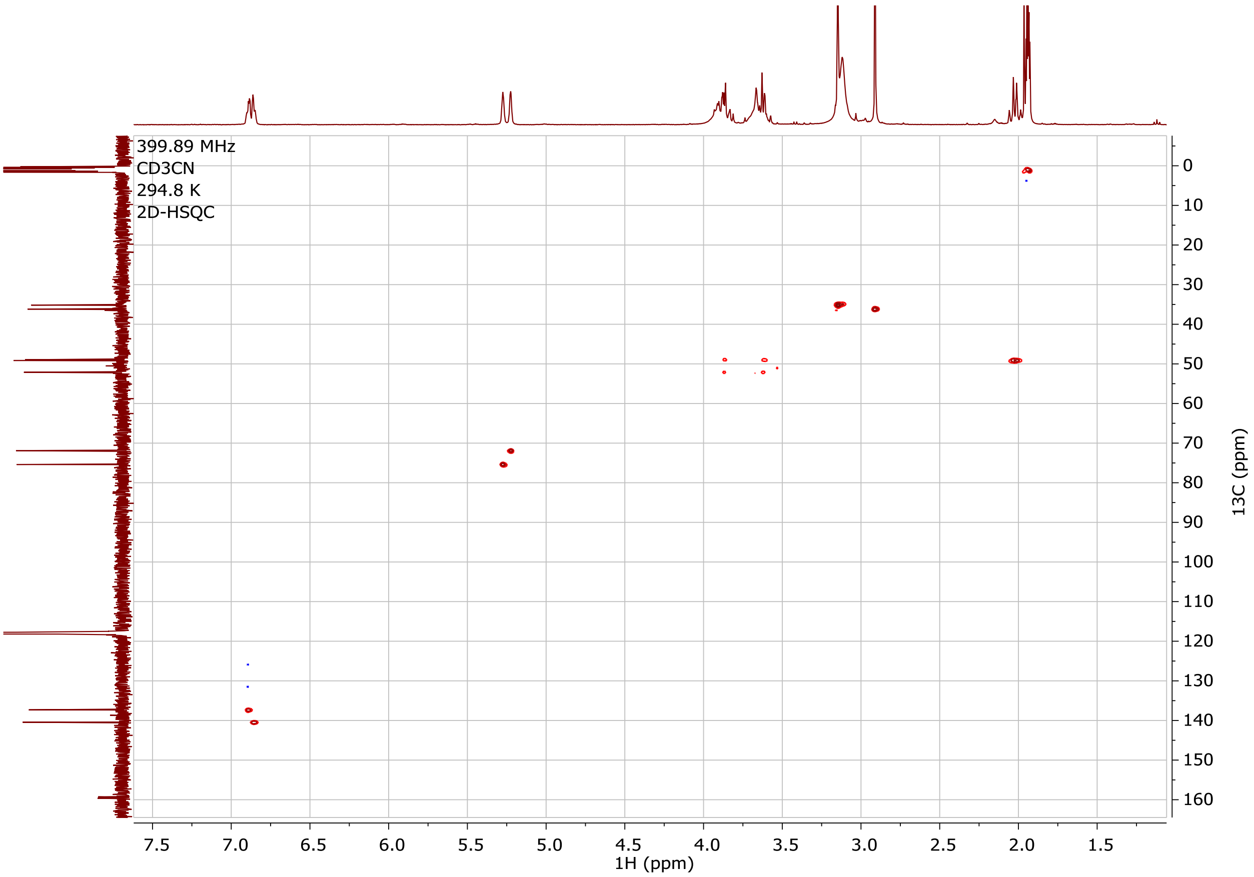
399.89 MHz
CD3CN
294.8 K
2D-HSQC
1H (ppm)
13C (ppm)

4(BF4)2

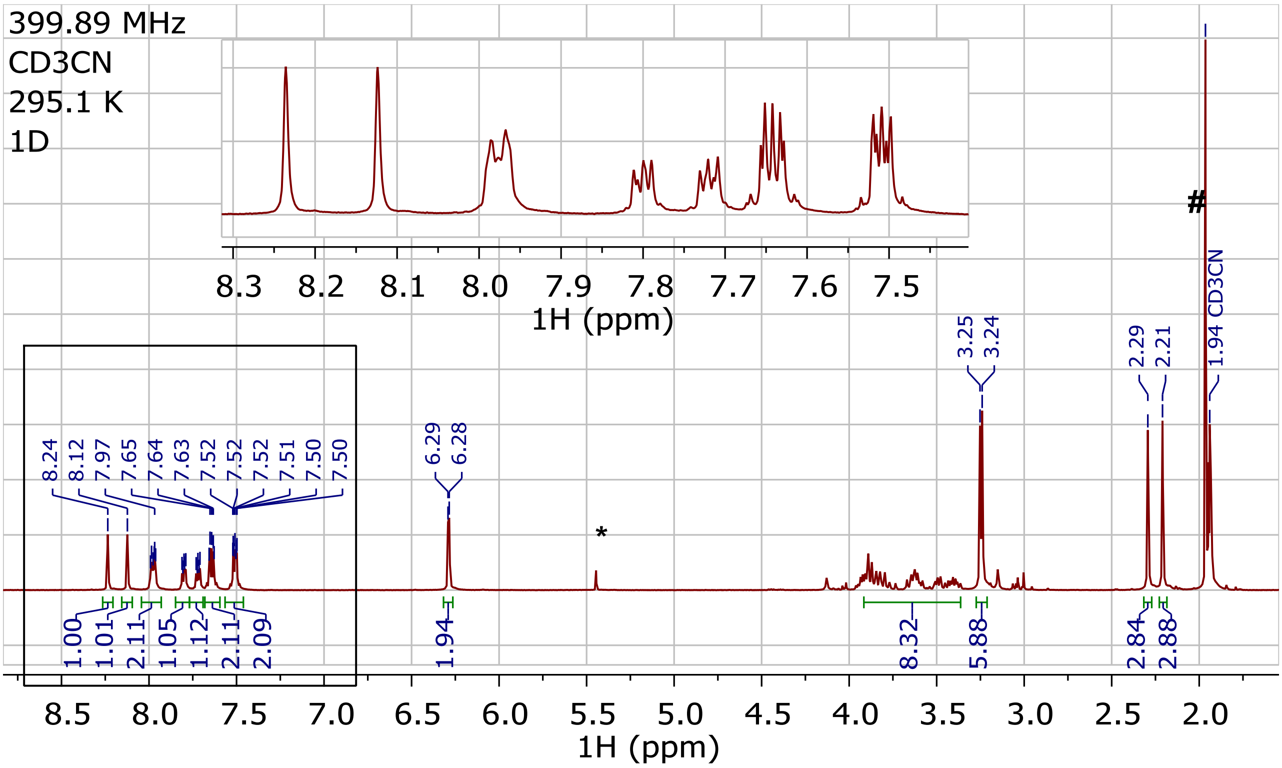

* marks traces of DCM, # marks traces of MeCN

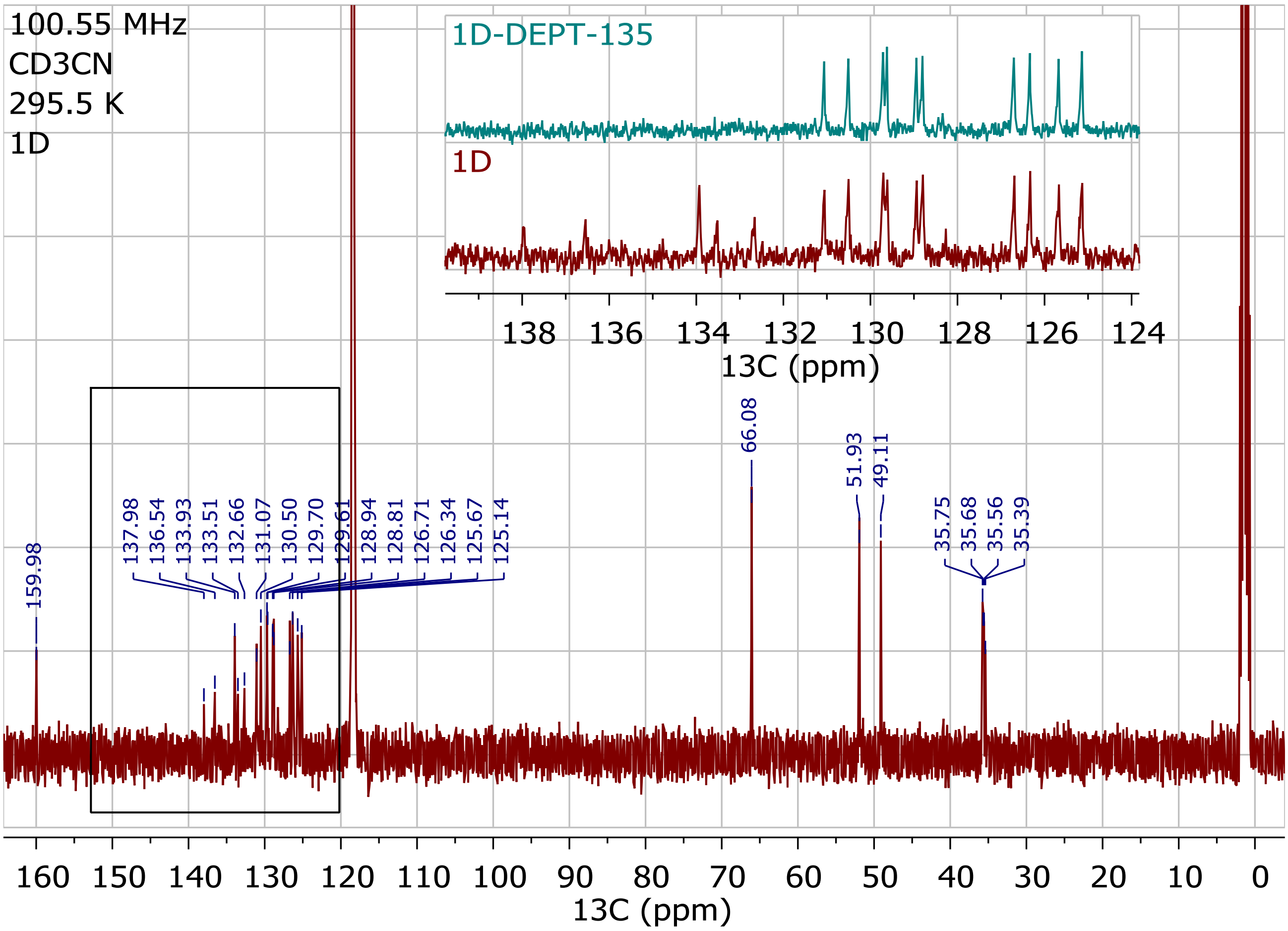

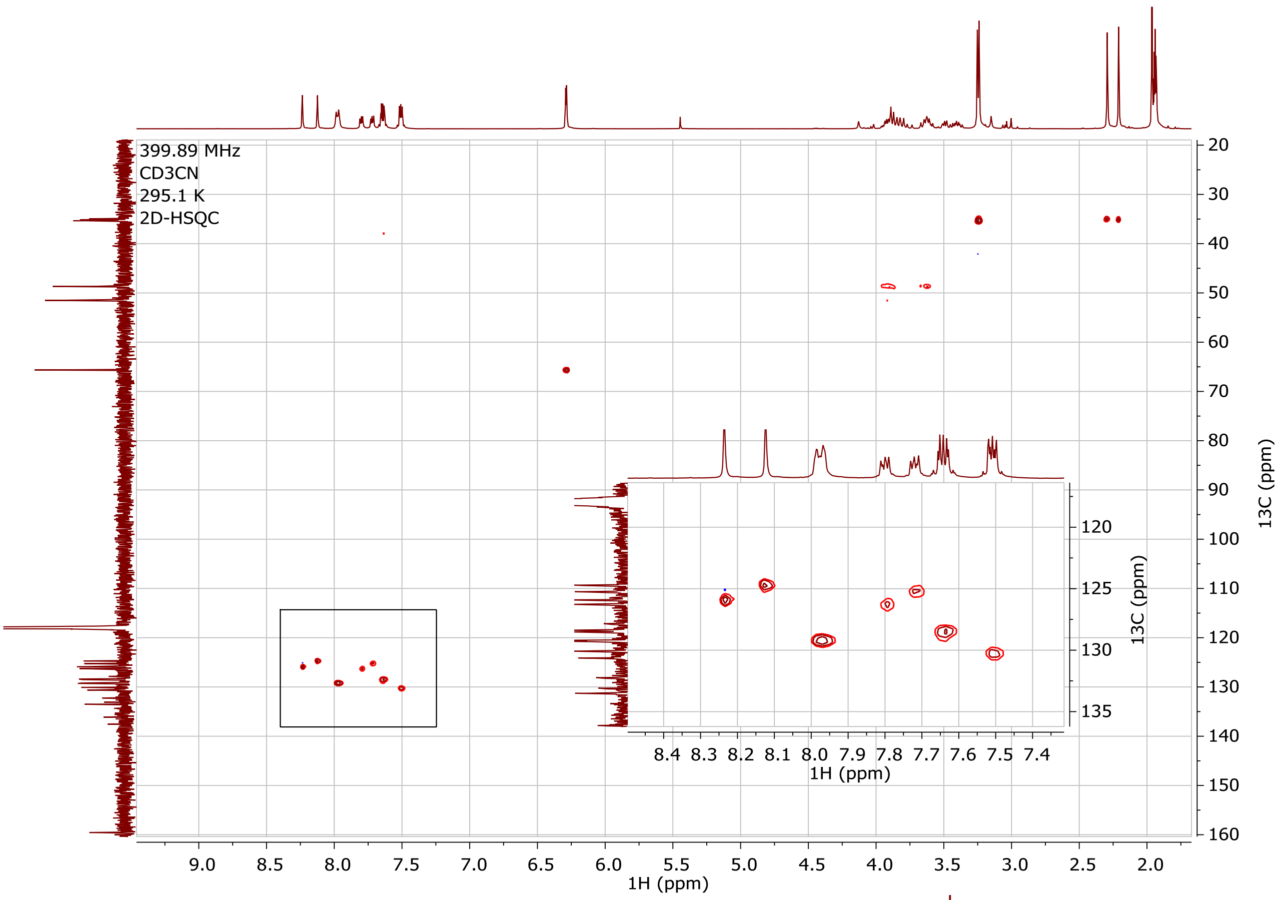
399.89 MHz
CD3CN
295.1 K
2D-HSQC
1H (ppm)
13C (ppm)

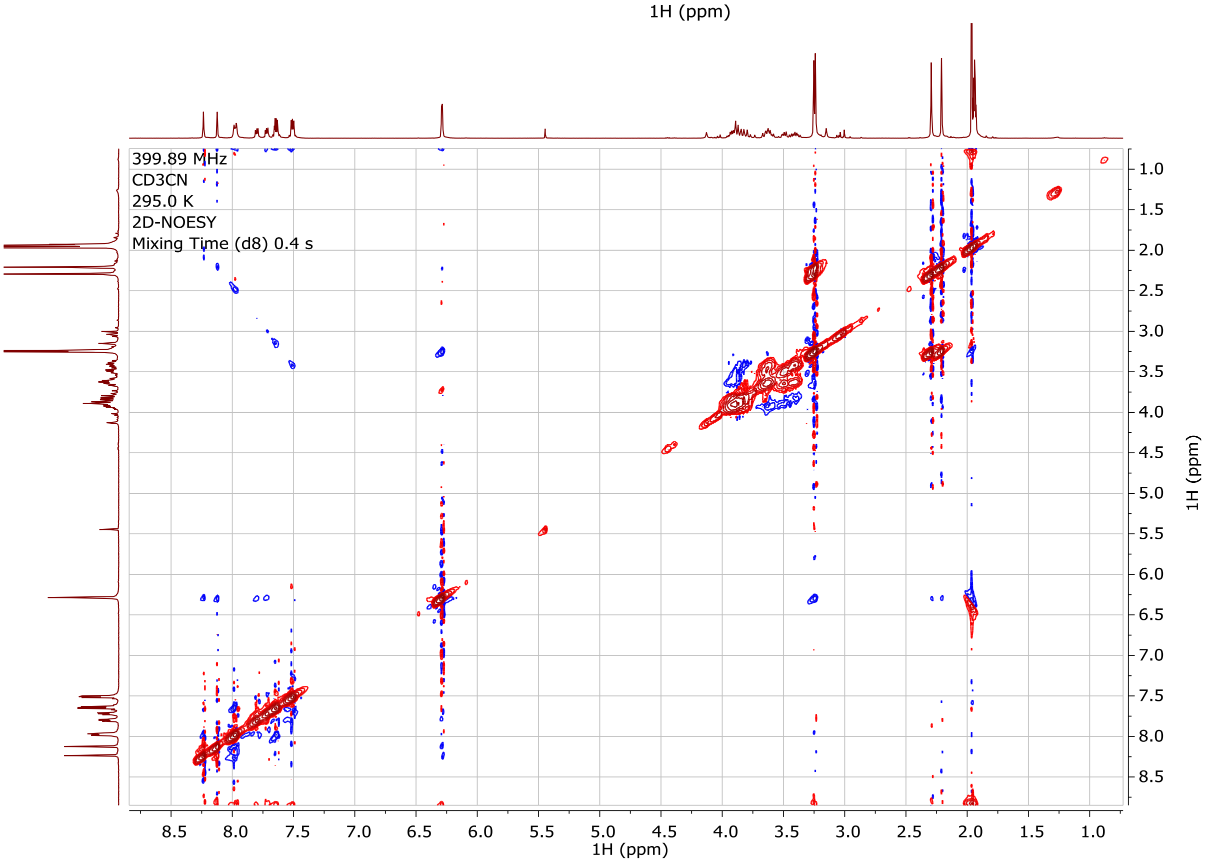
399.89 MHz
CD3CN
295.0 K
2D-NOESY
Mixing Time (d8) 0.4 s
1H (ppm)
1H (ppm)

5(BF_4)$_2$

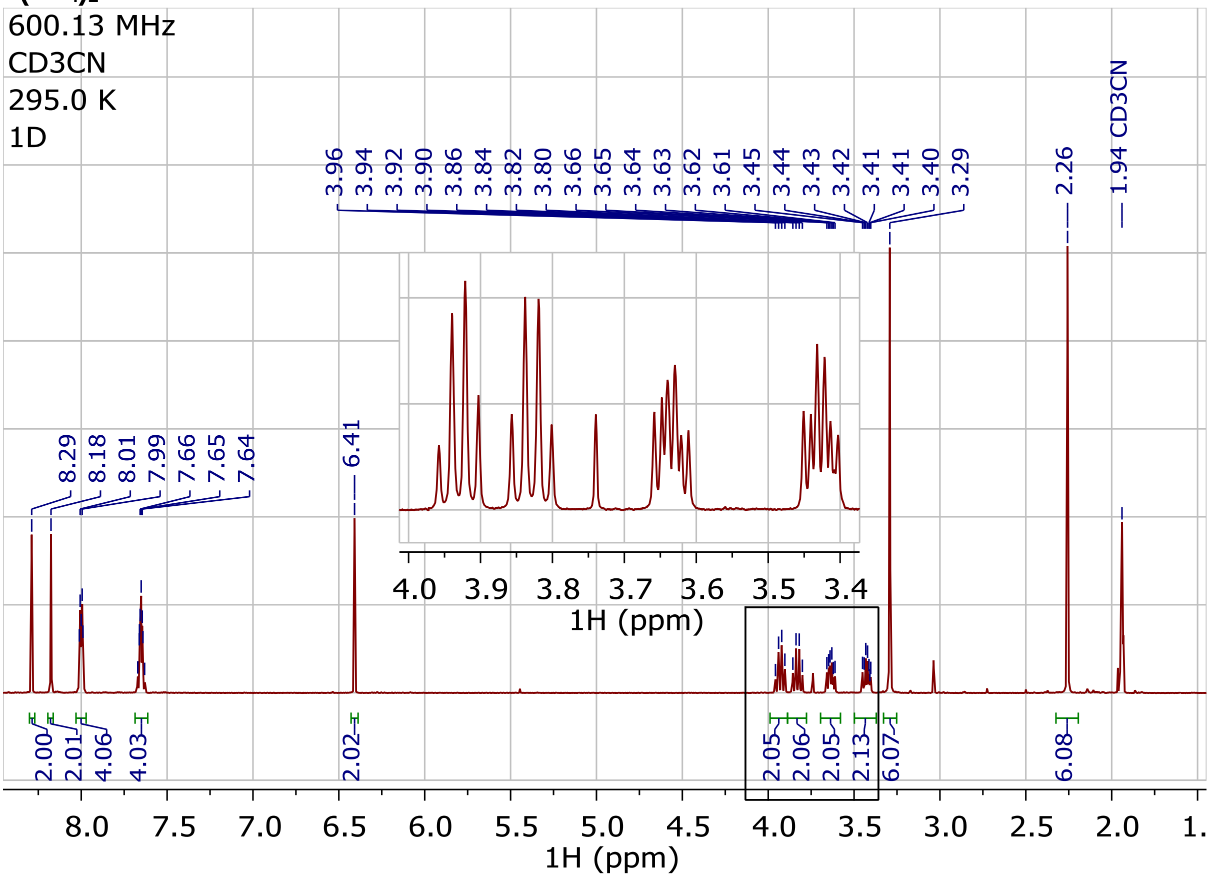
600.13 MHz
CD3CN
295.0 K
1D
8.29
8.18
8.01
7.99
7.66
7.65
7.64
6.41
3.96
3.94
3.92
3.90
3.86
3.84
3.82
3.80
3.66
3.65
3.64
3.63
3.62
3.61
3.45
3.44
3.43
3.42
3.41
3.41
3.40
3.29
2.26
1.94 CD3CN
2.00
2.01
4.06
4.03
2.02
2.05
2.06
2.05
2.13
6.07
6.08
1H (ppm)

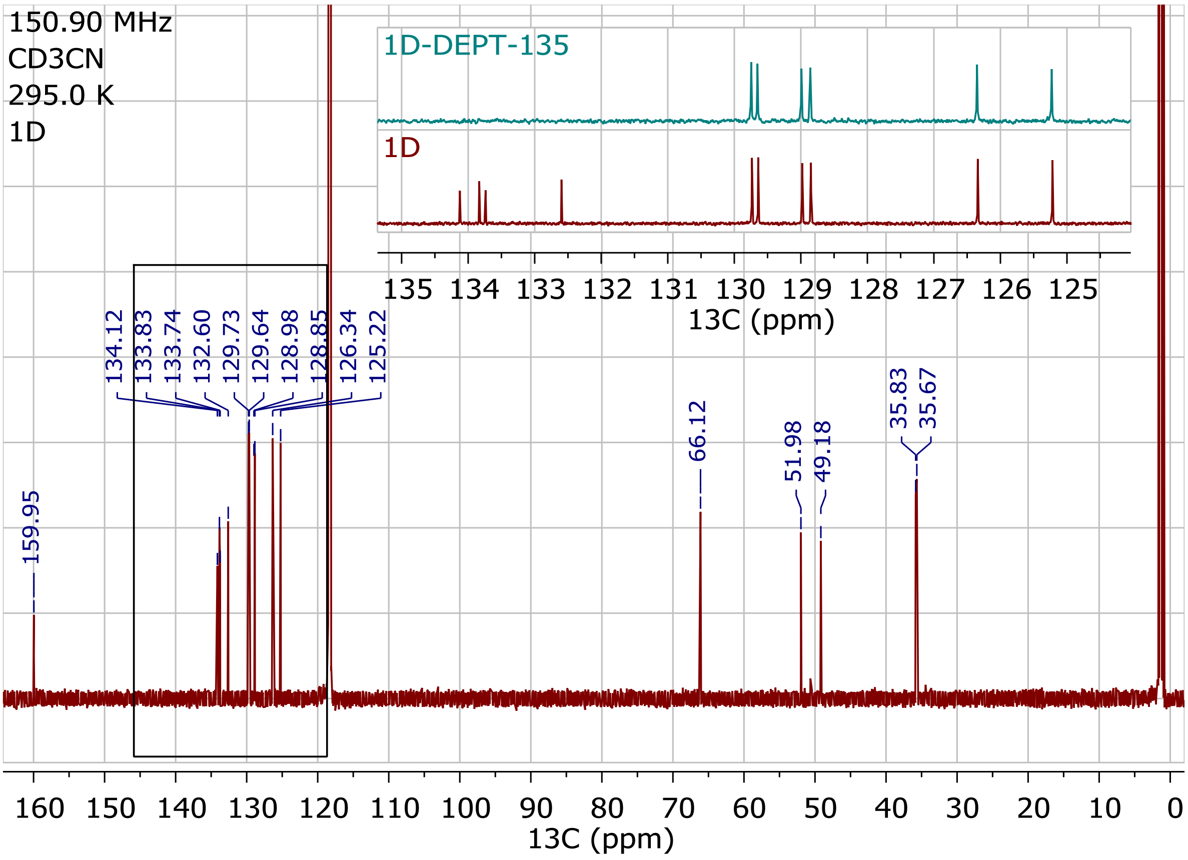
150.90 MHz
CD3CN
295.0 K
1D
1D-DEPT-135
1D
159.95
134.12
133.83
133.74
132.60
129.73
129.64
128.98
128.85
126.34
125.22
66.12
51.98
49.18
35.83
35.67
13C (ppm)

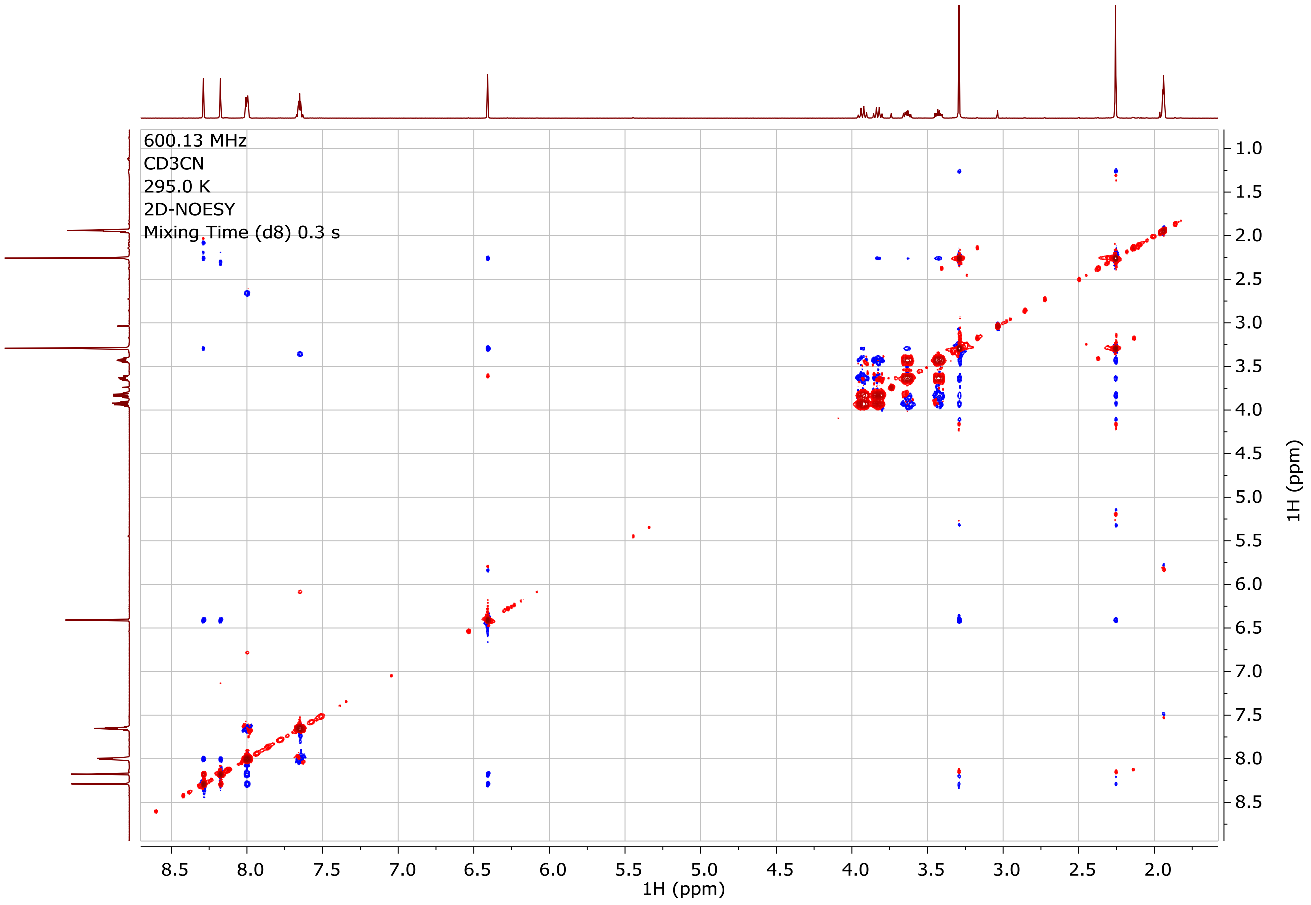
600.13 MHz
CD3CN
295.0 K
2D-NOESY
Mixing Time (d8) 0.3 s
1H (ppm)
1H (ppm)

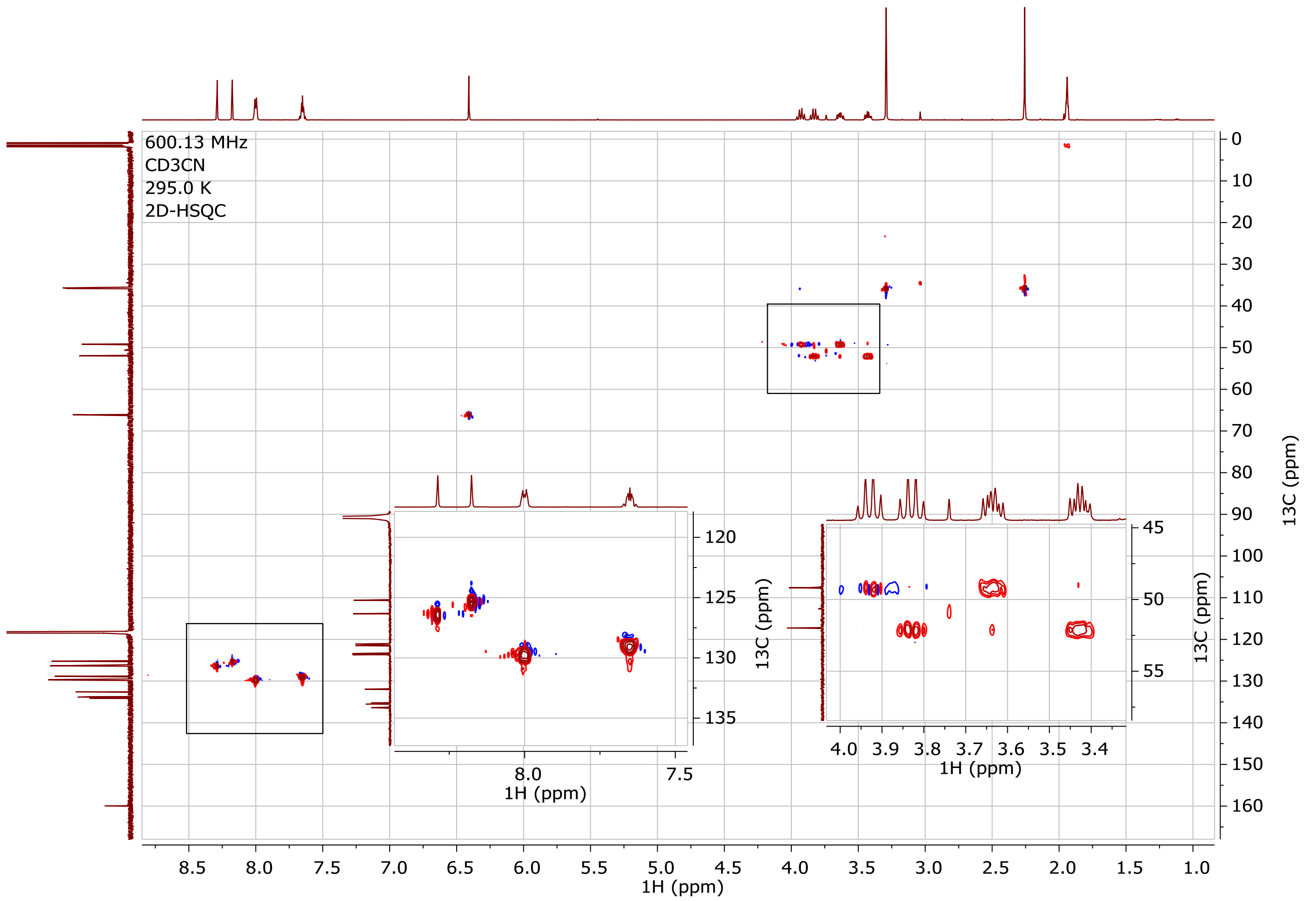
600.13 MHz
CD3CN
295.0 K
2D-HSQC
13C (ppm)
1H (ppm)
13C (ppm)
1H (ppm)
13C (ppm)
1H (ppm)

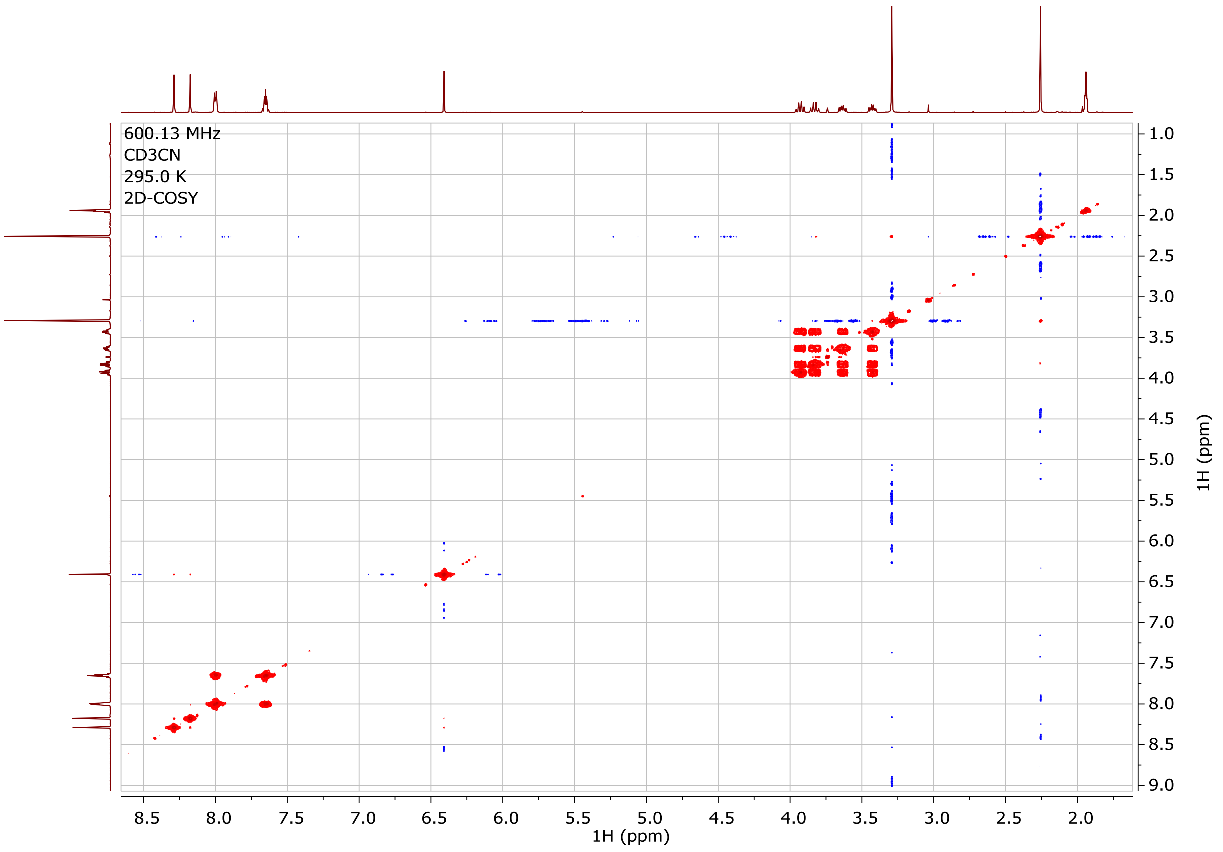
600.13 MHz
CD3CN
295.0 K
2D-COSY
1H (ppm)
1H (ppm)

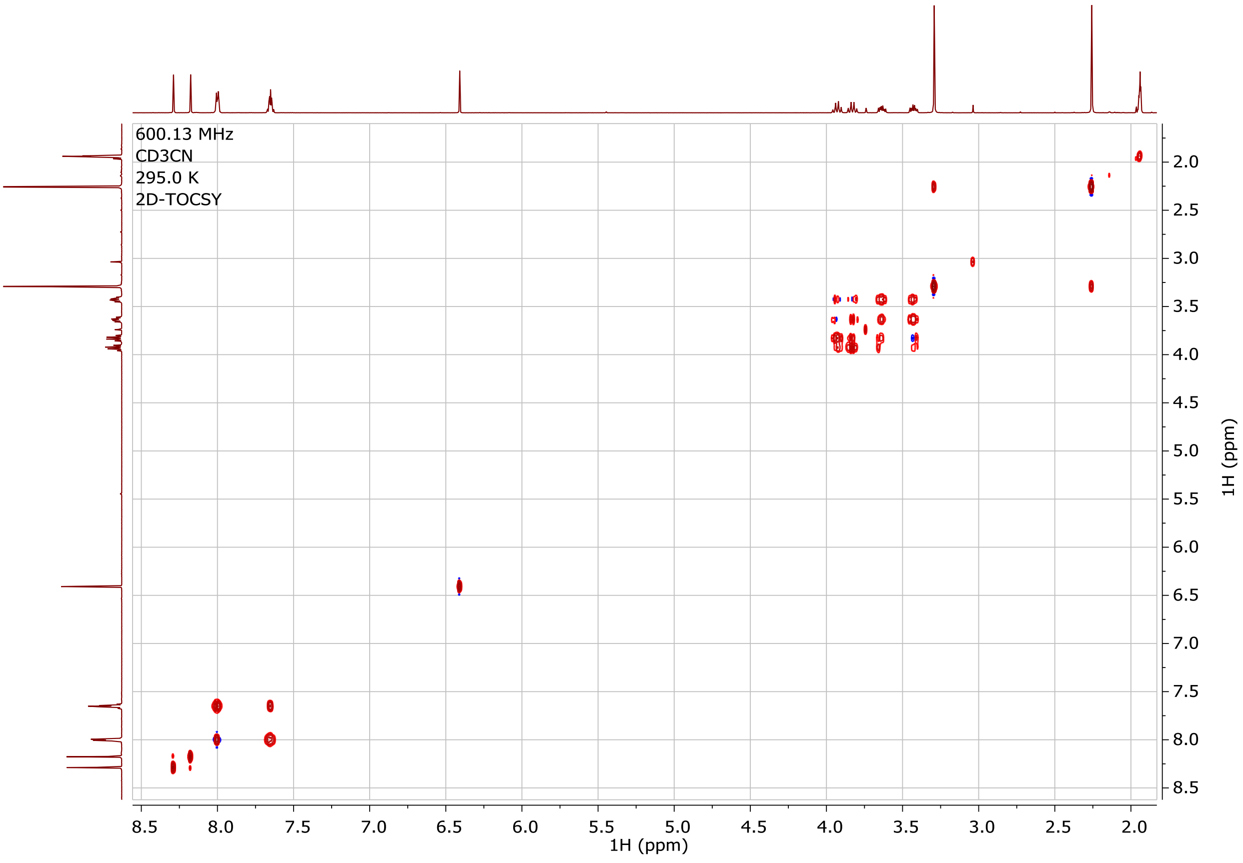
600.13 MHz
CD3CN
295.0 K
2D-TOCSY
1H (ppm)
1H (ppm)

8(SbF$_6$)$_2$

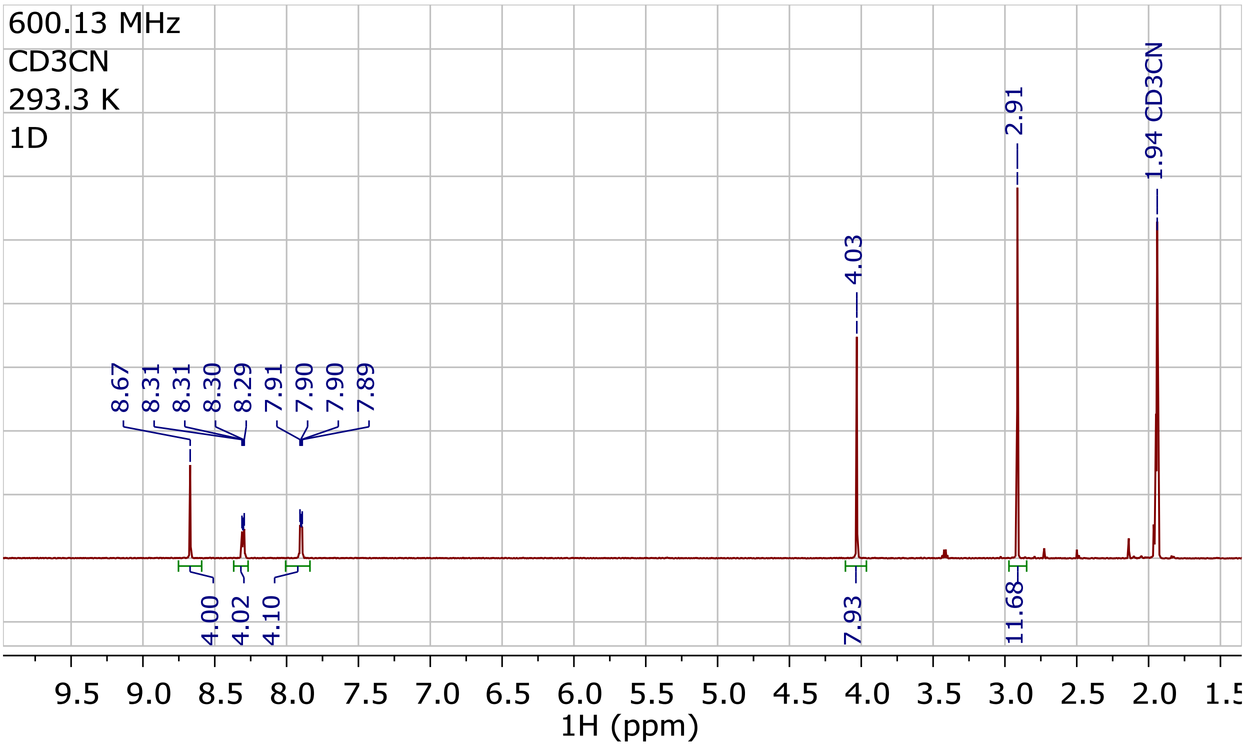
600.13 MHz
CD3CN
293.3 K
1D
8.67
8.31
8.31
8.30
8.29
7.91
7.90
7.90
7.89
4.03
2.91
1.94 CD3CN
4.00
4.02
4.10
7.93
11.68
9.5 9.0 8.5 8.0 7.5 7.0 6.5 6.0 5.5 5.0 4.5 4.0 3.5 3.0 2.5 2.0
1H (ppm)

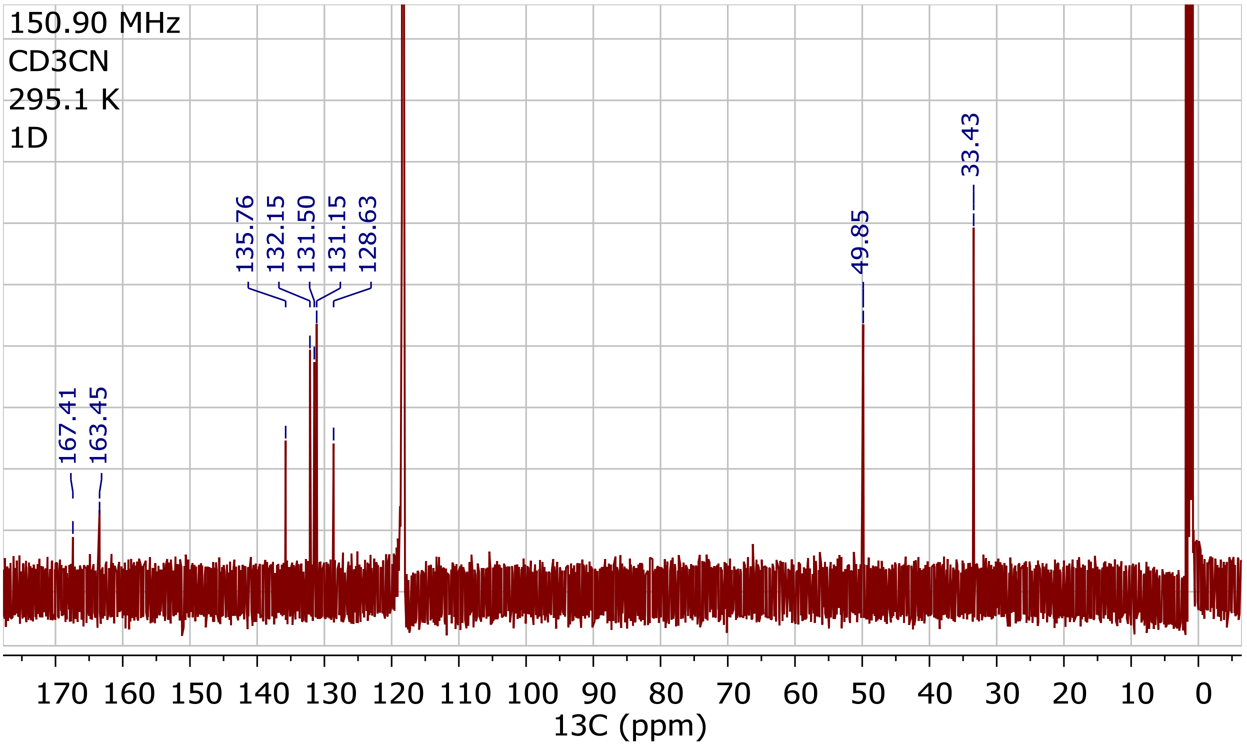
150.90 MHz
CD3CN
295.1 K
1D
167.41
163.45
135.76
132.15
131.50
131.15
128.63
49.85
33.43
170 160 150 140 130 120 110 100 90 80 70 60 50 40 30 20 10 0
13C (ppm)

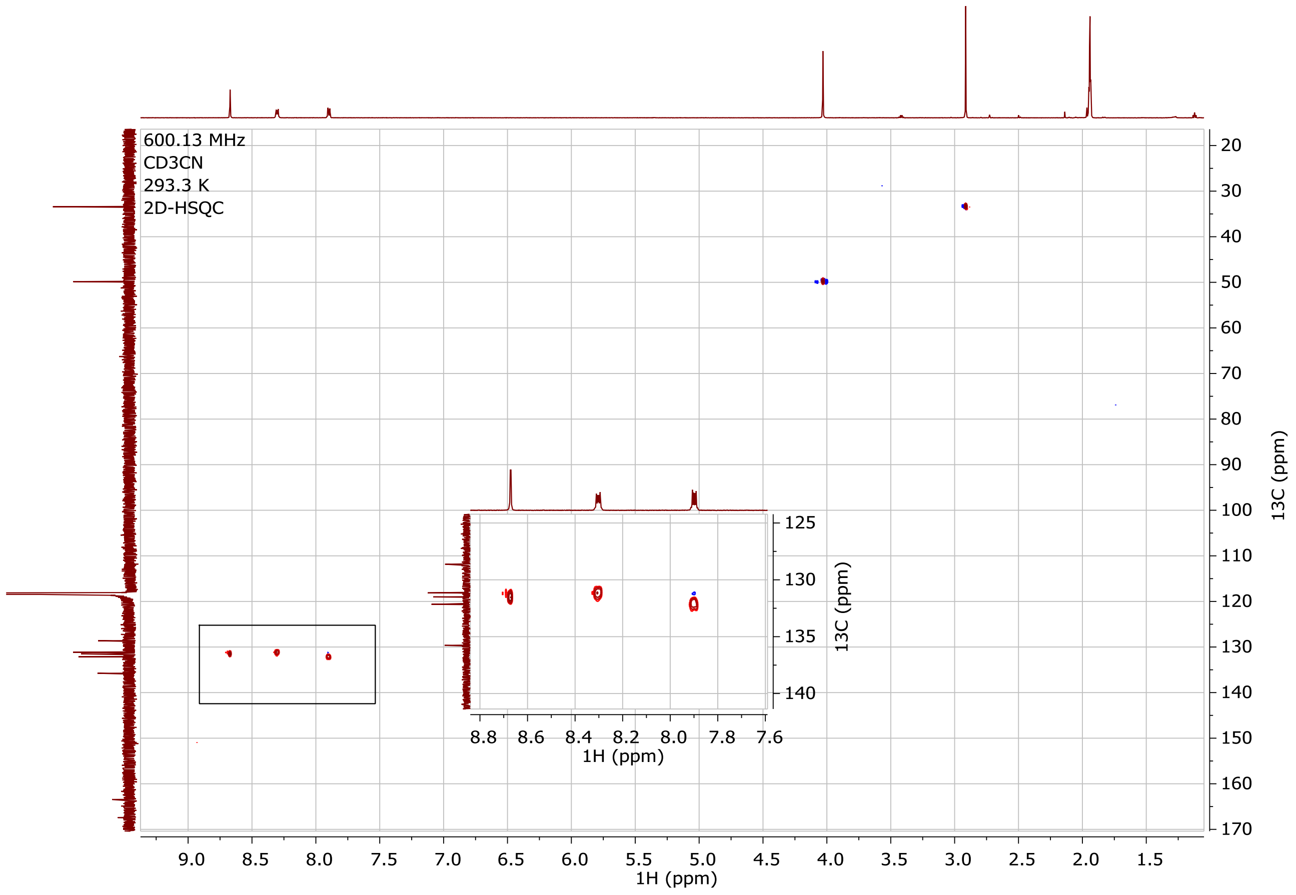
600.13 MHz
CD3CN
293.3 K
2D-HSQC
1H (ppm)
13C (ppm)

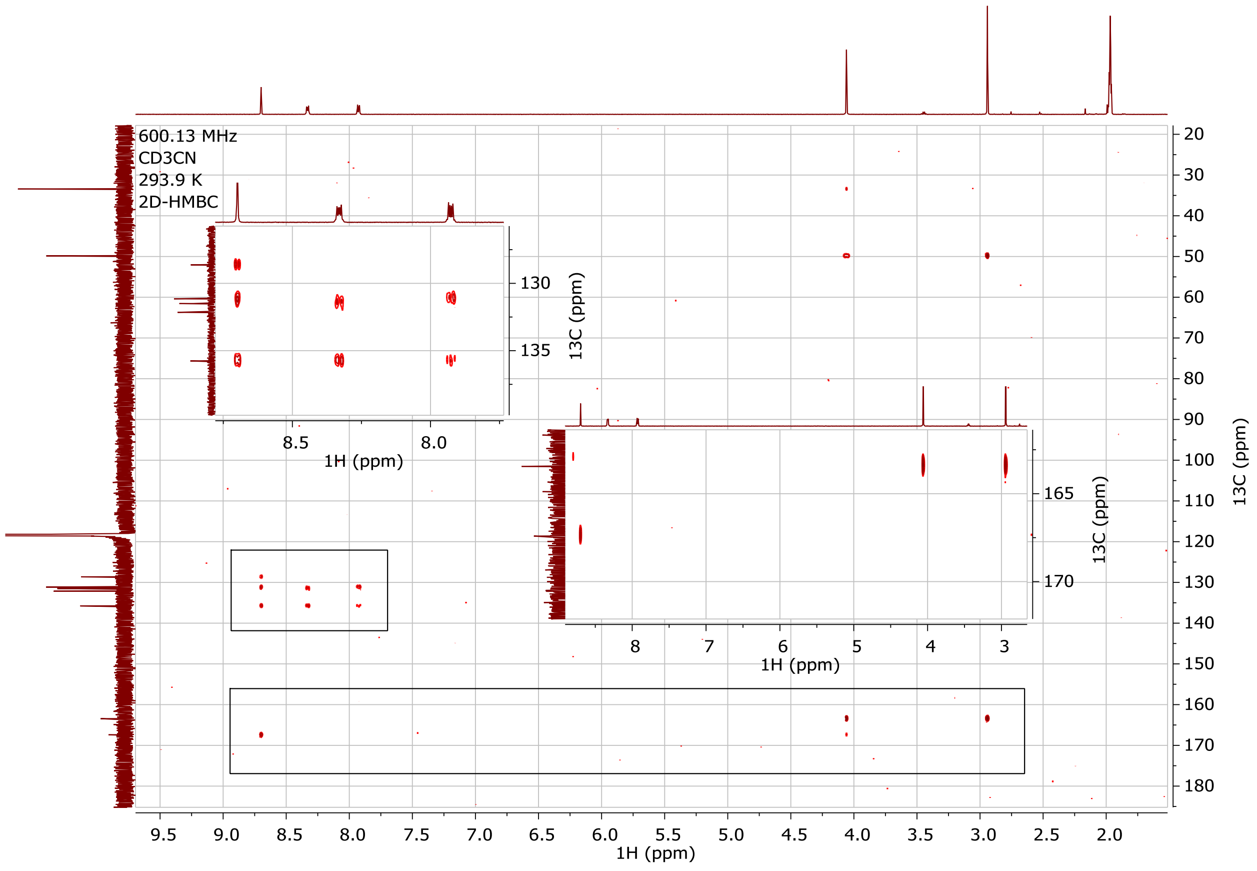
600.13 MHz
CD3CN
293.9 K
2D-HMBC
1H (ppm)
13C (ppm)

13. HR-MS spectra

HR-MS **3**$(BF_4)_2$

Analysis Info

Analysis Name	D:\data_2019\Himmel\icr35568_000001.d	Acquisition Date	06.08.2019 08:01:55
Method	ESI pos HPmix 200-1800	Instrument	ICR Apex-Qe
Sample Name	MW_118_1	Operator	I.Mitsch
Comment	Werr, AK Himmel: MW_118_1 in ACN		

Acquisition Parameters

Accumulations	16	Collision Gas Flow Rate	0.5 L/sec	Capillary Entrance	4200.0 V
Broadband Low Mass	173.2 m/z	Collision Energy	0.5 eV	Calibration Date	Wed Jul 17 03:06:43 2019
Broadband High Mass	2500.0 m/z	Collision Cell RF	1200.0 V		
Data Acquisition Size	2097152	Q1 Resolution	5.0		
		Q1 Mass	200.000 m/z		

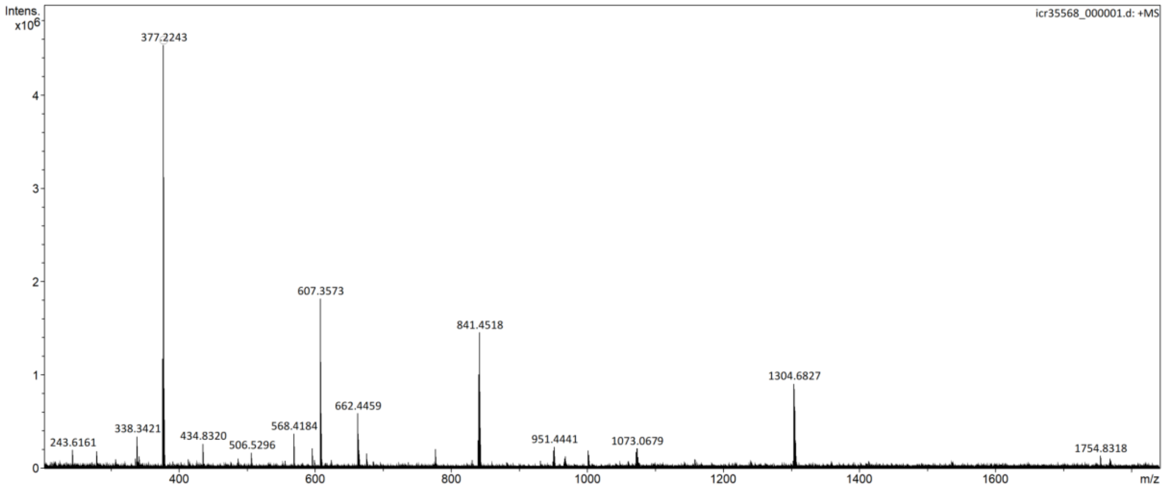

Mass Spectrum Formula Report

Analysis Info

Analysis Name	D:\data_2019\Himmel\icr35568_000001.d	Acquisition Date	06.08.2019 08:01:55
Comment	Werr, AK Himmel: MW_118_1 in ACN		

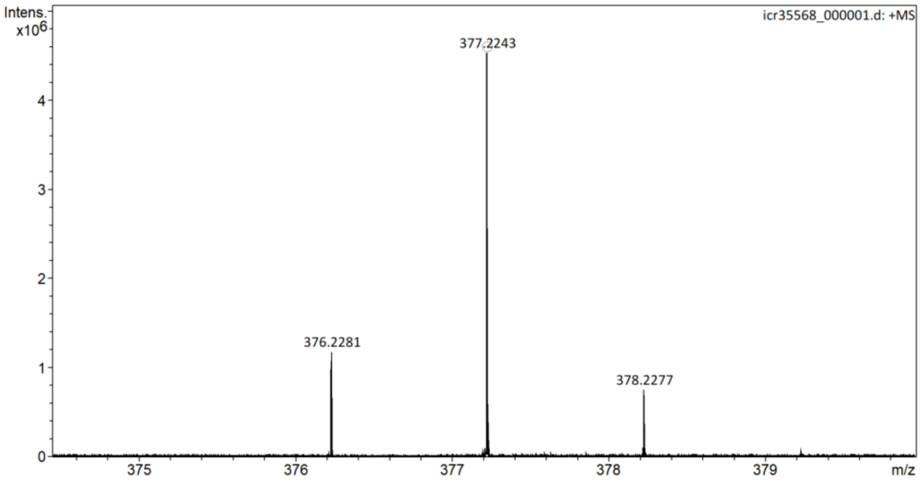

Meas. m/z	Ion Formula	m/z	err [ppm]	mSigma	rdb	e⁻ Conf	N-Rule
377.2243	C15H26BF4N6	377.2243	-0.2	17.4	4.5	even	ok
	C15H27F2N7O2	375.2189	-2687.5	952.0	5.0	odd	ok

HR-MS **4**$(BF_4)_2$

Analysis Info

Analysis Name	D:\data_2019\Himmel\icr36059_000001.d	Acquisition Date	27.09.2019 14:51:03
Method	ESI pos HPmix 200-1800	Instrument	ICR Apex-Qe
Sample Name	MW_134_1	Operator	I.Mitsch
Comment	Werr, AK Himmel: MW_134_1 in MeCN		

Acquisition Parameters

Accumulations	16	Collision Gas Flow Rate	0.5 L/sec	Capillary Entrance	4200.0 V
Broadband Low Mass	173.2 m/z	Collision Energy	0.5 eV	Calibration Date	Mon Sep 2 01:52:56 2019
Broadband High Mass	2500.0 m/z	Collision Cell RF	1200.0 V		
Data Acquisition Size	2097152	Q1 Resolution	5.0		
		Q1 Mass	200.000 m/z		

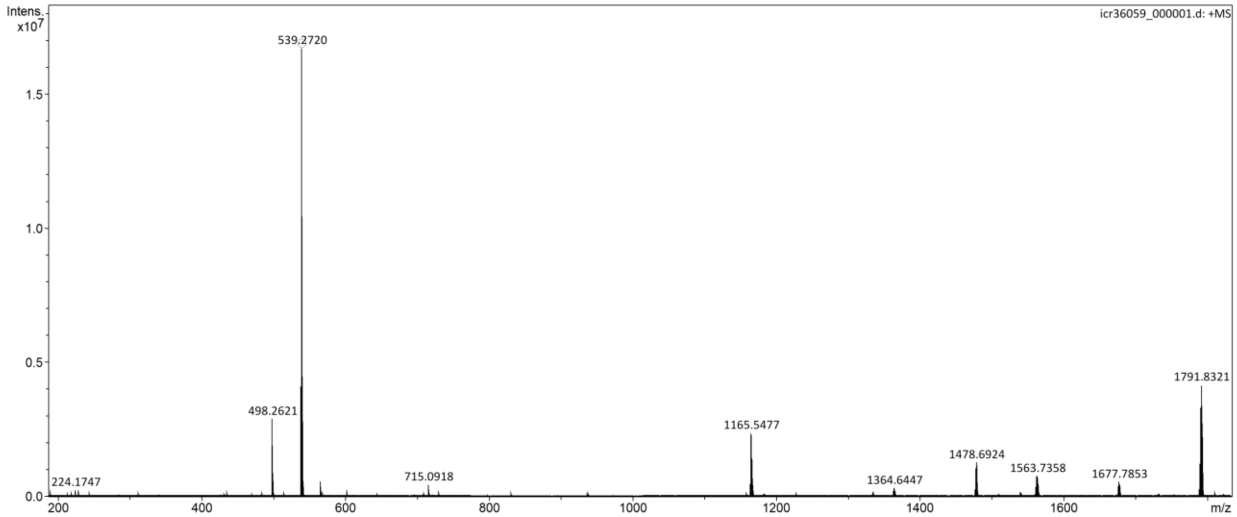

Mass Spectrum Formula Report

Analysis Info

Analysis Name	D:\data_2019\Himmel\icr36059_000001.d	Acquisition Date	27.09.2019 14:51:03
Comment	Werr, AK Himmel: MW_134_1 in MeCN		

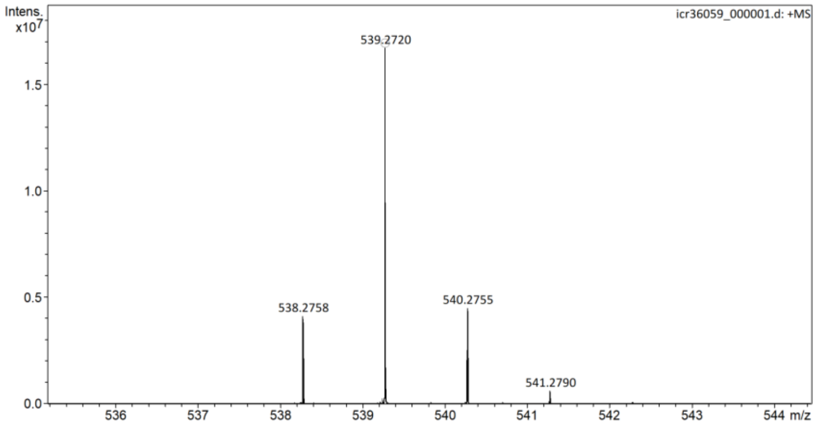

Meas. m/z	Ion Formula	m/z	err [ppm]	mSigma	rdb	e⁻ Conf	N-Rule
539.2720	C28H38F3N2O5	539.2727	1.3	22.1	9.5	even	ok
	C28H31BFN9O	539.2723	0.5	32.5	18.0	odd	ok
	C28H32BF4N6	539.2712	-1.5	33.3	14.5	even	ok
	C28H35B2F6O2	539.2722	0.3	107.4	9.5	even	ok

HR-MS **5**$(BF_4)_2$

Analysis Info

Analysis Name	D:\data_2019\Himmel\icr34851_000001.d	Acquisition Date	05.06.2019 09:35:06
Method	ESI pos HPmix 200-1800	Instrument	ICR Apex-Qe
Sample Name	MW_107_5	Operator	I.Mitsch
Comment	Werr, AK Himmel: MW_107_5 in MeCN		

Acquisition Parameters

Accumulations	16	Collision Gas Flow Rate	0.5 L/sec	Capillary Entrance	4200.0 V
Broadband Low Mass	173.2 m/z	Collision Energy	0.5 eV	Calibration Date	Mon May 20 03:30:31 2019
Broadband High Mass	2500.0 m/z	Collision Cell RF	1200.0 V		
Data Acquisition Size	2097152	Q1 Resolution	5.0		
		Q1 Mass	200.000 m/z		

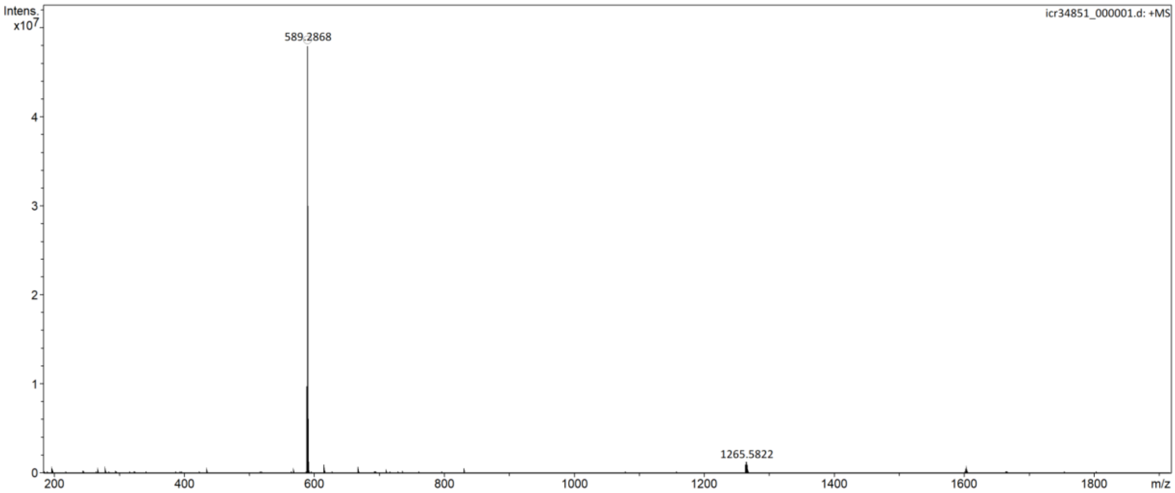

Mass Spectrum Formula Report

Analysis Info

Analysis Name	D:\data_2019\Himmel\icr34851_000001.d	Acquisition Date	05.06.2019 09:35:06
Comment	Werr, AK Himmel: MW_107_5 in MeCN		

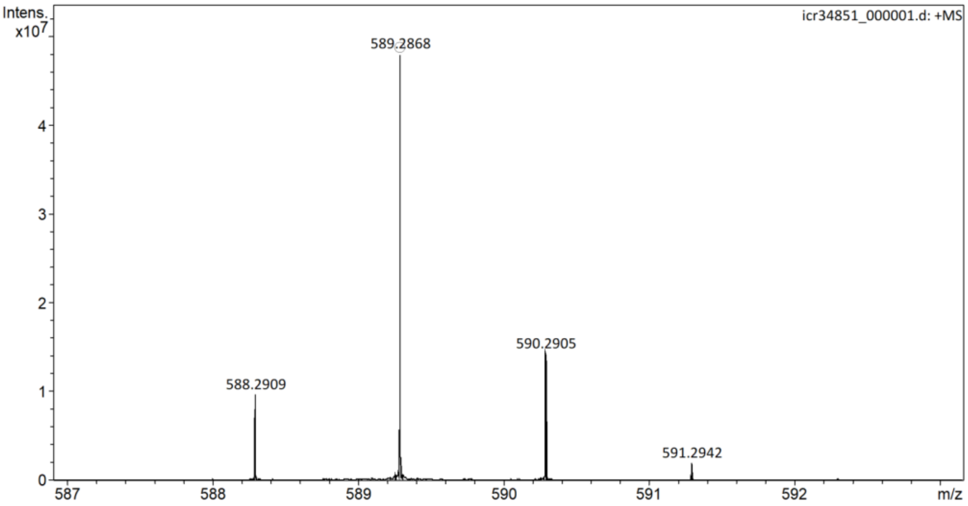

Meas. m/z	Ion Formula	m/z	err [ppm]	mSigma	rdb	e⁻ Conf	N-Rule
589.2868	C32H33BFN9O	589.2880	2.0	41.7	21.0	odd	ok
	C32H34BF4N6	589.2869	0.2	43.5	17.5	even	ok
	C32H37B2F6O2	589.2878	1.8	125.5	12.5	even	ok

HR-MS of reaction **5**(BF_4)$_2$ + 2eq KOtBu filtrate showing **7**$^{\cdot+}$

Analysis Info

Analysis Name	D:\data_2019\Himmel\icr36160_000001.d	Acquisition Date	09.10.2019 11:21:48
Method	ESI pos HPmix 200-1800	Instrument	ICR Apex-Qe
Sample Name	MW_137_1	Operator	I.Mitsch
Comment	Werr, AK Himmel: MW_137_1 in DCM		

Acquisition Parameters

Accumulations	16	Collision Gas Flow Rate	0.5 L/sec	Capillary Entrance	4200.0 V
Broadband Low Mass	173.2 m/z	Collision Energy	0.5 eV	Calibration Date	Tue Oct 1 01:19:47 2019
Broadband High Mass	2500.0 m/z	Collision Cell RF	1200.0 V		
Data Acquisition Size	2097152	Q1 Resolution	5.0		
		Q1 Mass	200.000 m/z		

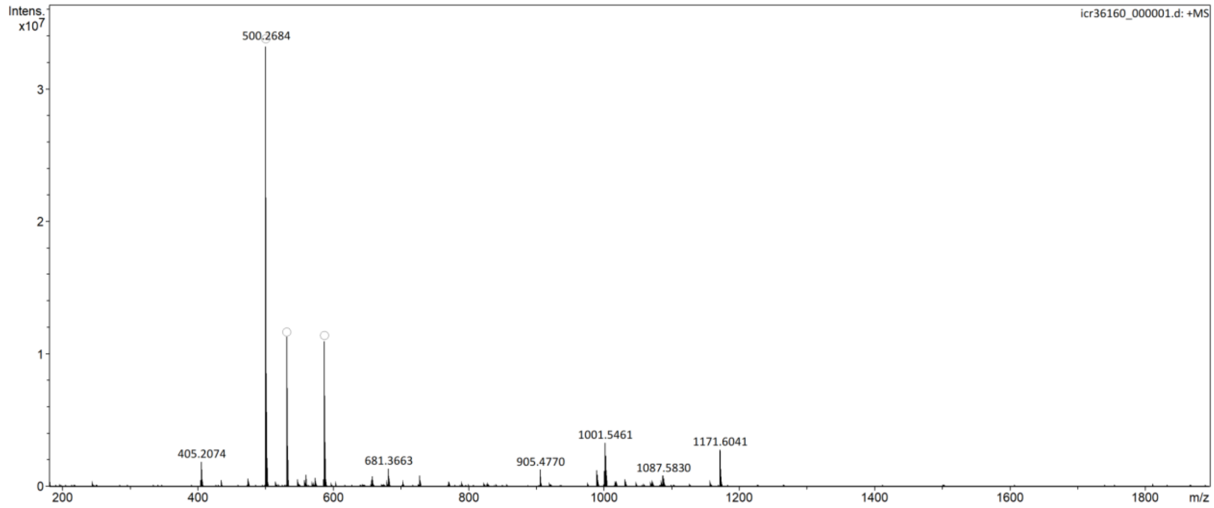

Mass Spectrum Formula Report

Analysis Info

Analysis Name	D:\data_2019\Himmel\icr36160_000001.d	Acquisition Date	09.10.2019 11:21:48
Comment	Werr, AK Himmel: MW_137_1 in DCM		

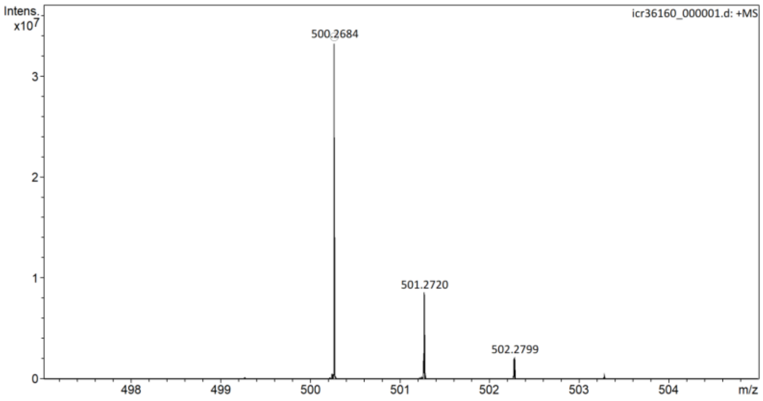

Meas. m/z	Ion Formula	m/z	err [ppm]	mSigma	rdb	e⁻ Conf	N-Rule
500.2684	C19H40N4O11	500.2688	0.8	21.0	2.0	odd	ok
	C18H34N11O6	500.2688	0.8	30.1	7.5	even	ok
	C17H38N7O10	500.2675	-1.8	30.9	2.5	even	ok
	C32H32N6	500.2683	-0.2	43.0	20.0	odd	ok
	C17H28N18O	500.2688	0.8	44.6	13.0	odd	ok
531.2869	C20H43N4O12	531.2872	0.5	22.1	1.5	even	ok
	C19H37N11O7	531.2872	0.5	31.4	7.0	odd	ok
	C18H41N7O11	531.2859	-2.0	32.1	2.0	odd	ok
	C33H35N6O	531.2867	-0.4	38.0	19.5	even	ok
	C18H31N18O2	531.2872	0.5	45.9	12.5	even	ok
587.3130	C23H47N4O13	587.3134	0.7	20.9	2.5	even	ok
	C22H41N11O8	587.3134	0.7	31.2	8.0	odd	ok
	C21H45N7O12	587.3121	-1.6	34.0	3.0	odd	ok
	C21H35N18O3	587.3134	0.6	43.9	13.5	even	ok
	C36H39N6O2	587.3129	-0.2	45.1	20.5	even	ok
	C38H41N3O3	587.3142	2.1	55.3	20.0	odd	ok

HR-MS of reaction **5**$(BF_4)_2$ + 2eq KOtBu residue showing $(\mathbf{7}+H)^+$

Analysis Info

Analysis Name	D:\data_2019\Himmel\icr36162_000001.d	Acquisition Date	09.10.2019 11:41:28
Method	ESI pos HPmix 200-1800	Instrument	ICR Apex-Qe
Sample Name	MW_137_2	Operator	I.Mitsch
Comment	Werr, AK Himmel: MW_137_2 in DCM		

Acquisition Parameters

Accumulations	16	Collision Gas Flow Rate	0.5 L/sec	Capillary Entrance	4200.0 V
Broadband Low Mass	173.2 m/z	Collision Energy	0.5 eV	Calibration Date	Tue Oct 1 01:19:47 2019
Broadband High Mass	2500.0 m/z	Collision Cell RF	1200.0 V		
Data Acquisition Size	2097152	Q1 Resolution	5.0		
		Q1 Mass	200.000 m/z		

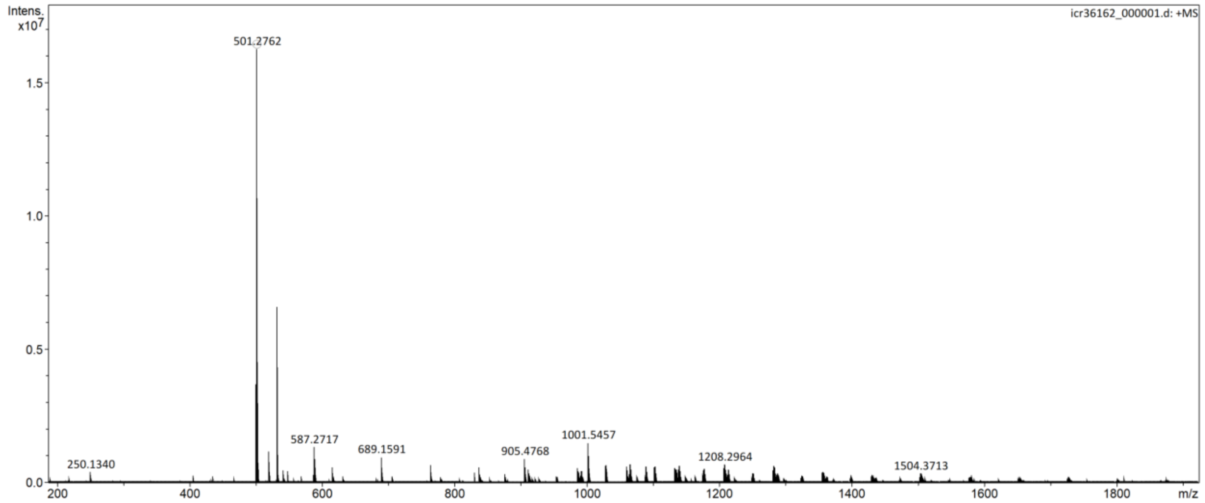

Mass Spectrum Formula Report

Analysis Info

Analysis Name	D:\data_2019\Himmel\icr36162_000001.d	Acquisition Date	09.10.2019 11:41:28
Comment	Werr, AK Himmel: MW_137_2 in DCM		

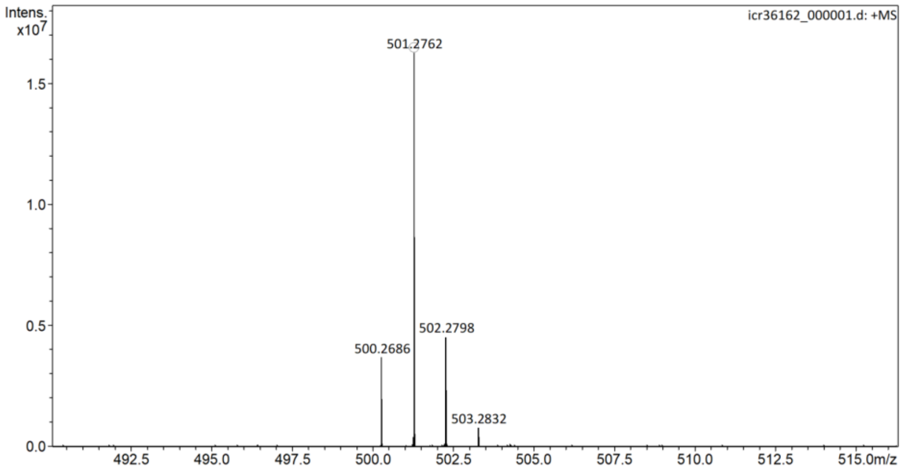

Meas. m/z	Ion Formula	m/z	err [ppm]	mSigma	rdb	e⁻ Conf	N-Rule
501.2762	C19H41N4O11	501.2766	0.9	32.0	1.5	even	ok
	C32H33N6	501.2761	-0.2	32.3	19.5	even	ok

HR-MS **8**(SbF_6)$_2$

Analysis Info

Analysis Name	Z:\Himmel\icr37203_000001.d	Acquisition Date	16.01.2020 11:22:14
Method	ESI pos HPmix 200-1800	Instrument	ICR Apex-Qe
Sample Name	MW_157_1	Operator	I.Mitsch
Comment	Werr, AK Himmel: MW_157_1 in MeCN		

Acquisition Parameters

Accumulations	16	Collision Gas Flow Rate	0.5 L/sec	Capillary Entrance	4200.0 V
Broadband Low Mass	173.2 m/z	Collision Energy	0.5 eV	Calibration Date	Tue Jan 14 02:13:10 2020
Broadband High Mass	2500.0 m/z	Collision Cell RF	1200.0 V		
Data Acquisition Size	2097152	Q1 Resolution	5.0		
		Q1 Mass	200.000 m/z		

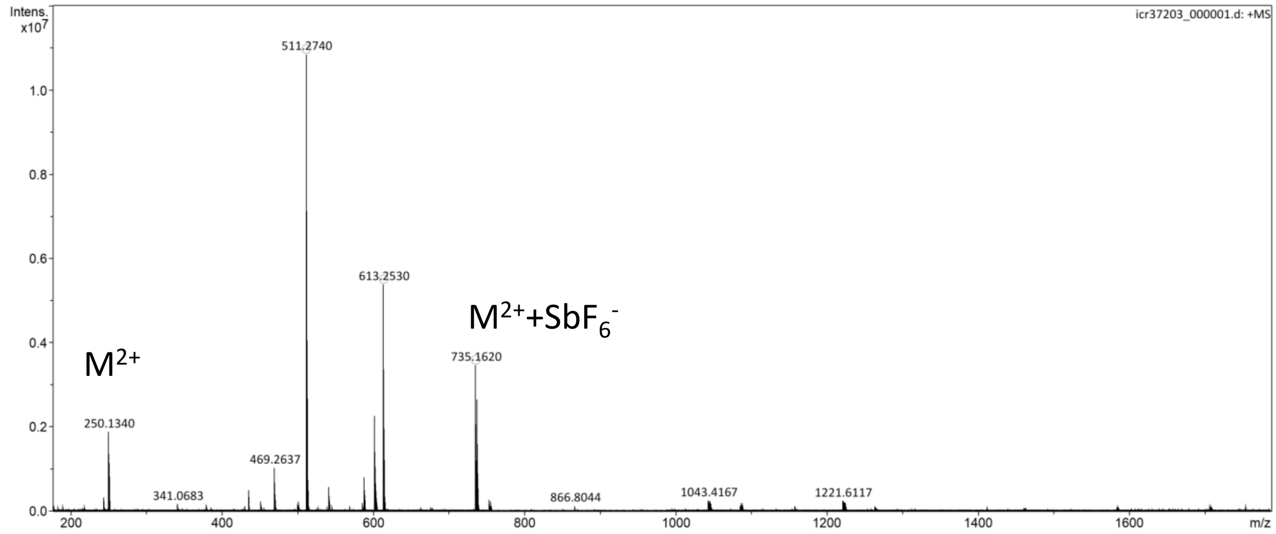

14. IR spectra

IR (KBr) **5**(BF_4)$_2$

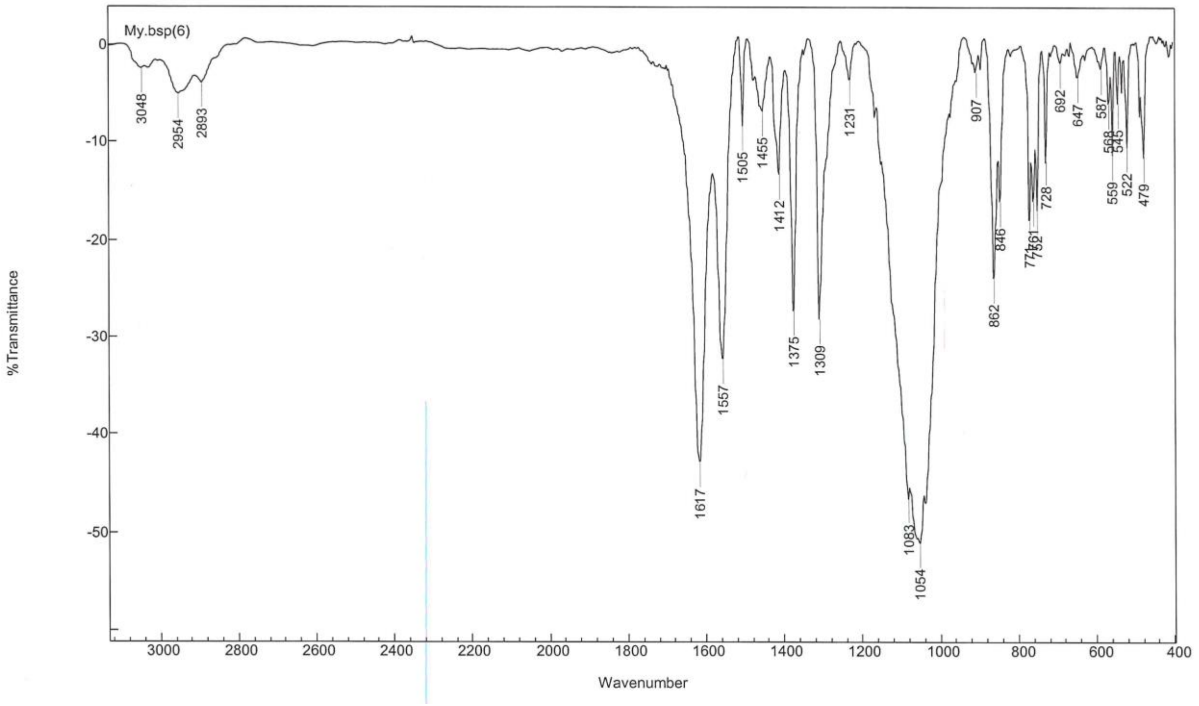

IR (KBr) **3**(BF_4)$_2$

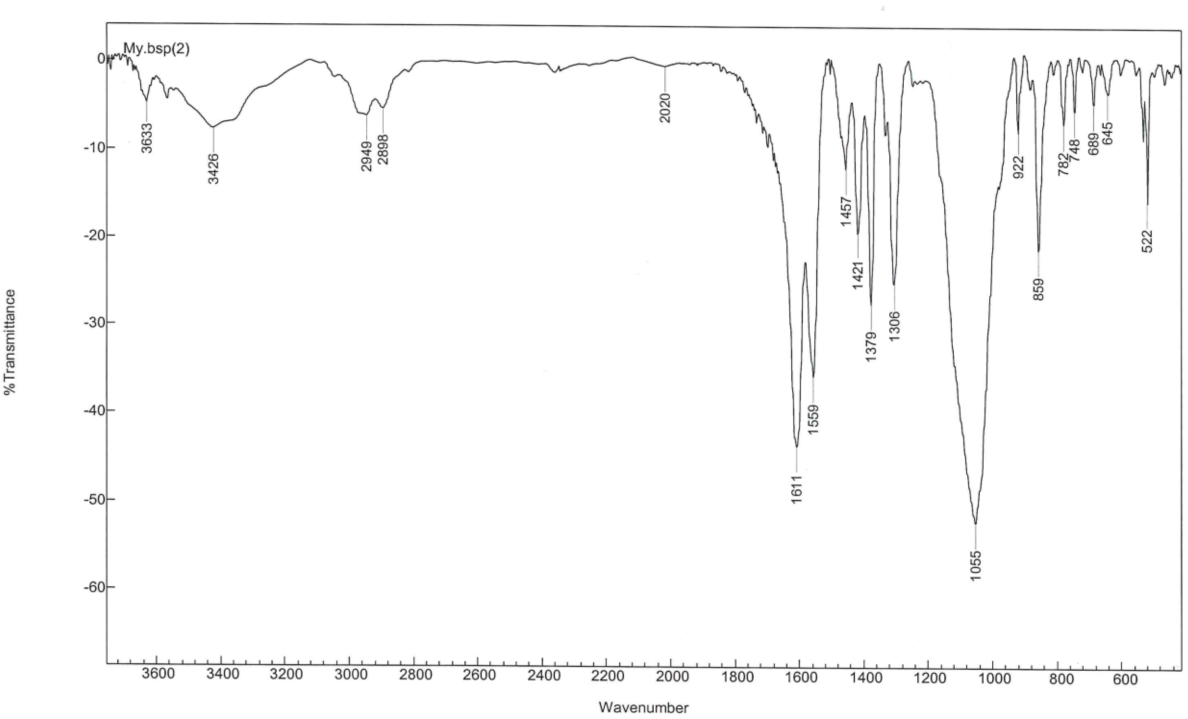

IR (KBr) **4**$(BF_4)_2$

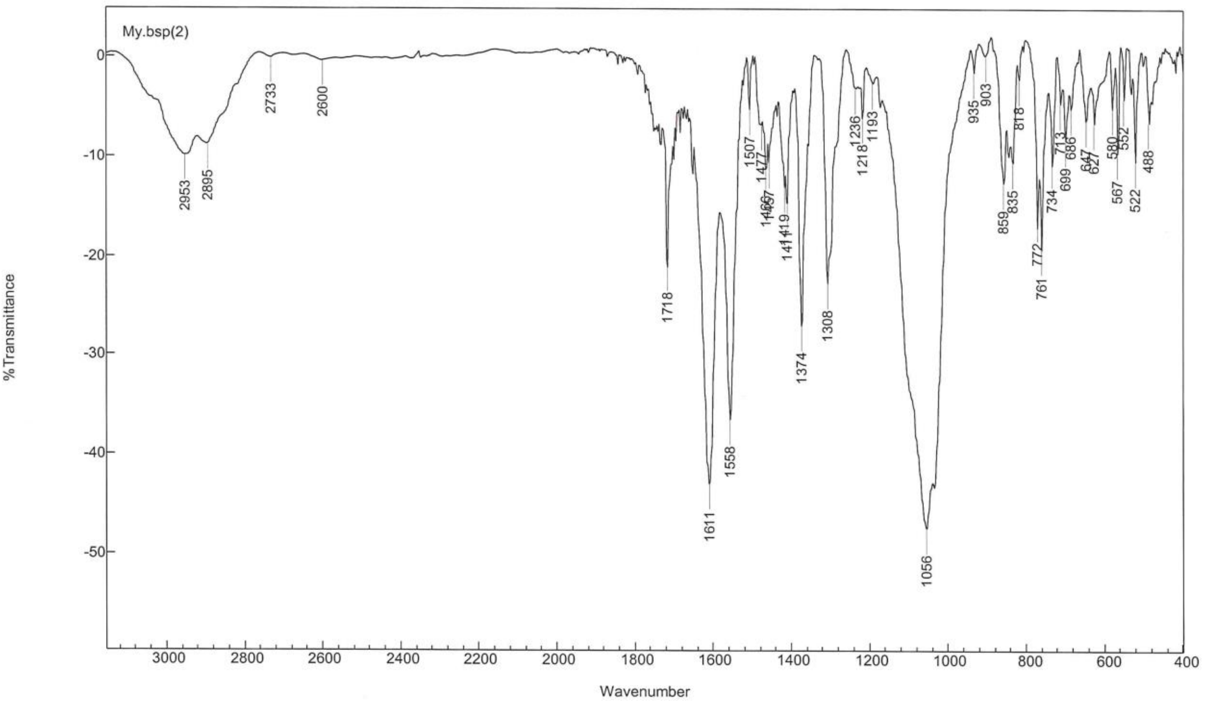

IR (KBr) **7**

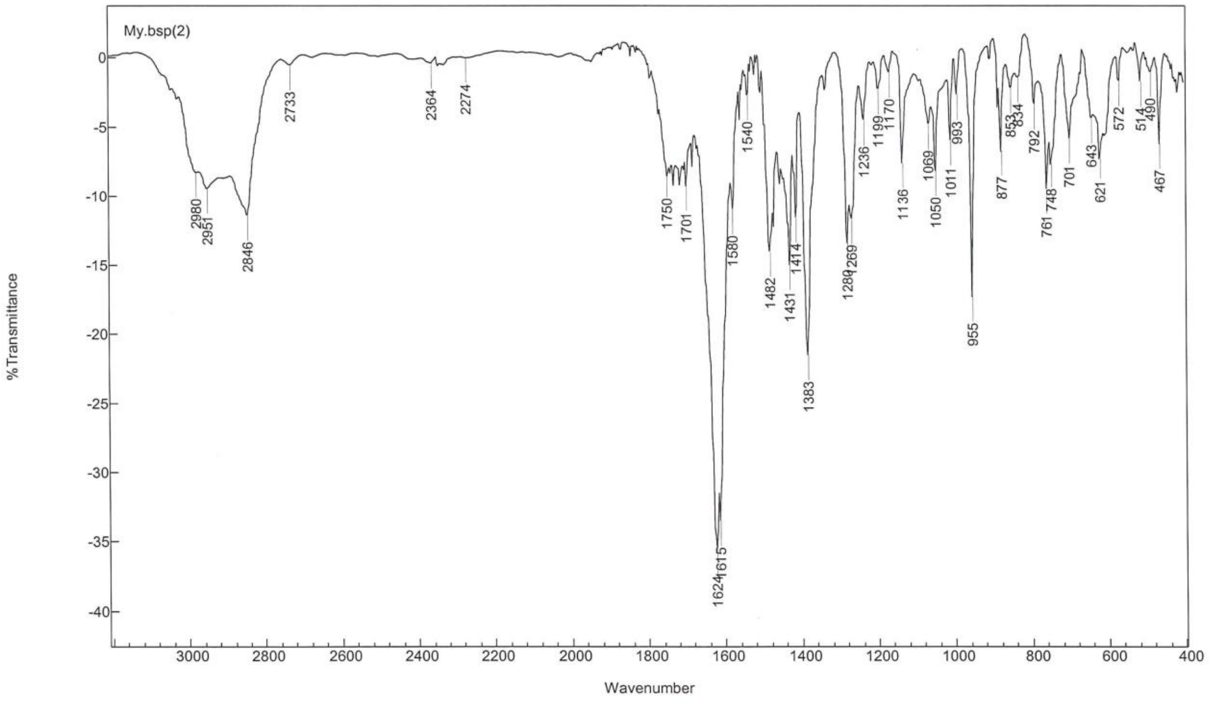

IR (KBr) **8**$(SbF_6)_2$

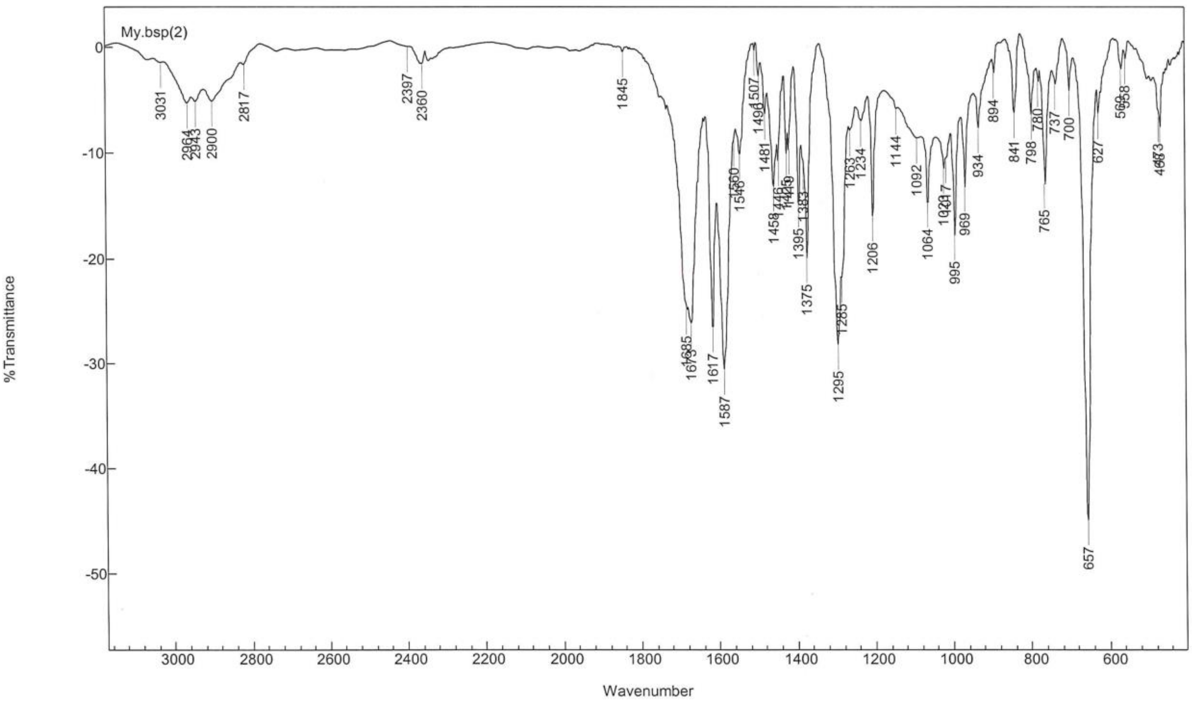

15. Calculated (B3LYP+D3/def2-TZVP) minimum structures (xyz coordinates)

Optimised structure of **1**$^{2+}$

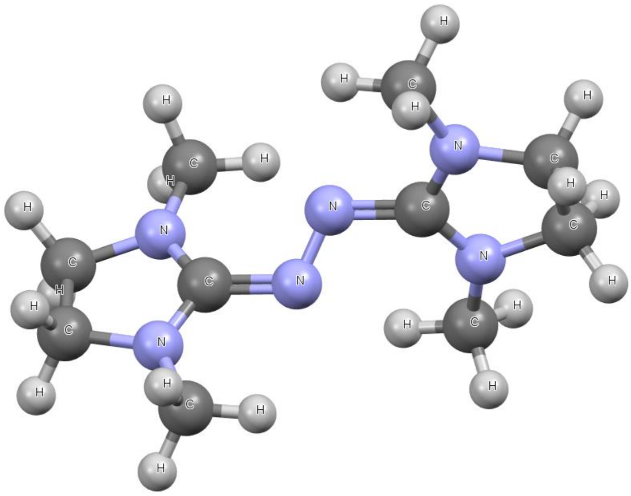

36
Energy = -720.8569747584
Coordinates

N	-2.1463346	-0.1859449	1.8484678
C	-2.4026255	-0.9787323	3.0690570
C	-0.9852391	-1.3997082	3.5039028
N	-0.1525501	-1.0404229	2.3281423
C	-0.8876042	-0.3397659	1.4831817
N	-0.4488783	0.3183553	0.3049035
N	0.3541004	-0.3240197	-0.3860090
C	0.8138162	0.3444768	-1.5502135
N	0.9883171	-0.3674501	-2.6475318
C	1.4304892	0.5211595	-3.7424561
C	1.7559094	1.8333996	-3.0030593
N	1.1779556	1.6102425	-1.6535506
C	1.2718561	2.6578347	-0.6408813
C	0.6154541	-1.7577423	-2.8926668
C	-3.2300233	0.4596122	1.1129562
C	1.2828764	-1.3071295	2.3440585
H	-3.0408488	-1.8277614	2.8175686
H	-2.9138889	-0.3635997	3.8083335
H	-0.8976506	-2.4661442	3.7053464
H	-0.6243935	-0.8457603	4.3723005
H	2.2928137	0.0863507	-4.2459557
H	0.6199073	0.6294458	-4.4655464
H	1.2972075	2.7085908	-3.4603268
H	2.8282464	2.0095500	-2.9010423
H	1.0266789	2.2737074	0.3433022
H	2.2901741	3.0483176	-0.6346613
H	0.5808010	3.4647256	-0.8867922
H	0.4525833	-2.2719341	-1.9492659
H	-0.2928868	-1.8002237	-3.4969755
H	1.4266282	-2.2429718	-3.4339813
H	-3.8084294	1.0649180	1.8098342
H	-2.8231344	1.0968871	0.3324624
H	-3.8820393	-0.2955052	0.6694028
H	1.6831706	-1.0009735	3.3110773

H	1.4585066	-2.3744221	2.2056353
H	1.7908446	-0.7617619	1.5559731

Optimised structure of **1**$(BF_4)_2$

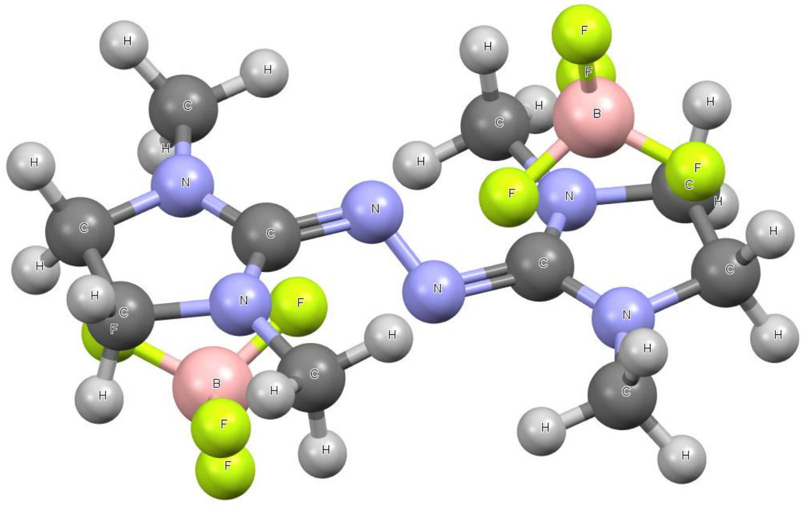

46
Energy = -1570.411159623
Coordinates

F	-0.2424577	-1.1482498	9.2035895
F	-1.1764561	-1.9375864	11.1601861
F	-0.1384460	0.1235230	11.1185054
F	1.1196992	-1.7869951	10.9418862
B	-0.1345947	-1.2141544	10.6313702
F	0.2729458	3.9963930	7.1427424
F	1.2126528	4.7789364	5.1861772
F	0.1711287	2.7197191	5.2308804
F	-1.0842675	4.6327141	5.3995209
B	0.1683868	4.0587458	5.7146788
N	0.6014249	1.2741244	8.0628228
N	1.3892401	2.2698018	10.1523216
N	2.3892876	0.4706041	9.3399670
C	1.4245866	1.3557183	9.2037886
C	2.3201377	1.8819542	11.2260751
H	1.7382684	1.6359773	12.1142851
H	2.9979872	2.7080421	11.4424972
C	3.0340328	0.6399935	10.6495806
H	4.1061650	0.7879312	10.5091561
H	2.8531497	-0.2549999	11.2428080
C	0.4227387	3.3368350	10.3496147
H	0.1439724	3.7687387	9.3921427
H	0.9035542	4.1068842	10.9517772
H	-0.4538619	2.9531281	10.8743068
C	2.6755312	-0.6722835	8.4900787
H	2.2332432	-1.5668094	8.9264446
H	3.7584957	-0.7787299	8.4191584
H	2.2650859	-0.4982853	7.5000099
N	-0.5773115	1.5721461	8.2797193
N	-1.3640913	0.5759176	6.1901788
N	-2.3619882	2.3779527	6.9989292
C	-1.3994256	1.4909682	7.1378040
C	-2.2914264	0.9652266	5.1138879
H	-1.7067484	1.2099433	4.2271582
H	-2.9702724	0.1404275	4.8957424
C	-3.0045170	2.2086575	5.6882040
H	-4.0771642	2.0625236	5.8266285
H	-2.8210749	3.1030269	5.0947968
C	-0.3987583	-0.4928174	5.9957557

H	-0.1252476	-0.9268088	6.9538632
H	-0.8785653	-1.2609663	5.3903721
H	0.4809140	-0.1103566	5.4753845
C	-2.6482286	3.5222063	7.8469898
H	-2.2090766	4.4164053	7.4067791
H	-3.7312349	3.6265766	7.9210310
H	-2.2343233	3.3517421	8.8361622

Optimised structure of the 2,3-dimethylbutadiene DA adduct

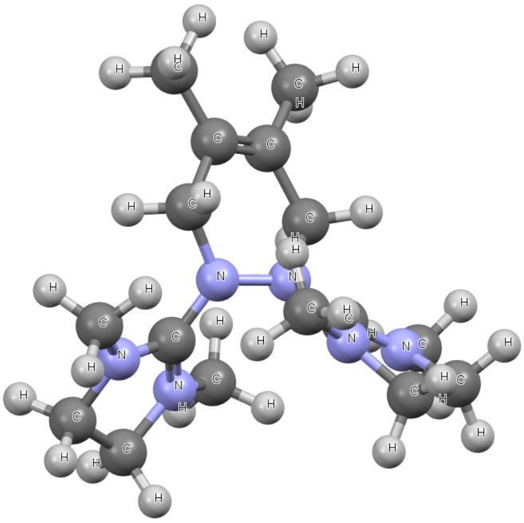

52

Energy = -955.4949393961

Coordinates

C	0.2880279	-0.9873447	1.5910974
N	-0.3518708	0.0646972	0.9941620
N	-0.4210313	0.1364718	-0.4011266
C	0.6875736	0.4917527	-1.1204891
C	-1.2440322	0.9679091	1.7498326
C	-1.7719743	0.1285953	-0.9993233
C	-2.5698205	1.2204057	1.0818749
C	-2.8158401	0.8235392	-0.1656253
C	-3.5216615	1.9556024	1.9825572
C	-4.1114714	0.9867729	-0.9089512
N	1.7004007	1.2453217	-0.6793756
N	0.8941074	0.0830119	-2.3775193
N	0.8672440	-0.8921383	2.7933647
N	0.4469285	-2.2077050	1.0678145
C	2.0769982	0.7635540	-2.9393040
C	2.7887553	1.2583885	-1.6797922
C	1.3180871	-2.2242567	3.2407946
C	1.3402323	-3.0106455	1.9297317
C	0.1266199	-0.8288861	-3.2169657
C	1.7545149	2.1049896	0.4927355
C	-0.2434618	-2.8136330	-0.0599406
C	1.0415115	0.2561249	3.6745297
H	-0.7478195	1.9231326	1.9438226
H	-1.4266383	0.4983088	2.7167199
H	-2.0945366	-0.8962314	-1.2045334
H	-1.6974979	0.6403053	-1.9591929
H	-4.4016282	2.3177859	1.4596854
H	-3.8552239	1.3130614	2.8029617
H	-3.0313865	2.8222612	2.4353765
H	-4.0085641	1.7220097	-1.7126949
H	-4.4087801	0.0432992	-1.3759269
H	-4.9284403	1.3020598	-0.2670670
H	1.7624509	1.5758947	-3.5988369
H	2.6732211	0.0572114	-3.5131831
H	3.1826184	2.2664084	-1.7872049
H	3.5975726	0.5964264	-1.3627048
H	0.6142080	-2.6312005	3.9707104
H	2.2989440	-2.1469275	3.7052381
H	0.9460945	-4.0186218	2.0365133

H	2.3370038	-3.0675486	1.4869126
H	0.8234476	-1.4714860	-3.7542989
H	-0.5258108	-1.4546778	-2.6209827
H	-0.4696475	-0.2774771	-3.9468731
H	2.3854010	2.9598131	0.2571898
H	0.7664901	2.4838287	0.7374538
H	2.1873054	1.5880582	1.3490303
H	-0.3489239	-3.8782654	0.1395130
H	-1.2416708	-2.3994128	-0.1671904
H	0.3198851	-2.6946998	-0.9853018
H	0.3223895	0.2294304	4.4958240
H	2.0466526	0.2193060	4.0934944
H	0.9309666	1.1867502	3.1320638

Optimised structure of the 2,3-dimethylbutadiene DA adduct including anions

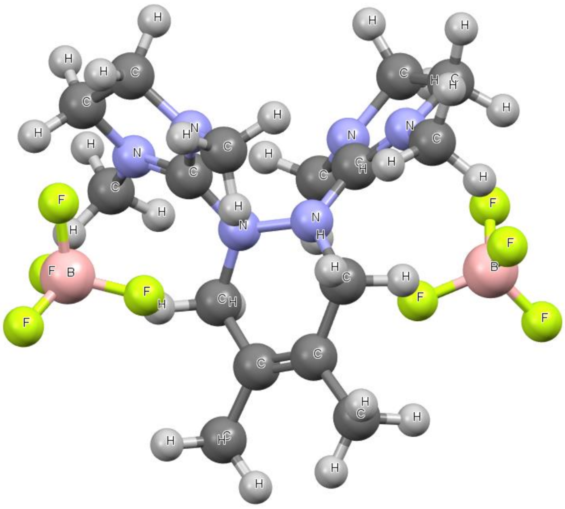

62

Energy = -1805.036093096

Coordinates

F	12.9665556	7.3393433	4.2464863
F	11.5267196	5.5512583	4.0001593
F	11.1658842	7.4868041	2.8087618
F	10.8156101	7.4532530	5.0745249
B	11.6602969	6.9642147	4.0427308
F	4.4258908	8.6980106	7.4516292
F	5.3380085	9.8655549	5.7015407
F	3.2402361	8.9517069	5.4956750
F	3.5730791	10.7668041	6.8834847
B	4.1240736	9.6076965	6.3924370
N	7.9230211	7.6253530	4.3149326
N	8.6013893	5.3289883	4.3332528
N	8.5513953	6.3897769	2.3932420
N	7.1462065	7.4894529	5.4664249
N	5.2995882	6.2420521	6.2720147
N	5.1373317	7.0768800	4.2305033
C	8.4647662	8.9556184	4.0010928
H	7.7720603	9.5040024	3.3542198
H	9.3971936	8.7886819	3.4642450
C	8.7534550	9.7966605	5.2115260
C	8.3454737	9.4512902	6.4264508
C	7.5945007	8.1767363	6.6864465
H	8.2306219	7.5142402	7.2825080
H	6.6956789	8.4131384	7.2536496
C	9.5241733	11.0328625	4.8512557
H	9.6025163	11.7375881	5.6742914
H	9.0462665	11.5551118	4.0161316
H	10.5346756	10.7625582	4.5300318
C	8.5636295	10.2291856	7.6911566
H	9.2470219	11.0637772	7.5621143
H	8.9767348	9.5844888	8.4736186
H	7.6093886	10.6174395	8.0599388
C	8.3742820	6.4929854	3.7139598
C	8.9681734	4.3036765	3.3417587
H	8.1487800	3.5902535	3.2188643
H	9.8629495	3.7857595	3.6747420
C	9.2326503	5.1276598	2.0795783
H	10.2952105	5.3171253	1.9277364
H	8.7918864	4.6878817	1.1854940

C	8.9649854	5.1584334	5.7319024
H	9.2715121	6.1077532	6.1549098
H	9.8358330	4.5074995	5.7727304
H	8.1468226	4.7130552	6.2995783
C	8.4520698	7.4138391	1.3720547
H	7.7169761	8.1617848	1.6461117
H	8.1286510	6.9389616	0.4455241
H	9.4238511	7.8861586	1.2182649
C	5.8914921	6.9849055	5.3316972
C	3.8907714	6.0246775	5.9203916
H	3.2667859	6.7270726	6.4729219
H	3.6103849	4.9984680	6.1554669
C	3.8922369	6.3130492	4.4175217
H	3.9149606	5.4035863	3.8109613
H	3.0458449	6.9294458	4.1286935
C	5.7501844	5.9343482	7.6150631
H	6.8324736	5.8925662	7.6586057
H	5.3582966	4.9544282	7.8883079
H	5.3781842	6.6829385	8.3166929
C	5.2077678	8.1296906	3.2284457
H	5.7762315	8.9693006	3.6108070
H	4.1971849	8.4942685	3.0568872
H	5.6267406	7.7556187	2.2933255

Optimised structure of the furan DA adduct

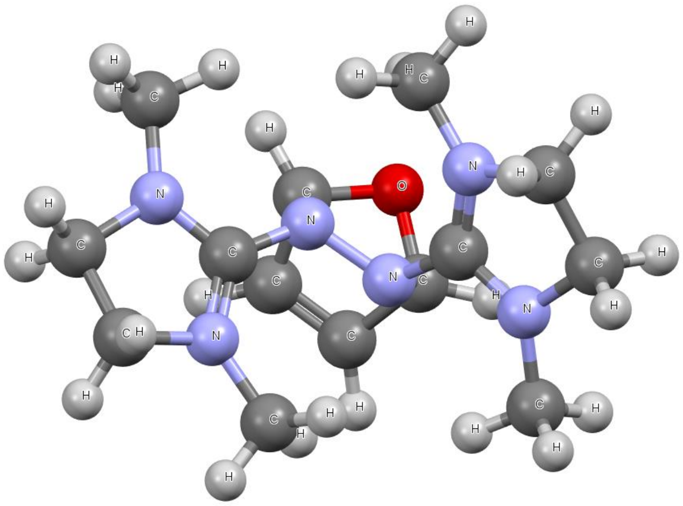

45

Energy = -950.8586214174

Coordinates

C	0.5605697	0.2521804	-2.8969129
C	1.0486577	-0.9757012	-3.0514830
C	2.0525663	-1.1385726	-1.9348919
N	1.2555074	-1.2975845	-0.6289944
N	0.6875545	0.0022414	-0.4580669
C	1.2874434	0.7983749	-1.6775751
O	2.5377448	0.1852435	-1.7295418
C	-0.6668600	0.0924668	-0.2098958
N	-1.5894476	-0.8662141	-0.2559338
N	-1.1761777	1.2630430	0.1754655
C	-2.6441527	1.1701814	0.2709705
C	-2.8685505	-0.3456469	0.2807918
C	1.9971463	-1.7867034	0.4452046
N	2.4911205	-1.1251395	1.4837619
N	2.2450746	-3.0922211	0.5041042
C	3.0509839	-2.0898312	2.4601982
C	3.1297978	-3.3887761	1.6455541
C	-1.4768136	-2.2659853	-0.6574567
C	-0.4790098	2.5191622	0.4135151
C	1.8829537	-4.1235808	-0.4573668
C	2.5748281	0.3138959	1.7242434
H	-0.2173346	0.7587117	-3.4473206
H	0.7789946	-1.7401658	-3.7632628
H	2.8482075	-1.8700538	-2.0113034
H	1.3418325	1.8608720	-1.4807018
H	-2.9908149	1.6567733	1.1805850
H	-3.1056969	1.6620240	-0.5883503
H	-3.6913029	-0.6539205	-0.3603453
H	-3.0339033	-0.7430227	1.2847401
H	2.3834103	-2.1690537	3.3204568
H	4.0251155	-1.7464561	2.8010546
H	2.7585773	-4.2550799	2.1899812
H	4.1380846	-3.6055776	1.2853979
H	-0.9875196	-2.8631699	0.1125779
H	-2.4858491	-2.6459925	-0.7992716
H	-0.9428117	-2.3572567	-1.5972348
H	-0.9470872	3.0197266	1.2599505
H	0.5623196	2.3293603	0.6592972
H	-0.5415583	3.1795589	-0.4545891

H	2.7505563	-4.4153042	-1.0536952
H	1.5222784	-4.9996652	0.0809598
H	1.0961494	-3.7635667	-1.1136766
H	1.6588142	0.6920425	2.1787290
H	3.3986631	0.4824375	2.4145697
H	2.7908083	0.8419351	0.8013816

Optimised structure of the pyrrol DA adduct

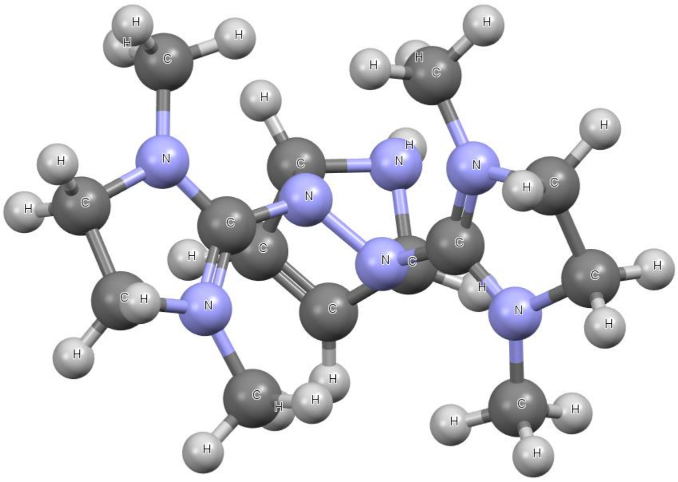

46
Energy = -930.9885266317
<u>Coordinates</u>

C	-2.6916192	1.4020104	-0.5578612
C	-3.0601005	0.3095602	0.1093226
C	-2.1358423	0.2512168	1.3282621
N	-1.8520386	1.6437777	1.6485431
C	-1.5604819	2.0116809	0.2517674
N	-0.3520837	1.0897029	0.0635434
N	-0.7476186	-0.0995988	0.7584468
C	0.8713799	1.6384432	0.4370199
N	1.4531314	2.5115211	-0.3827696
N	1.6069072	1.3810378	1.5109288
C	2.6767157	3.0503297	0.2368291
C	2.9180509	2.0594542	1.3837703
C	0.9513710	3.0347372	-1.6444041
C	1.2729086	0.6231117	2.7146310
C	-0.5715466	-1.3096078	0.1216393
N	-0.6746823	-2.4383415	0.8247625
N	-0.2461811	-1.5384457	-1.1492501
C	-0.5615450	-3.6040664	-0.0700101
C	0.0414370	-2.9797460	-1.3326621
C	-0.9234815	-2.5994279	2.2501949
C	-0.1021229	-0.6113733	-2.2686861
H	-3.0426597	1.7646385	-1.5121560
H	-3.7850906	-0.4415926	-0.1676035
H	-2.3981413	-0.3773029	2.1697890
H	-1.2895504	3.0479135	0.0861260
H	2.4981662	4.0701720	0.5854720
H	3.4873455	3.0654245	-0.4892524
H	3.1710273	2.5520009	2.3199177
H	3.6910248	1.3237536	1.1522922
H	1.7689851	3.0694897	-2.3638839
H	0.1679945	2.3896665	-2.0315183
H	0.5597338	4.0464658	-1.5153183
H	1.4938214	-0.4370649	2.5859533
H	1.8891574	1.0075824	3.5247588
H	0.2302654	0.7727366	2.9739311
H	-1.5482054	-4.0404923	-0.2416811
H	0.0814534	-4.3584309	0.3791336
H	-0.4295150	-3.3375418	-2.2453907
H	1.1203425	-3.1332360	-1.4077845

H	-0.3298660	-3.4376080	2.6126544
H	-0.6211009	-1.7049134	2.7874745
H	-1.9767294	-2.8121768	2.4475761
H	-0.1462307	-1.1966126	-3.1844017
H	-0.9149285	0.1061202	-2.2824776
H	0.8565749	-0.0927313	-2.2349632
H	-2.6858919	2.1124926	1.9930546

Optimised structure of the cyclopentadiene DA adduct

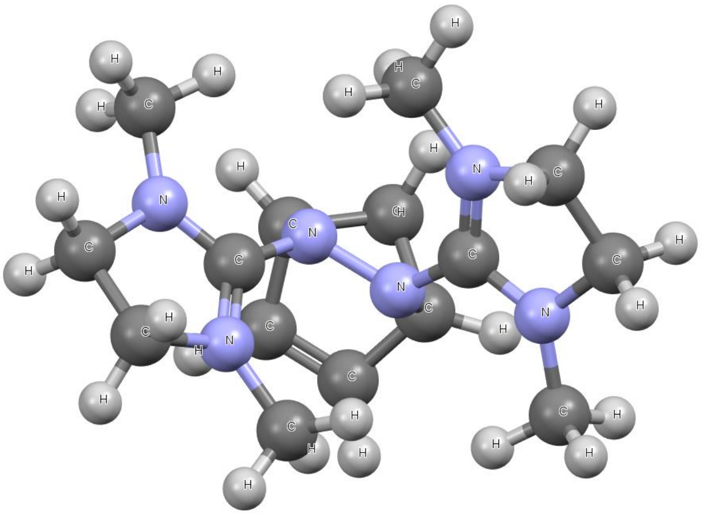

47

Energy = -914.9552903468

Coordinates

C	-2.5572164	1.0523063	-1.2540486
C	-2.9846626	-0.0397038	-0.6166259
C	-2.2924353	-0.0538689	0.7373757
C	-2.2168425	1.4397531	1.0556163
C	-1.5991087	1.7560124	-0.3199855
N	-0.3491698	0.8635313	-0.2309615
N	-0.7995325	-0.3165124	0.4437033
C	0.8408818	1.4230933	0.1696331
N	1.4324217	2.3341182	-0.6060961
N	1.5691434	1.1414895	1.2517494
C	2.6252373	2.8767532	0.0667106
C	2.8749593	1.8346560	1.1619460
C	0.9927469	2.8466163	-1.8954876
C	1.2748324	0.2695400	2.3850802
C	-0.4388981	-1.5340923	-0.0947921
N	-0.5654049	-2.6390627	0.6418492
N	0.0980481	-1.7874953	-1.2877870
C	-0.2355468	-3.8268377	-0.1655870
C	0.5108089	-3.2082495	-1.3522444
C	-1.0361897	-2.7656100	2.0133318
C	0.3264287	-0.9002498	-2.4252255
H	-2.7908409	1.3722125	-2.2587763
H	-3.6433363	-0.8123465	-0.9857879
H	-2.6545246	-0.7294870	1.5012384
H	-3.2017652	1.8801534	1.1823045
H	-1.5787573	1.6934468	1.9019480
H	-1.3177685	2.7778875	-0.5405510
H	2.4093305	3.8698314	0.4676503
H	3.4493083	2.9560799	-0.6396815
H	3.1228808	2.2831970	2.1212939
H	3.6552087	1.1183755	0.8944579
H	1.8606833	2.9431817	-2.5470256
H	0.2883130	2.1574658	-2.3528007
H	0.5274081	3.8294435	-1.7920700
H	1.9436006	0.5496379	3.1958282
H	0.2536099	0.4019663	2.7271664
H	1.4500610	-0.7788331	2.1398526
H	-1.1517043	-4.3427079	-0.4624822
H	0.3803843	-4.5127453	0.4126974

H	0.2135799	-3.6373927	-2.3064274
H	1.5964141	-3.2819326	-1.2550235
H	-0.4450606	-3.5316017	2.5136716
H	-0.9060338	-1.8261922	2.5431247
H	-2.0865218	-3.0639866	2.0453795
H	0.4443530	-1.5242210	-3.3083001
H	-0.5246123	-0.2467656	-2.5805694
H	1.2350884	-0.3108538	-2.2951721

Optimised structure of the anthracene DA adduct

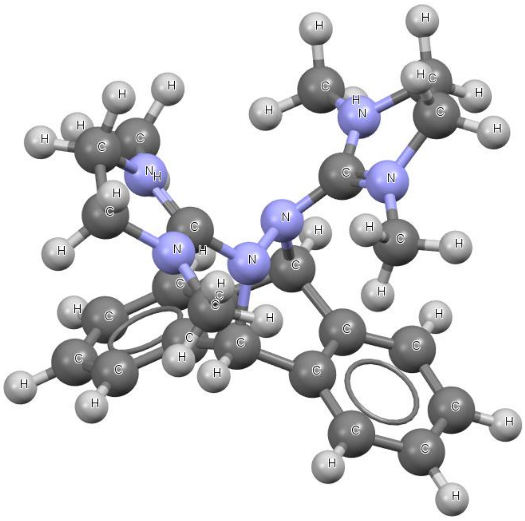

60
Energy = -1260.305320051
Coordinates

C	0.0051251	1.3794382	1.9557230
C	-0.7945149	0.2662796	2.2340507
C	-0.3549201	-0.9573098	1.4799269
C	1.1107635	-1.2174425	1.7359018
C	1.9035878	-0.1052359	1.4348972
C	1.0906226	1.0639760	0.9539496
C	1.6853758	-2.3959124	2.1778767
C	3.0720644	-2.4463375	2.3401521
C	3.8600409	-1.3380948	2.0484651
C	3.2787041	-0.1561413	1.5840480
C	-0.2582407	2.6034728	2.5444799
C	-1.3251508	2.7007543	3.4411896
C	-2.1159679	1.5922268	3.7239864
C	-1.8593027	0.3627593	3.1130807
N	0.3374223	0.5753181	-0.3079597
N	-0.4372028	-0.5901216	-0.0225068
C	-0.2751159	1.5528446	-1.0660563
N	-1.5749021	1.7661985	-1.2614766
N	0.4774124	2.4146277	-1.7556658
C	-1.7591714	2.7972852	-2.3052030
C	-0.3695756	3.4381624	-2.3892600
C	-2.7311997	1.2073163	-0.5731257
C	1.9256397	2.4691787	-1.8659445
C	-0.3223945	-1.6400856	-0.9112917
N	-1.3093734	-2.5361099	-0.9978583
N	0.6574612	-1.8999084	-1.7746628
C	0.2593971	-3.0159825	-2.6591834
C	-0.9293830	-3.6273489	-1.9095751
C	-2.5759484	-2.5635187	-0.2854836
C	1.9873814	-1.3153934	-1.8876791
H	-0.9868405	-1.8226998	1.6491177
H	1.6894089	1.9246065	0.6753740
H	1.0742597	-3.2578790	2.4169620
H	3.5355991	-3.3530241	2.7060387
H	4.9315752	-1.3886926	2.1893227
H	3.8950246	0.7065211	1.3613779
H	0.3617849	3.4668878	2.3352808
H	-1.5310681	3.6450279	3.9277730
H	-2.9322482	1.6802387	4.4286240

H	-2.4751524	-0.4996531	3.3381296
H	-2.0656350	2.3263748	-3.2420248
H	-2.5304696	3.5003175	-1.9987397
H	-0.2998715	4.3773759	-1.8352591
H	-0.0436985	3.6116397	-3.4131290
H	-3.1729142	0.3941649	-1.1515044
H	-2.4604068	0.8660847	0.4181854
H	-3.4722625	1.9983984	-0.4677821
H	2.3297146	3.2913359	-1.2702870
H	2.3631950	1.5333798	-1.5336359
H	2.1954212	2.6350213	-2.9089454
H	1.0883864	-3.7107536	-2.7740572
H	-0.0139116	-2.6299088	-3.6438438
H	-0.6587761	-4.5172766	-1.3362689
H	-1.7625785	-3.8741747	-2.5649157
H	-3.3728120	-2.8052689	-0.9888605
H	-2.5644564	-3.3253271	0.4977558
H	-2.7791051	-1.5936933	0.1569628
H	2.0151246	-0.5607605	-2.6754457
H	2.3021316	-0.8908699	-0.9426088
H	2.6819567	-2.1125982	-2.1484913

SUPPORTING INFORMATION

Optimised structure of the tetracene DA adduct

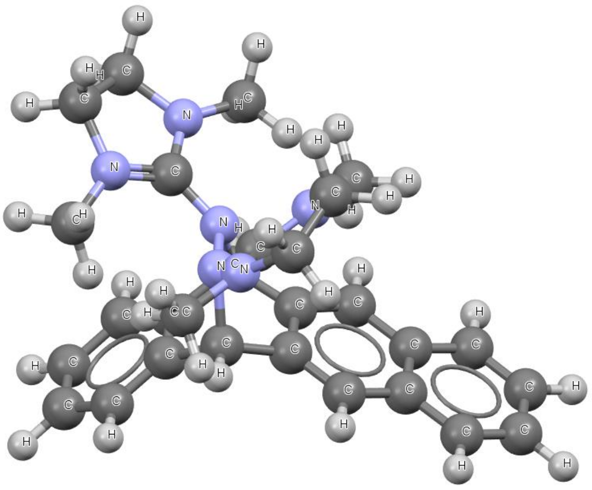

66

Energy = -1413.917524685

Coordinates

C	-3.3834309	-2.2591513	1.4794375
C	-2.9418978	-2.9759931	0.3720645
C	-1.7206876	-2.6697716	-0.2320723
C	-0.9521219	-1.6490635	0.3000298
C	-1.3989626	-0.9233328	1.4094915
C	-2.6142243	-1.2186972	2.0036361
C	0.3811063	-1.1544551	-0.2062714
C	-0.4293139	0.1453307	1.8269236
C	1.3862861	-1.1728658	0.9082100
C	0.9334642	-0.4592080	2.0491945
C	2.6101768	-1.7788184	0.9002427
C	3.4433527	-1.7049105	2.0490498
C	2.9898140	-0.9770952	3.1973260
C	1.7147424	-0.3488783	3.1630146
C	4.7080854	-2.3340726	2.0923059
C	5.4912150	-2.2502797	3.2166301
C	5.0447482	-1.5347511	4.3465466
C	3.8213165	-0.9117764	4.3378207
N	-0.2587828	1.0776058	0.5977590
N	0.1441372	0.3369889	-0.5556394
C	-0.5481375	0.5883016	-1.7213222
C	0.4060630	2.2637447	0.8301407
N	1.6004082	2.6536582	0.3890252
C	1.8190806	4.0748746	0.7315897
C	0.7434750	4.3314065	1.7926753
N	-0.1837827	3.2121384	1.5638817
N	0.0287918	0.3240141	-2.8972651
C	-0.9242303	0.5489184	-3.9956486
C	-2.0105225	1.3831063	-3.3079688
N	-1.7608367	1.1173317	-1.8754501
C	1.3651728	-0.1839286	-3.1574957
C	-2.8056009	1.3672576	-0.8912979
C	-1.5083425	3.2038868	2.1612046
C	2.6557281	1.8876792	-0.2609296
H	-4.3271736	-2.5141706	1.9430603
H	-3.5451889	-3.7846056	-0.0191353
H	-1.3750433	-3.2377654	-1.0874188
H	-2.9586423	-0.6653074	2.8689746
H	0.7197370	-1.6596652	-1.1047333

H	-0.7792919	0.7481044	2.6583208
H	2.9549559	-2.3299989	0.0323057
H	1.3695072	0.1938994	4.0361188
H	5.0501138	-2.8909972	1.2283950
H	6.4560839	-2.7395468	3.2420188
H	5.6718810	-1.4831684	5.2269745
H	3.4778735	-0.3703614	5.2109632
H	2.8287086	4.2123588	1.1122727
H	1.6921486	4.6954143	-0.1584565
H	0.2273204	5.2794262	1.6530153
H	1.1342233	4.2919154	2.8123784
H	-0.4361016	1.0783407	-4.8117639
H	-1.2962983	-0.4074273	-4.3710855
H	-3.0168049	1.0667784	-3.5733221
H	-1.9141824	2.4523933	-3.5098029
H	1.8086091	0.3869537	-3.9733988
H	1.9859593	-0.0764580	-2.2739201
H	1.3305503	-1.2358448	-3.4512467
H	-3.7639154	1.1375412	-1.3549411
H	-2.6848061	0.7213605	-0.0305749
H	-2.8108931	2.4150997	-0.5865059
H	-1.9828440	4.1692587	1.9848308
H	-2.1169841	2.4250662	1.7133333
H	-1.4470543	3.0414568	3.2400741
H	3.6110112	2.2386534	0.1268087
H	2.5651402	0.8338444	-0.0282524
H	2.6413913	2.0450772	-1.3407857

Optimised structure of the pentacene DA adduct

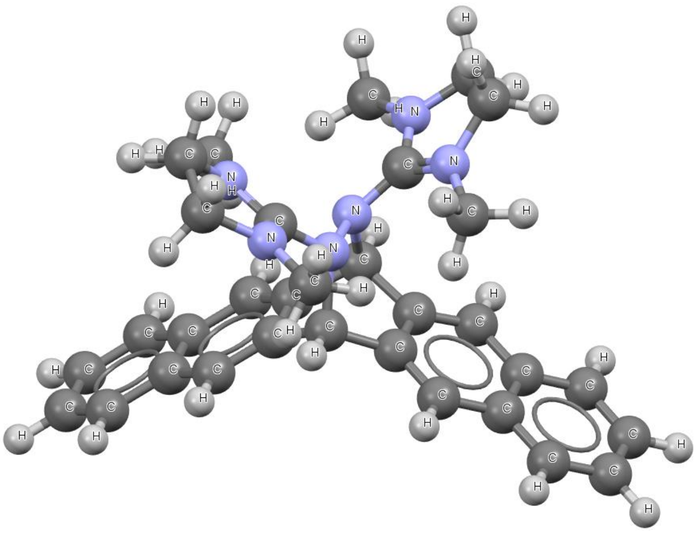

72

Energy = -1567.529256890

Coordinates

C	-0.0903705	1.2451652	2.3462751
C	-0.8416288	0.0555602	2.5405724
C	-0.3115767	-1.0907453	1.7305759
C	1.1555766	-1.2830881	2.0138525
C	1.8985882	-0.0935726	1.7895931
C	1.0273243	1.0481460	1.3554645
C	1.7766072	-2.4339799	2.4061803
C	3.1819336	-2.4428542	2.6186843
C	3.9309057	-1.2407409	2.4000293
C	3.2521321	-0.0682077	1.9725195
C	-0.4263538	2.3997698	2.9928795
C	-1.5265660	2.4126622	3.8926715
C	-2.2801195	1.2105170	4.0948117
C	-1.9123228	0.0338946	3.3887283
N	0.3246622	0.5889215	0.0483103
N	-0.3748512	-0.6416925	0.2446065
C	-0.3417436	1.5682416	-0.6558957
N	-1.6504961	1.7097006	-0.8589886
N	0.3596194	2.5234473	-1.2741842
C	-1.8898782	2.7947007	-1.8333735
C	-0.5451069	3.5295841	-1.8510515
C	-2.7754379	1.0363569	-0.2237226
C	1.8018187	2.6761505	-1.3594748
C	-0.1586184	-1.6271925	-0.6940825
N	-1.0752153	-2.5855539	-0.8613345
N	0.8637596	-1.7724279	-1.5353056
C	0.5751637	-2.8649999	-2.4875682
C	-0.5891606	-3.5974607	-1.8118177
C	-2.3588506	-2.7363525	-0.1978887
C	2.1540810	-1.0969061	-1.5673349
H	-0.8965844	-1.9988980	1.8308649
H	1.5798262	1.9558910	1.1371251
H	1.2101710	-3.3421610	2.5806922
H	3.8203549	0.8408140	1.8086727
H	0.1481597	3.3080836	2.8478385
H	-2.4828815	-0.8751801	3.5438734
H	-2.1529143	2.3704930	-2.8051642
H	-2.7097904	3.4232463	-1.4931616
H	-0.5456227	4.4315426	-1.2341061

H	-0.2194657	3.7937912	-2.8554839
H	-3.1654490	0.2430938	-0.8638686
H	-2.4901619	0.6398727	0.7428490
H	-3.5622085	1.7732991	-0.0680578
H	2.1469873	3.4803081	-0.7050357
H	2.2938074	1.7496588	-1.0817274
H	2.0719120	2.9270405	-2.3853542
H	1.4553034	-3.4926189	-2.6083203
H	0.3051710	-2.4485651	-3.4608180
H	-0.2722319	-4.4949736	-1.2750097
H	-1.3809371	-3.8681948	-2.5078409
H	-2.3200821	-3.5352127	0.5466529
H	-2.6423252	-1.8070401	0.2854500
H	-3.1139808	-2.9940665	-0.9406822
H	2.1618515	-0.3096137	-2.3230589
H	2.4004267	-0.6922497	-0.5935161
H	2.9117069	-1.8345108	-1.8285304
C	5.3272447	-1.2542068	2.6195766
C	5.9574632	-2.4001303	3.0360641
C	5.2203249	-3.5828275	3.2520288
C	3.8630378	-3.6043523	3.0480869
H	5.8945706	-0.3449672	2.4617714
H	7.0261656	-2.3991481	3.2060308
H	5.7316242	-4.4762477	3.5859323
H	3.2986505	-4.5122695	3.2232427
C	-3.3691148	1.2280447	4.9959180
C	-3.7008186	2.3779556	5.6678198
C	-2.9588820	3.5607286	5.4694166
C	-1.8955199	3.5783380	4.6015016
H	-3.9358127	0.3187863	5.1558473
H	-4.5332021	2.3800985	6.3592982
H	-3.2300131	4.4573587	6.0113177
H	-1.3224860	4.4863856	4.4580028

Optimised structure of **7**

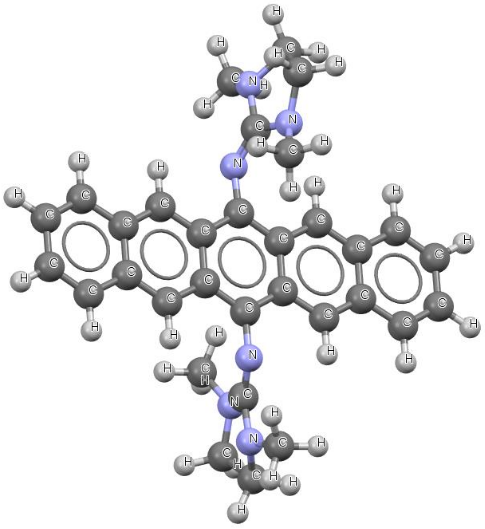

70
Energy = -1566.857582522
Coordinates

C	-6.6555647	-0.3835151	-0.7450214
C	-6.4476255	-1.6501634	-0.1173060
C	-5.1957695	-2.0456994	0.2415409
C	-4.0598516	-1.2092934	0.0015106
C	-4.2712722	0.0762546	-0.6276662
C	-5.6051083	0.4465546	-0.9899051
C	-2.7732314	-1.5874078	0.3482698
C	-1.6573337	-0.7595355	0.1166137
C	-1.8704247	0.5329952	-0.5049973
C	-3.1833909	0.9024784	-0.8569338
C	-0.3540036	-1.1773223	0.4688159
C	0.7380612	-0.3094193	0.2361800
C	0.5244612	0.9866630	-0.3876906
C	-0.7808795	1.4023128	-0.7388836
C	2.0523898	-0.6717349	0.5940706
C	3.1403810	0.1517169	0.3625367
C	2.9304638	1.4322232	-0.2765917
C	1.6449843	1.8080184	-0.6254137
C	4.4737999	-0.2164377	0.7294874
C	5.5249298	0.6109041	0.4798117
C	5.3183422	1.8733439	-0.1579136
C	4.0673356	2.2663447	-0.5214661
N	-0.9976784	2.6079505	-1.3974312
N	-0.1791405	-2.3909330	1.1254435
C	-0.9792977	3.7725708	-0.8748001
C	0.3070921	-3.4606980	0.6264524
N	0.4843246	-4.6025702	1.3987329
C	1.3290609	-5.5457743	0.6887662
N	-1.1548124	4.9142409	-1.6477798
C	-0.7780258	6.0867073	-0.8792484
C	1.0430848	-5.1728848	-0.7607466
N	0.7563623	-3.7459273	-0.6584225
C	-1.0444476	5.6113479	0.5437246
N	-0.7858844	4.1790191	0.4410976
C	-1.1603404	3.3625347	1.5824368
C	-0.8919910	4.8849340	-3.0687342
C	0.2221744	-3.0984666	-1.8437456
C	0.5845654	-4.5063066	2.8373830
H	-7.6597770	-0.0891517	-1.0245413

H	-7.2978471	-2.2944136	0.0704905
H	-5.0363755	-3.0065149	0.7178279
H	-5.7614887	1.4079835	-1.4659265
H	-2.6102797	-2.5448494	0.8257591
H	-3.3350666	1.8620313	-1.3338674
H	2.2130541	-1.6262305	1.0766272
H	1.4932166	2.7641209	-1.1076560
H	4.6293660	-1.1745855	1.2125061
H	6.5286661	0.3181799	0.7626578
H	6.1691035	2.5158497	-0.3490852
H	3.9092376	3.2239742	-1.0047081
H	2.3912222	-5.3944382	0.9348253
H	1.0642885	-6.5771490	0.9261971
H	0.2851127	6.3333175	-1.0223432
H	-1.3739027	6.9567220	-1.1589626
H	0.1702650	-5.7216338	-1.1429280
H	1.8866969	-5.3561200	-1.4275017
H	-2.0881247	5.8015518	0.8326906
H	-0.3902360	6.0756037	1.2828859
H	-0.7746941	3.8380581	2.4849733
H	-2.2491284	3.2629740	1.6752497
H	-0.7250365	2.3714823	1.5083001
H	-1.4013724	5.7228971	-3.5474059
H	0.1826560	4.9544801	-3.2889794
H	-1.2675966	3.9512054	-3.4799432
H	-0.8157417	-3.3953031	-2.0401582
H	0.2631178	-2.0186689	-1.7461331
H	0.8358662	-3.3874679	-2.6977454
H	-0.1390907	-3.7782417	3.1955391
H	0.3689409	-5.4800658	3.2802377
H	1.5863489	-4.1879328	3.1587410

Optimised structure of **7**$^{\cdot+}$ radical cation

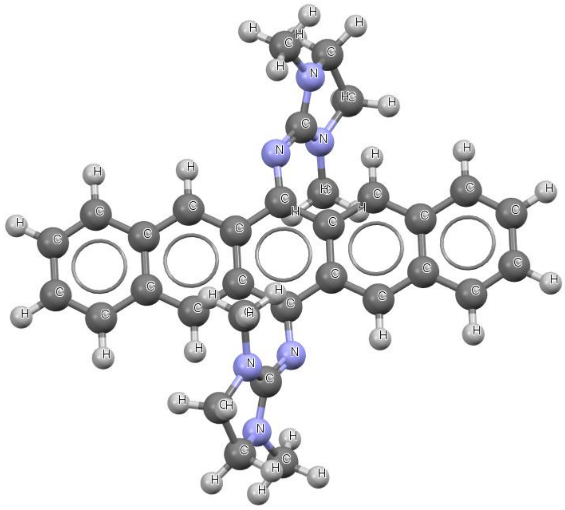

70
Energy = -1566.683326701
Coordinates

C	-6.4850639	0.4399319	-0.4399644
C	-6.4104509	-0.8468070	0.1561065
C	-5.1990378	-1.4061490	0.4509293
C	-3.9920963	-0.7083902	0.1702870
C	-4.0680260	0.5975292	-0.4222718
C	-5.3471657	1.1413734	-0.7234990
C	-2.7337669	-1.2575021	0.4269953
C	-1.5515993	-0.5692387	0.1607176
C	-1.6261478	0.7664653	-0.3779119
C	-2.8828908	1.2899345	-0.6826186
C	-0.2802850	-1.2112239	0.3759309
C	0.9250610	-0.4600126	0.1294204
C	0.8506011	0.8759250	-0.4085771
C	-0.4207786	1.5177043	-0.6246442
C	2.1817204	-0.9837994	0.4339592
C	3.3668754	-0.2908619	0.1751124
C	3.2910579	1.0157061	-0.4160395
C	2.0327837	1.5646456	-0.6734697
C	4.6459404	-0.8346618	0.4767306
C	5.7838358	-0.1323443	0.1953463
C	5.7093243	1.1552105	-0.3989559
C	4.4979854	1.7143903	-0.6943962
N	-0.4259987	2.7674529	-1.1106041
N	-0.2748914	-2.4612325	0.8615679
C	-1.1600688	3.7908210	-0.7862602
C	0.4565390	-3.4854274	0.5345599
N	0.8409994	-4.4298361	1.4409799
C	1.3305032	-5.6181303	0.7498963
N	-1.5463522	4.7323294	-1.6946116
C	-2.0406808	5.9203853	-1.0066042
C	1.7342084	-5.0429154	-0.6102935
N	0.8769213	-3.8642359	-0.7038878
C	-2.4422221	5.3471558	0.3551443
N	-1.5816562	4.1710741	0.4512566
C	-1.5166325	3.3815979	1.6606148
C	-0.9800134	4.8257034	-3.0238661
C	0.8141718	-3.0721870	-1.9116848
C	0.2725411	-4.5249411	2.7692596
H	-7.4548928	0.8620250	-0.6701424

H	-7.3244747	-1.3845749	0.3726571
H	-5.1410112	-2.3900149	0.9005125
H	-5.4047382	2.1223633	-1.1801883
H	-2.6651670	-2.2525758	0.8457616
H	-2.9593369	2.2578284	-1.1587268
H	2.2579050	-1.9522692	0.9089635
H	1.9642778	2.5600484	-1.0914853
H	4.7034719	-1.8162853	0.9320506
H	6.7535999	-0.5544038	0.4258569
H	6.6233428	1.6937349	-0.6136338
H	4.4399935	2.6989269	-1.1425192
H	2.1639647	-6.0690587	1.2868718
H	0.5321006	-6.3644541	0.6518082
H	-1.2452718	6.6701477	-0.9106547
H	-2.8759325	6.3665860	-1.5447667
H	1.5454770	-5.7280269	-1.4363015
H	2.7916014	-4.7531465	-0.6313923
H	-3.4987758	5.0544376	0.3773657
H	-2.2551465	6.0347412	1.1794731
H	-1.4375218	4.0550136	2.5143269
H	-2.3999389	2.7479464	1.7872037
H	-0.6327877	2.7478656	1.6428574
H	-1.7161396	5.2603756	-3.7003034
H	-0.0800706	5.4513527	-3.0321980
H	-0.7186149	3.8297218	-3.3724970
H	0.7335859	-3.7437310	-2.7667284
H	-0.0680449	-2.4362314	-1.8929510
H	1.6990917	-2.4404943	-2.0368422
H	-0.6341722	-5.1407987	2.7736631
H	1.0037423	-4.9714758	3.4432536
H	0.0217786	-3.5283230	3.1238513

Optimised structure of **8**

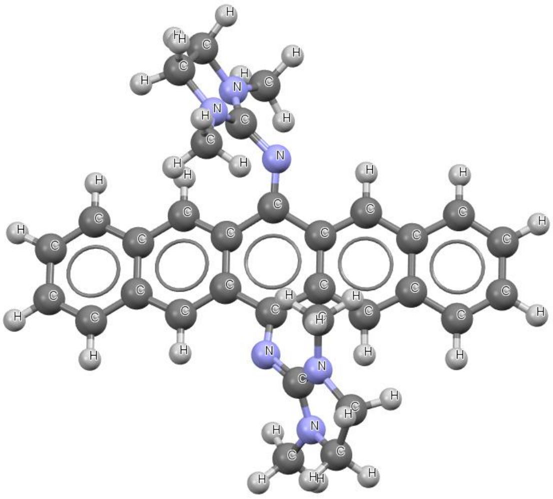

70
Energy = -1566.407692682
Coordinates

C	-4.1568806	-0.4686400	-0.0165610
C	-3.8355473	-1.7398237	0.5077677
C	-2.5256870	-2.1127714	0.6815170
C	-1.4805838	-1.2234915	0.3417760
C	-1.8062001	0.0674611	-0.1808791
C	-3.1647898	0.4173394	-0.3562488
C	-0.1226625	-1.5737305	0.4779200
C	0.8944914	-0.7110493	0.1263223
C	0.5715892	0.6028841	-0.3477441
C	-0.7558354	0.9503325	-0.5029561
C	2.2944340	-1.1515705	0.2178070
C	3.2942279	-0.3681336	-0.5200757
C	2.9882833	0.9781925	-0.9065219
C	1.6556060	1.5457231	-0.6535531
C	4.5272445	-0.8947537	-0.8506997
C	5.5080308	-0.1432108	-1.5287094
C	5.2180033	1.2159768	-1.8666775
C	3.9501323	1.7367916	-1.5404028
C	6.7638280	-0.6882713	-1.8820596
C	7.6953792	0.0836738	-2.5308432
C	7.4121618	1.4280822	-2.8570088
C	6.1987088	1.9839605	-2.5351897
N	1.5273536	2.8286145	-0.7527127
N	2.5384137	-2.2151963	0.9114867
C	0.5139798	3.6428385	-0.4353187
N	-0.1338448	4.3907750	-1.3378743
C	-0.9255495	5.4252929	-0.6570025
C	-0.9878804	4.8935813	0.7824835
N	0.1272368	3.9369862	0.8119391
C	0.4702101	3.2008182	2.0121899
C	0.2317102	4.5189361	-2.7390582
C	3.6907154	-2.7675496	1.3084574
N	4.0868125	-3.9884083	0.9260416
C	5.1803611	-4.4550316	1.7903732
C	5.6530849	-3.1514198	2.4507349
N	4.5036186	-2.2598359	2.2429190
C	4.4934791	-0.8986974	2.7404094
C	3.2945763	-4.9198786	0.1399061
H	-5.1956150	-0.1974111	-0.1527475

H	-4.6325171	-2.4238277	0.7684157
H	-2.2810334	-3.0906322	1.0770291
H	-3.4146953	1.3893198	-0.7640217
H	0.1306392	-2.5583334	0.8479294
H	-1.0234189	1.9130013	-0.9162089
H	4.7589900	-1.9261877	-0.6237493
H	3.7149475	2.7562279	-1.8158956
H	6.9821518	-1.7210223	-1.6387691
H	8.6552584	-0.3372767	-2.8001346
H	8.1597537	2.0191475	-3.3695605
H	5.9806478	3.0129689	-2.7922567
H	-1.9063218	5.5277785	-1.1176934
H	-0.4063493	6.3860157	-0.7201825
H	-1.9260342	4.3704511	0.9911508
H	-0.8482521	5.6712017	1.5314437
H	0.5313136	3.8962279	2.8483320
H	-0.2799400	2.4373220	2.2361795
H	1.4405746	2.7259363	1.8858544
H	0.8457438	5.4095532	-2.8976125
H	0.7872401	3.6397206	-3.0548074
H	-0.6729474	4.6014345	-3.3404588
H	5.9563710	-4.9427411	1.2032957
H	4.7915379	-5.1705261	2.5207371
H	6.5359555	-2.7332342	1.9578457
H	5.8667209	-3.2634948	3.5123124
H	5.1775024	-0.2639080	2.1703915
H	3.4866299	-0.4924165	2.6751125
H	4.7983771	-0.9019880	3.7861111
H	3.9502521	-5.4694890	-0.5345599
H	2.7775674	-5.6308047	0.7899931
H	2.5616387	-4.3727800	-0.4473490

Acknowledgments

The authors gratefully acknowledge continuous financial support by the *Deutsche Forschungsgemeinschaft* (DFG).

16. References

[1] M. Reinmuth, C. Neuhäuser, P. Walter, M. Enders, E. Kaifer, H.-J. Himmel, *Eur. J. Inorg. Chem.* **2011**, 83.
[2] H. Herrmann, E. Kaifer, H.-J. Himmel, *Chem. Eur. J.* **2017**, *23*, 5520.
[3] M. A. Bernstein, S. Sýkora, C. Peng, A. Barba, C. Cobas, *Anal. Chem.* **2013**, *85*, 5778.
[4] H. K. Hansen, C. Riverol, W. E. Acree Jr., *Can. J. Chem. Eng.* **2000**, *78*, 1168.
[5] a) A. Vailaya, *J. Liq. Chromatogr. Relat. Technol.* **2005**, *28*, 965; b) T. Galaon, V. David, *J. Sep. Sci.* **2011**, *34*, 1423; c) M. Tanase, A. Soare, V. David, S. C. Moldoveanu, *ACS Omega* **2019**, *4*, 19808.
[6] A. Ito, M. Uebe, K. Takahashi, H. Ishikawa, D. Sakamaki, H. Sato, T. Matsumoto, K. Tanaka, *Chem. Eur. J.* **2016**, *22*, 2165.
[7] J. Hornung, O. Hübner, E. Kaifer, H. J. Himmel, *RSC Advances* **2016**, *6*, 39323.
[8] a) J. Hankache, O. S. Wenger, *Chem. Rev.* **2011**, *111*, 5138; b) A. Heckmann, C. Lambert, *Angew. Chem. Int. Ed.* **2012**, *51*, 326; c) C. Lambert, C. Risko, V. Coropceanu, J. Rgen Schelter, S. Amthor, N. E. Gruhn, J. C. Durivage, J.-L. Bré, *J. Am. Chem. Soc.* **2005**, *127*, 8508.
[9] a) D. L. Sun, S. V. Rosokha, S. V. Lindeman, J. K. Kochi, *J. Am. Chem. Soc.* **2003**, *125*, 15950; b) M. Yamamoto, Y. Tsujii, A. Tsuchida, *Chem. Phys. Lett.* **1989**, *154*, 559.
[10] a) M. Uebe, T. Kato, K. Tanaka, A. Ito, *Chem. Eur. J.* **2016**, *22*, 18923; b) M. Uebe, K. Kawashima, A. Ito, *Chem. Eur. J.* **2018**, *24*, 16113.
[11] a) K. Deuchert, S. Hünig, *Angew. Chem. Int. Ed.* **1978**, *17*, 875; b) K. Deuchert, S. Hünig, *Angew. Chem.* **1978**, *90*, 927.
[12] a) R. R. Schmidt, *Angew. Chem. Int. Ed.* **1973**, *12*, 212; *Angew. Chem.* **1973**, *85*, 235 b) C. K. Bradsher, *Cationic Polar Cycloaddition, Vol. 16*, Academic Press, **1974**; c) W. Ritzberger-Baumgartner, J. G. Schantl, G. Hajos, *Molecules Online* **1996**, *1*, 119; d) L. R. Domingo, M. J. Aurell, P. Pérez, *RSC Advances* **2014**, *4*, 16567; e) L. R. Domingo, J. A. Sáez, *Org. Biomol. Chem.* **2009**, *7*, 3576.
[13] a) H. Mayr, Armin R. Ofial, J. Sauer, B. Schmied, *Eur. J. Org. Chem.* **2000**, 2013; b) L. R. Domingo, M. Oliva, J. Andrés, *J. Org. Chem.* **2001**, *66*, 6151; c) L. R. Domingo, *J. Org. Chem.* **2001**, *66*, 3211; d) L. R. Domingo, M. Oliva, J. Andrés, *J. Mol. Struct.* **2001**, *544*, 79; e) V. Tamilmani, C. A. Daul, P. Venuvanalingam, *Chem. Phys. Lett.* **2005**, *416*, 354; f) T. Kudoh, T. Mori, M. Shirahama, M. Yamada, T. Ishikawa, S. Saito, H. Kobayashi, *J. Am. Chem. Soc.* **2007**, *129*, 4939; g) V. Tamilmani, D. Senthilnathan, P. Venuvanalingam, *J. Chem. Sci.* **2008**, *120*, 225; h) C. Raquel, A. Juan, *Lett. Org. Chem.* **2011**, *8*, 104; i) L. R. Domingo, M. Ríos-Gutiérrez, P. Pérez, *Org. Biomol. Chem.* **2020**, *18*, 292.
[14] a) K. Fukui, *Theory of orientation and stereoselection*, Springer, Berlin ; Heidelberg, **1975**; b) K. Fukui, *Acc. Chem. Res.* **1971**, *4*, 57; c) I. Fleming, *Molecular orbitals and organic chemical reactions*, Wiley, Chichester, **2011**.
[15] a) K. N. Houk, *Acc. Chem. Res.* **1975**, *8*, 361; b) E. Eibler, P. Höcht, B. Prantl, H. Roßmaier, H. M. Schuhbauer, H. Wiest, J. Sauer, *Liebigs Annalen* **1997**, *1997*, 2471.
